# Musculoskeletal Diseases: Mechanisms and Therapeutic Advances

**DOI:** 10.1002/mco2.70519

**Published:** 2025-12-04

**Authors:** Xiao Ma, Eloy Yinwang, Xupeng Chai, Fangqian Wang, Zehao Chen, Shengdong Wang, Hao Zhou, Yucheng Xue, Jiangchu Lei, Fanglu Chen, Hengyuan Li, Shixin Chen, Shenzhi Zhao, Kelei Wang, Liang Chen, Junjie Gao, Zhaoming Ye, Nong Lin

**Affiliations:** ^1^ Department of Orthopedic Surgery The Second Affiliated Hospital Zhejiang University School of Medicine Hangzhou City Zhejiang Province China; ^2^ Orthopedics Research Institute of Zhejiang University Hangzhou City Zhejiang Province China; ^3^ Key Laboratory of Motor System Disease Research and Precision Therapy of Zhejiang Province Hangzhou City Zhejiang Province China; ^4^ Clinical Research Center of Motor System Disease of Zhejiang Province Hangzhou City China; ^5^ Department of Orthopedics Shanghai Sixth People's Hospital Affiliated to Shanghai Jiao Tong University School of Medicine Shanghai China

**Keywords:** bone and soft tissue tumors, immune modulation, inflammation, musculoskeletal diseases, precision immunotherapy

## Abstract

Musculoskeletal diseases encompass a broad spectrum of inflammatory, degenerative, and neoplastic disorders that compromise bone and joint function across the lifespan. Increasing evidence highlights the central role of immune regulation in their pathogenesis, driven by complex interactions among immune, bone, and stromal cells. Inflammatory conditions such as rheumatoid arthritis, ankylosing spondylitis, and dermatomyositis are marked by persistent immune activation and progressive tissue destruction. Degenerative diseases like osteoarthritis, osteoporosis, and intervertebral disc degeneration involve immune senescence, dysregulated tissue remodeling, and inflammation‐driven structural damage. Bone and soft tissue tumors—including osteosarcoma, chondrosarcoma, Ewing sarcoma, and soft tissue sarcoma—develop within immunosuppressive niches that hinder antitumor immunity. Notably, these immune environments are not strictly dichotomous but exhibit dynamic, context‐dependent states of immune stimulation and suppression. This review delineates both shared and disease‐specific immune mechanisms, spanning cytokine networks, signaling pathways, and cellular interactions. It further discusses current and emerging therapeutic strategies, including cytokine modulators, bone‐regulatory agents, immune checkpoint inhibitors, and cell‐based therapies. Despite recent advances, key challenges persist in translating immunological insights into durable, disease‐modifying treatments. By bridging mechanisms across inflammation, degeneration, and malignancy, this review provides an integrated framework for understanding immune contributions to musculoskeletal diseases and identifies promising directions for precision immunotherapy.

## Graphical abstract

Musculoskeletal diseases comprise a broad spectrum of inflammatory, degenerative, and neoplastic disorders. Increasing evidence highlights the central role of immune regulation in their pathogenesis, with complex interactions among immune, bone, muscle, and stromal cells. The immune environments are not strictly dichotomous but exhibit dynamic and context‐dependent immune stimulation and suppression. By bridging mechanisms across inflammation, degeneration, and malignancy, this review provides an integrated framework for understanding immune contributions to musculoskeletal diseases and identifies promising directions for precision immunotherapy.

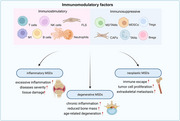

## 1 Introduction

Musculoskeletal diseases (MSDs) encompass a spectrum of conditions affecting the bones, joints, and associated connective tissues, leading to functional impairment and disability, and imposing a substantial economic burden on nearly 2 billion people worldwide [1]. These disorders, including inflammatory, degenerative, and neoplastic conditions, disrupt the maintenance of bone homeostasis, growth, and regeneration, processes that depend critically on a delicate balance between bone formation and resorption [2, 3]. The immune system plays a crucial role in the pathogenesis and progression of a wide range of MSDs. For example, in inflammatory disorders, such as rheumatoid arthritis (RA) and ankylosing spondylitis (AS), excessive immune activation and subsequent autoimmune attacks on joint tissues contribute to pain, swelling, and progressive structural destruction [4, 5]. In degenerative diseases like osteoarthritis (OA) and osteoporosis, the synchronization between proinflammatory cytokines and osteoclast differentiation accelerates the loss of bone integrity [6]. In neoplastic diseases, such as osteosarcoma and soft tissue sarcoma (STS), the compromised immune response promotes the rapid proliferation of cancer cells, thereby fostering tumor progression and tissue destruction [6]. Therefore, understanding the complex interplay between the immune and skeletal systems is important for developing effective strategies to address bone‐ and muscle‐related disorders and improve therapeutic outcomes.

Historically, the relationship between the bone and immune systems was first recognized in the 1970s, when researchers noticed that immune cell‐derived factors could potentiate osteoclast development, highlighting the participation of the immune system in bone metabolism [7]. Later, in the 2000s, the concept of *osteoimmunology* was put forward by H. Takayanagi to describe their mutual influence and complex crosstalk [8, 9]. The exploration of the interplay between muscle tissues and the immune system was marked by the refinement of experimental autoimmune myositis models in the 20th century [10], whereas research on tendon–immune interactions was advanced later, focusing primarily on inflammation and tissue repair in tendinopathies [11]. Currently, evaluating the immune status has become essential in the study of MSDs, as the immune and musculoskeletal systems share a variety of cytokines and cellular components. One such key cytokine is receptor activator of nuclear factor κB (RANK) ligand (RANKL), initially identified as a mediator of T cell proliferation and dendritic cell (DC) function, but now primarily recognized for its importance in bone biology [12]. Osteoclasts, which derive from the monocyte/macrophage lineage and are stimulated by RANKL during early stages of differentiation [13], play key roles in bone metabolism. Importantly, the crosstalk between tissue and immune cells is bidirectional. On the one hand, immune cells such as macrophages, natural killer (NK) cells, DCs, and T cells play crucial roles in modulating the physiological functions of bone tissue cells. On the other hand, bone, muscle, and tendon cells also influence the recruitment, survival, and functionality of immune cells [14]. As research advances and more evidence are discovered, the intricate connections between the two systems will be further uncovered, deepening our understanding of this field.

Despite the growing body of knowledge, there remains a lack of comprehensive understanding of the intricate relationship between the two systems. Therefore, in this review, we summarize the functions of various immunological factors, with a primary focus on cellular components, in MSDs, as well as the latest therapeutic advances utilizing immune modulation strategies. Given the differences in pathogenesis, we have organized the review into four main sections: the physiology of bone, muscle, and tendon tissues, as well as their involvement in inflammatory, degenerative, and neoplastic conditions. Understanding the mechanisms underlying destructive immune storms and permissive immune deserts is crucial for designing novel, safe, and efficient therapeutic strategies.

## 2 Physiological Musculoskeletal Microenvironment

The physiological musculoskeletal microenvironment comprises bone, muscle, and tendon tissues, together with resident immune cells and the extracellular matrix (ECM). These components engage in dynamic biochemical and biomechanical interactions to maintain homeostasis and regenerative capacity. Understanding their coordinated roles under normal conditions provides the foundation for elucidating the mechanisms underlying musculoskeletal pathologies.

### 2.1 Physiological Bone Microenvironment

#### 2.1.1 Bone Cells

The bone microenvironment is a highly organized and dynamic niche composed of mesenchymal and hematopoietic cells, immune cells, ECM, bone marrow stroma, a rich vascular network, and a diverse array of cytokines, growth factors, and hormones [15]. Major bone‐forming cells comprise osteocytes, bone lining cells, osteoblasts, and osteoclasts. Osteocytes, embedded within the mineralized bone matrix, play a central regulatory role in bone remodeling by communicating with osteoclasts and osteoblasts via signaling pathways including RANKL/OPG (osteoprotegerin) and Sost/Dkk1/Wnt (sclerostin/dickkopf‐related protein 1/wingless‐related integration site) [16]. Osteoblasts, the principal bone‐forming cells, originate from bone marrow‐resident mesenchymal stem cells (MSCs) through intermediate stages of osteo‐chondroprogenitors and osteoprogenitors [17, 18, 19], a process primarily regulated by the BMPs (bone morphogenic proteins) and Wnt signaling pathways [12, 20]. Mature osteoblasts may further differentiate into osteocytes and become bone lining cells—the latter representing quiescent osteoblasts lining the bone surface [16]. In contrast, osteoclasts are multinucleated, bone‐resorbing cells derived from the monocyte/macrophage lineage through the fusion of preosteoclasts [21, 22]. Osteoclastogenesis is tightly regulated by osteoblasts and their progenitors through osteoblast‐derived cytokines such as RANKL, OPG, and macrophage colony stimulating factor (M‐CSF) [12, 20]. Together, these bone cells are indispensable for maintaining skeletal structure and integrity, ensuring the continuous renewal of bone tissues through coordinated matrix resorption and new bone formation. The interplay among osteocytes, osteoblasts, and osteoclasts orchestrates bone turnover and preserves skeletal integrity under physiological conditions.

#### 2.1.2 Immune Cells

Under physiological conditions, B and T lymphocytes represent the primary immune populations modulating bone remodeling [12]. B cells, which depend on the bone marrow microenvironment for their maturation [23], serve as the main producers of OPG within the bone marrow [24]. Acting as a decoy receptor for RANKL, OPG inhibits osteoclastogenesis and preserves bone mass. T cells support B cell function by expressing costimulatory surface molecules such as CD40L (CD40 ligand) [24], and also contribute to the suppression of osteoclast differentiation by expressing CTLA4 (cytotoxic T‐lymphocyte antigen 4), which induces tryptophan degradation and apoptosis of osteoclast precursors (OCPs) via CD80/86 binding [25].

The contribution of T cells to bone turnover is multifaceted, reflecting the functional diversity among their subsets. Th2 cells (T helper cell type 2), for example, activate B cells to facilitate bone regeneration [26], while Th17 cells promote bone resorption both directly—by secreting IL‐17 (interleukin‐17), IL‐22, and IL‐26—and indirectly—by recruiting other osteoclastic immune cells [23]. Additionally, regulatory T cells (Tregs), a specialized immunosuppressive subset characterized by expression of the transcription factor Forkhead Box P3 (Foxp3) [27], are physiologically promoted by bone‐residing Langerhans cells [28]. Tregs consist of thymus‐derived natural Tregs (nTregs), which primarily suppress immune responses via direct cell‐contact mechanisms to inhibit self‐reactive T cells, and peripherally induced Tregs (iTregs), which mainly exert their suppressive functions by secreting immunosuppressive cytokines such as IL‐10 and TGFβ (transforming growth factor beta) [29]. Both subsets rely on IL‐2 and TGFβ for their development and maintenance [30]. In addition, Tregs sustain bone homeostasis by inhibiting osteoclastogenesis via secretion of TGFβ, IL‐4, and IL‐10, and through direct CTLA4‐CD80/86 interactions that induce osteoclast apoptosis [29]. Interestingly, osteoclasts can also activate Tregs, suggesting the existence of a negative feedback loop [31]. Importantly, the balance between Th17 cells and Tregs is particularly crucial for preserving bone mass [32]. Of note, a noncanonical Treg subset, capable of secreting osteolytic cytokines IL‐17 and RANKL, has been identified, highlighting the nuanced and context‐dependent roles of Tregs in bone physiology [33]. Taken together, disruption of B–T cell cooperation or imbalance among T cell subsets perturbs osteoclastogenesis and ultimately drives pathological bone loss.

In addition to B and T cells, nonosteoclastic macrophages have garnered increasing attention for their extensive involvement in bone immunity and regulation of regenerative processes [23]. Bone‐resident macrophages, also known as “OsteoMacs,” are strategically located along bone surfaces in proximity to mature osteoblasts [34]. Their remarkable plasticity allows them to polarize into distinct functional phenotypes. The M1 phenotype exerts antiosteogenic effects by secreting grancalcin, a Ca^2+^‐binding protein that binds the Plexin‐B2 receptor, thereby suppressing osteoblastogenesis and promoting adipogenic conversion of bone marrow stromal cells [35]. Conversely, M2 macrophages facilitates tissue repair and osteoblast mineralization through production of cytokines like TGFβ, IL‐10, and arginase [36]. Macrophages also play a pivotal role in the endosteal maintenance of hematopoietic stem cells, ensuring hematopoiesis [37]. In addition, they are also crucial for angiogenesis through production of vascular endothelial growth factor (VEGF) and platelet‐derived growth factor, which help maintain adequate blood supply essential for bone health and regeneration [38]. In summary, macrophages play an indispensable role in bone homeostasis, orchestrating immune regulation, tissue repair, and matrix remodeling in a tightly regulated manner.

#### 2.1.3 Bone ECM

The bone ECM is a highly organized and hierarchically structured composite of both inorganic and organic components. The inorganic portion is predominantly made up of hydroxyapatite (HA, Ca_5_(PO_4_)_3_OH), which is deposited along the collagen framework to confer rigidity and mechanical strength to the bone [39]. In contrast, the organic matrix—including collagens, proteoglycans, γ‐carboxyglutamic acid‐containing proteins, glycoproteins, and small integrin‐binding ligand N‐linked glycoproteins [40]—mainly regulates mineralization and metabolic processes.

Type I collagen, the predominant collagen in bone, is composed of two α1 chains and one α2 chain, encoded by the *Col1a1* and *Col2a1* genes, respectively. These chains form a triple‐helical structure that further assembles into collagen fibrils and fibers in a hierarchical fashion [41]. The unmineralized form of collagen, known as osteoid in bone tissues, is intimately linked to its mineralized counterparts and serves as the scaffold for subsequent mineral deposition by osteoblasts [42]. Dysfunction or deficiency of type I collagen significantly increases the risk of fractures, highlighting its essential role in skeletal integrity [43]. Type III and IV collagens, though less abundant, modulate the organization and function of type I collagen, affecting fibril diameter and fibrillogenesis [40].

Noncollagenous proteins (NCPs) also play vital roles in maintaining the functional integrity of the ECM. Among them, small leucine‐rich proteoglycans (SLRPs) such as biglycan and decorin represent a major class. These secreted proteins are characterized by glycosaminoglycan residues [44] and a protein core composed of leucine‐rich repeats (LRRs) [45], enabling them to bind ligands such as TGFβ, collagens, and fibronectin through β‐sheet‐mediated interactions. Through these interactions, SLRPs regulate cell–matrix interactions, cell proliferation and bone remodeling [46]. Two other key NCPs are bone sialoprotein (BSP) [47] and osteopontin (OPN) [48], which play opposing roles in regulating mineralization: BSP promotes, while OPN inhibits, bone formation and mineral deposition.

In summary, the bone ECM is a multifaceted and dynamic system of organic and inorganic components that collectively ensure proper bone structure, function, metabolism, and turnover. A deeper understanding of its composition and regulatory mechanisms highlights the therapeutic potential of targeting ECM components to treat matrix‐related bone disorders.

### 2.2 Physiological Muscle Microenvironment

#### 2.2.1 Muscle Cells

The muscle microenvironment constitutes of muscle cells, immune cells, and a noncellular ECM, which together maintain tissue homeostasis and preserve the body's muscle mass. Muscle‐forming cells primarily refer to myofibers, which can be classified into two functional subtypes: slow‐twitch (type I) fibers that are predominantly oxidative, and fast‐twitch (type II) fibers that rely on glycolytic metabolism [49]. Studies have also revealed proliferative differences between these subtypes, showing that fast‐twitch fibers display higher proliferative efficiency but lower self‐renewal potential [50]. Satellite cells, residing beneath the basal lamina, act as a reservoir for muscle regeneration following injury. In adult skeletal muscle tissues, the satellite cell niche represents a well‐defined anatomical compartment located between the myofiber plasma membrane and a laminin‐rich basal lamina, maintains satellite cells in a quiescent state but becomes activated upon homeostatic disruption, during which immune cells infiltrate, inflammatory cytokines are released, satellite cells proliferate, and the ECM undergoes remodeling [51]. In contrast, immature niches within rapidly growing or regenerating muscle contain immature myofibers and a loosely organized basal lamina, providing a permissive environment for the rapid expansion of muscle progenitor cells [52].

#### 2.2.2 Immune Cells

Although muscle tissues lack defined anatomical or functional barriers, they are generally regarded as a nonclassical immune‐privileged site, where immune responses remain largely quiescent and the expression of MHC (major histocompatibility complex) molecules is minimal unless injury or regeneration occurs [53]. Under physiological conditions, macrophages participate in the clearance of damaged tissue while simultaneously releasing mediators that regulate satellite cell and fibroblast proliferation and enhance vascular permeability—events that collectively set the stage for muscle regeneration. During the regenerative phase, M2‐polarized macrophages dominate the immune landscape, promoting myogenesis and collagen deposition [54]. Similarly, neutrophils play a dual role: they combat inflammation but can also contribute to muscle injury through the release of free radicals and proteolytic enzymes [55]. In contrast, lymphoid cells have historically received less attention in studies of the physiological muscle microenvironment, partly due to their rare presence in physiological muscles, though recent research has begun to highlight their importance. For example, Tregs are now recognized as essential for effective muscle regeneration, and impairment of IL‐6Rα (IL‐6 receptor alpha) signaling has been shown to cause significant muscle loss [56]. In summary, muscle tissue remains immunologically quiescent under normal conditions; however, once homeostasis is disrupted, immune activation profoundly shapes both tissue damage control and regenerative myogenesis, underscoring the indispensable role of immune regulation in muscle physiology.

#### 2.2.3 Muscle ECM

The muscle ECM provides not only structural support but also biochemical cues for muscle cell alignment, adhesion, and signaling. Typically, the muscle ECM is organized into three hierarchical layers: the epimysium, perimysium, and endomysium, which enclose the entire muscle, each muscle fascicle, and each individual myofiber, respectively [57]. The ECM of skeletal muscle is composed primarily of collagen type I, forming the main fibrous network that confers mechanical strength and stability, together with other fibril‐forming collagens such as types III, V, IX, and XI. Nonfibrillar collagens, including type IV and VI, also play essential roles in maintaining the integrity and connectivity of the muscle structural network [58]. Importantly, the proper organization of these ECM components is critical for preserving muscle architecture and function. In aging‐related sarcopenia, excessive crosslinking of collagen type I fibers causes enhanced tissue stiffness, fibrosis, and impaired muscle performance [59]. Deficiency of collagen type IV disrupts the ECM–TGFβ signaling homeostasis, leading to muscle fiber dysfunction and muscular dystrophies [60]. Moreover, ECM remodeling is an essential, tightly regulated process during regenerative myogenesis driven by satellite cells. For instance, impaired deposition of laminin‐α1 and laminin‐α5 into the basal lamina of the satellite cell niche profoundly compromises satellite cell expansion and self‐renewal [61]. In conclusion, the muscle ECM functions as a dynamic and instructive scaffold—maintaining tissue integrity, mediating mechanical and metabolic interactions, and orchestrating the physiological and regenerative processes of muscle tissues.

### 2.3 Physiological Tendon Microenvironment

#### 2.3.1 Tendon Cells

Tendons are dense, low‐metabolic connective tissues that connect muscles to bones, enabling force transmission and coordinated body movement. Depending on their anatomical and functional properties, tendons can be categorized into stiff, positional tendons that stabilize joints, and elastic, energy‐storing tendons that facilitate efficient motion [62]. While the ECM constitutes the major structural component of tendons, tendon‐resident cells, particularly tenocytes, are essential for the maintenance and renewal of the tissue. These cells exhibit a highly elongated morphology aligned with collagen fibrils and tensile stress directions [63], and display low metabolic activity [64], reflecting their role in sustaining the dense collagenous architecture. In addition, a smaller population of tendon stem/progenitor cells (TSPCs) resides within tendon tissues, contributing to tendon healing by differentiating into tenocytes, providing key regulatory cues in tendon regeneration [65].

#### 2.3.2 Immune Cells

Although tendon tissues are generally regarded as immunologically quiescent, they nevertheless harbor several types of tissue‐resident immune cells, such as macrophages and mast cells. Macrophages, encompassing both M1‐ and M2‐like phenotypes, are responsible for immune surveillance and clearance of apoptotic cells, thereby maintaining the balance of tissue remodeling [66]. Tendon‐resident macrophages also play a regulatory role in modulating fibroblast phenotype and ECM organization (ECMO) during tendon growth [67]. Moreover, a distinct population of tissue‐resident macrophage‐like cells, termed “tenophages,” has also been identified under physiological conditions; these CX3CL1⁺/CX3CR1⁺ cells represent tendon‐specific extensions of the macrophage surveillance network [68]. Mast cells, although comparatively sparse, reside in healthy tendons as well as in adjacent connective tissues near the paratenon, muscle–tendon junction, and bone–tendon junction [69]. These cells contain granules rich in inflammatory mediators and proteolytic enzymes, ready to be activated upon tendon overload [70]. Lymphocytes are even more sparsely distributed in healthy tendon tissues and are mostly studied under pathological or injury conditions [71]. In summary, the macrophage‐dominant immune landscape of normal tendons supports tissue homeostasis while maintaining readiness for potential pathological challenges involving active immune infiltration.

#### 2.3.3 Tendon ECM

The ECM of tendons plays a significant role in preserving the tensile architecture, strength, elasticity, and overall mechanical integrity. Collagens constitute the predominant component of the tendon ECM, with collagen type I forming the primary fibrous framework and smaller proportions of collagens type III, V and XI contributing to fibril organization [62]. Under physiological conditions, collagen molecules are assembled into hierarchically organized fiber bundles, crosslinked at their N‐ and C‐termini, and aligned in parallel with the principal direction of tensile stress [72]. In contrast, the noncollagenous matrix compartments, which provide a permissive microenvironment for nutrient exchange and innervation within the dense tendon structure, are enriched in proteoglycans (mainly SLRPs), elastin, and glycoproteins [73]. Importantly, the tendon ECM also underlies the tissue's mechanical adaptability, which relies on mechanotransduction pathways that convert mechanical stimuli into cellular responses, thereby regulating biochemical synthesis and matrix remodeling [74]. In brief, these components form a highly ordered yet dynamic matrix that confers mechanical resilience and structural integrity, ensuring the proper function of the musculoskeletal system.

## 3 Inflammatory MSDs

Inflammatory MSDs are a group of conditions characterized by a shared pathogenic mechanism: the breakdown of immune tolerance, which leads to dysregulated immune responses that mistakenly target self‐tissues, causing inflammation and tissue damage. This chapter explores the specific immunological components underlying the pathology of representative disorders, including RA, AS, psoriatic arthritis (PsA), dermatomyositis (DM), and polymyositis (PM).

### 3.1 Rheumatoid Arthritis

**FIGURE 1:**
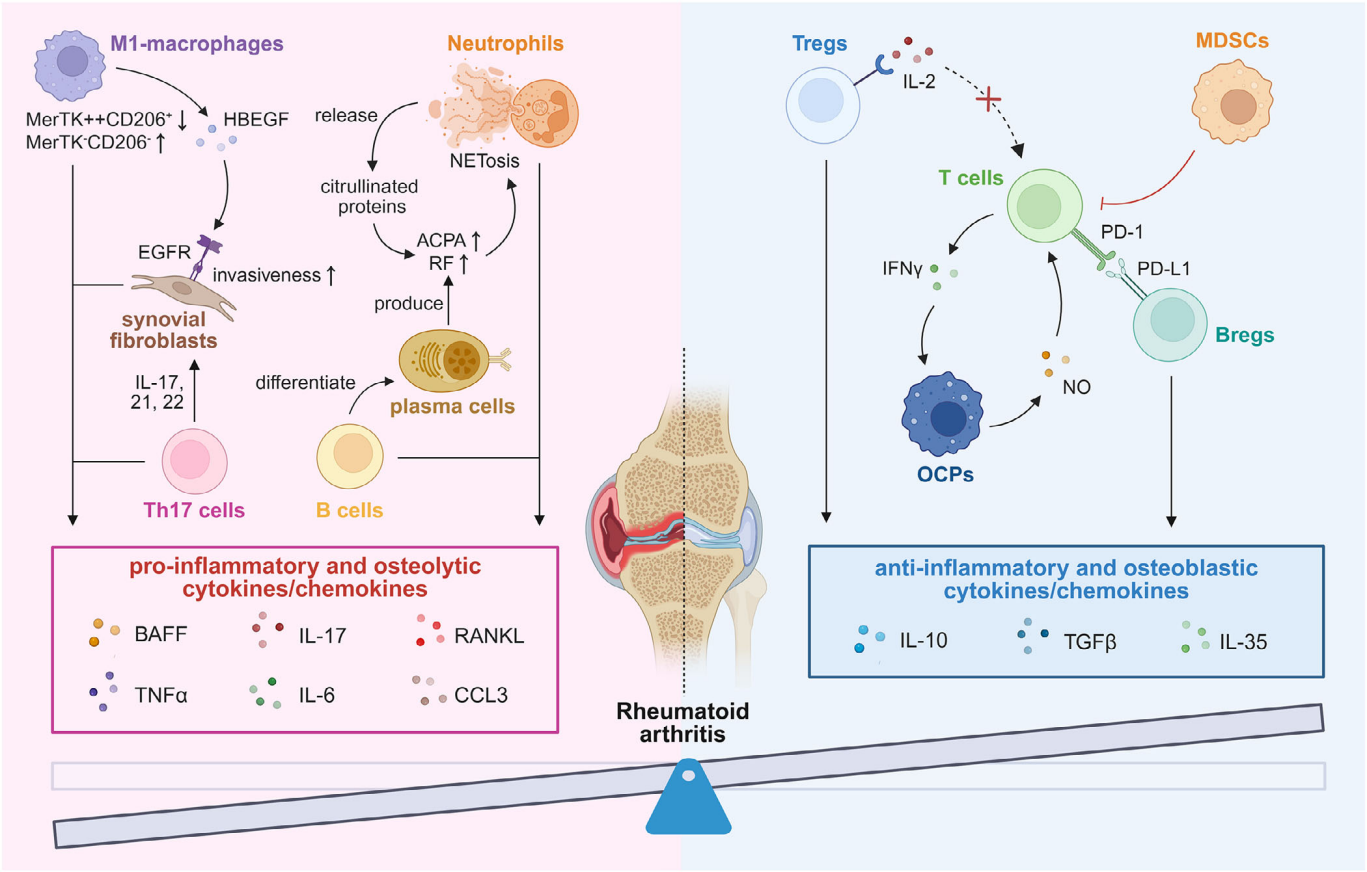
Imbalanced immune responses in the rheumatoid arthritis microenvironment. M1 macrophages secrete IL‐6 and TNFα, promoting inflammation and joint destruction. MerTK⁺CD206⁺ resident macrophages reduced; MerTK^−^CD206^−^ infiltrating macrophages expanded during active RA. HBEGF⁺ macrophages enhance fibroblast invasiveness via EGFR signaling. Neutrophils release NETs, expose citrullinated antigens, amplify autoantibody production. Neutrophils produce TNFα, BAFF, and RANKL, contributing to bone erosion. B cells produce TNFα and CCL3 to suppress bone formation; TNFα and RANKL drive osteoclastogenesis. Plasma cells produce RF and ACPAs, forming immune complexes and triggering complement‐mediated joint damage. Th17 cells secrete IL‐17, IL‐21, IL‐22, upregulating RANKL on synovial fibroblasts and activating OCPs. CD11b^−/low^CD115⁺CD117⁺ MDSC–OCP hybrids suppress CD4⁺ and CD8⁺ T cells via nitric oxide; suppression depends on T cell‐derived IFNγ. Tregs produce IL‐10 and TGFβ; express CD25 to consume IL‐2 and suppress effector T cells. Bregs secrete IL‐10, IL‐35, and TGFβ; express PD‐L1 to inhibit T cell activation. *Abbreviations*: TNFα, tumor necrosis factor alpha; MerTK, Mer tyrosine kinase; HBEGF, heparin‐binding EGF‐like growth factor; EGFR, epidermal growth factor receptor; NETs, neutrophil extracellular traps; BAFF, B cell‐activating factor; RANKL, receptor activator of nuclear factor κB ligand; RF, rheumatoid factor; ACPAs, anticitrullinated protein antibodies; OCPs, osteoclast precursors; MDSC, myeloid‐derived suppressor cell; IFNγ, interferon gamma; TGFβ, transforming growth factor beta; PD‐L1, programmed death‐ligand 1.

RA is a chronic systemic inflammatory disease primarily characterized by persistent synovitis, cartilage destruction, and excessive systemic inflammation [75]. Dysregulated autoimmune responses are a hallmark of RA. Diagnosis of RA is based on clinical manifestations, and is also supported by the evidence of systemic inflammation such as increased erythrocyte sedimentation rate (ESR) or C‐reactive protein (CRP) [76]. In contrast, the presence of immunosuppressive factors, such as myeloid‐derived suppressor cells (MDSCs), Tregs, and regulatory B cells (Bregs), may act as protective modulators in the microenvironment. Hence, the profound involvement of the immune system is of major importance in RA (Figure 1).

#### 3.1.1 Immuno‐stimulatory Factors in RA

##### 3.1.1.1 Macrophages

Macrophages exhibit remarkable plasticity and can polarize into either proinflammatory (M1‐like) or anti‐inflammatory (M2‐like) phenotypes in response to microenvironment cues, with RA favoring M1 polarization. Studies have demonstrated a positive correlation between the abundance of synovial macrophages and the severity of joint damage [77]. The activated M1‐polarized macrophages produce a range of proinflammatory cytokines, such as IL‐6 and TNFα (tumor necrosis factor alpha), that drive tissue inflammation and destruction [77, 78, 79].

Beyond the classical M1/M2 paradigm, recent studies have identified a phenotypic distinction between MerTK⁺CD206⁺ tissue‐resident macrophages—dominant in healthy synovium—and MerTK^−^CD206^−^ tissue‐infiltrating macrophages, which markedly expand during active RA and contribute to local inflammation [77]. A specific subgroup of HBEGF^+^ macrophages enriched in RA synovium has also been shown to promote fibroblast invasiveness via epidermal growth factor receptor (EGFR)‐dependent mechanisms [80]. These results underscore the diverse and pathogenic roles of macrophage subtypes in RA‐associated immune dysregulation. Additionally, recent research has revealed that high‐salt diets can induce macrophage pyroptosis through sodium transport [81], highlighting the potential of lifestyle interventions in RA management.

Taken together, these findings underscore the central role of macrophages in RA pathogenesis and highlight macrophage‐targeted modulation as a promising strategy for attenuating synovial inflammation.

##### 3.1.1.2 Neutrophils

Neutrophils, as frontline effectors of the innate immune system, function through multiple mechanisms, including phagocytosis, degranulation, the generation of reactive oxygen species (ROS) production, and the formation of neutrophil extracellular trap (NETs) [82, 83]. Upon activation by proinflammatory signals, neutrophils exhibit a wide range of effector functions shaped by their surface receptor repertoire and intracellular protein profiles. In RA, neutrophils are pathologically hyperactivated and frequently release NETs—web‐like extracellular structures primarily composed of nucleic acids complexed with nuclear, cytoplasmic, and granule proteins—through a specialized form of cell death known as NETosis [83, 84]. RA‐associated autoantibodies, including anticitrullinated protein antibodies (ACPAs) and rheumatoid factor (RF), as well as proinflammatory cytokines such as IL‐17A and TNFα, have been shown to trigger NET formation. In turn, NETs expose citrullinated proteins, perpetuating a self‐amplifying cycle of autoantibody production and inflammation. They also induce the expression of proinflammatory genes in fibroblast‐like synoviocytes (FLSs), thereby amplifying inflammatory responses in the RA synovium. Beyond NETosis, neutrophils in synovial joints also contribute to RA pathogenesis by producing cytokines such as TNF and BAFF (B cell‐activating factor), as well as signaling molecules such as RANKL, together with various chemokines and their receptors, which collectively drive local autoantibody production, bone erosion, and sustained inflammation [84].

##### 3.1.1.3 B Cells

The proliferation, differentiation, and activation of B cells are orchestrated by multiple factors, including dual signaling through B cell receptor (BCR) engagement and costimulatory signals, cytokines such as IL‐12, and essential survival factors like BAFF and APRIL (a proliferation‐inducing ligand) [85]. Accumulating evidence has highlighted the central role of B cells, particularly double‐negative (CD27^−^IgD^−^) and class‐switched memory (CD27^+^IgD^−^) B cells, in the pathogenesis of RA. B cells contribute to disease progression through multiple interconnected mechanisms, including antigen presentation, cytokine‐driven inflammation, and autoantibody‐mediated tissue damage. First, in proteoglycan‐induced arthritis (PGIA), a classic murine model of RA, B cell deficiency impairs the activation of T cells and thereby prevents disease onset, underscoring the indispensable role of B cells in priming autoreactive T cell responses [86]. Second, B cells secrete a spectrum of proinflammatory cytokines, including TNFα, IFNγ (interferon gamma), IL‐6, IL‐1β, and IL‐17, which actively promote joint inflammation and bone destruction [85]. For example, TNFα and CCL3 produced by B cells suppress bone formation in RA patients, whereas TNF and RANKL promote osteoclast differentiation and excessive bone resorption [87]. Third, upon differentiation into plasma cells, B cells produce autoantibodies such as RF and ACPAs. These autoantibodies form immune complexes that activate the complement cascade, resulting in joint injury [88]. In summary, B cells orchestrate RA progression through multifaceted mechanisms ranging from adaptive immune activation to direct mediation of inflammatory and osteo‐destructive processes.

##### 3.1.1.4 Th Cells

Th cells, a subset of CD4^+^ T lymphocytes, encompass well‐defined effector populations such as Th1, Th2, and Th17 cells, as well as regulatory subsets like Tregs [30]. Activated Th cells express RANKL along with a range of effector cytokines with either stimulatory or inhibitory effects on osteoclastogenesis [9, 89, 90]. Among them, Th1 and Th17 cells play pivotal roles in driving autoimmune inflammation. Th1 cells mediate IFNγ‐dependent macrophage activation and osteoclastogenesis. Th17 cells produce IL‐17, IL‐21, and IL‐22, which upregulate RANKL expression on synovial fibroblasts and subsequently activate OCPs, contributing to bone erosion [91]. Notably, IL‐17 has also been implicated in osteoblast regulation, although its role remains controversial—some studies report inhibition of calvarial osteoblast differentiation [92], whereas others suggest a pro‐osteogenic effect [93]. The developmental plasticity and interplay between Th17 and Tregs have emerged as a key focus in autoimmune research, as the Th17/Treg ratio is increasingly recognized as a prognostic indicator in RA [30, 94]. A more detailed discussion of the role of Tregs is provided in subsequent sections. Taken together, these heterogeneous T cell subsets contribute dynamically to the immunopathogenesis of RA by modulating both inflammatory responses and bone remodeling processes, and should be carefully considered in clinical strategies aimed at preserving bone mass and structural integrity.

#### 3.1.2 Immuno‐Suppressive Factors in RA

##### 3.1.2.1 MDSCs

MDSCs represent a heterogeneous population of immature myeloid cells characterized by potent immunosuppressive functions [95, 96]. They retain the capacity to differentiate into macrophages, monocytes and DCs [97]. Based on their derivation from granulocytic or monocytic myeloid lineages, MDSCs are categorized as CD11b^+^Ly6C^low^Ly6G^+^CD49d^−^ polymorphonuclear/granulocytic MDSCs (PMN‐MDSCs/G‐MDSCs) and CD11b^+^Ly6C^high^Ly6G^−^CD49d^+^ monocytic MDSCs (M‐MDSCs), with the two subgroups sharing certain phenotypical and morphological features [98]. Studies using collagen‐induced inflammatory arthritis (CIA) model, which recapitulates key features of RA pathogenesis, suggest that artificially expanded MDSCs can attenuate disease progression [99], whereas their depletion exacerbates it [100]. Moreover, a specialized subset of CD11b^–/low^CD115^+^CD117^+^ MDSCs exhibiting dual phenotypic characteristics of MDSCs and OCPs has been shown to suppress the proliferation of both CD4^+^ and CD8^+^ T cells in vitro via nitric oxide production, further underscoring their immunomodulatory capacity [101]. Notably, this OCP‐mediated T cell suppression is dependent on T cell‐derived IFNγ, positioning these specialized MDSCs as a “guarding system” that helps prevent immune hypersensitivity.

Conversely, some studies have reported proinflammatory effects of MDSCs in RA, mediated via production of BAFF that stimulates B cells to secrete TNFα via the BTK (Bruton's tyrosine kinase)/NF‐κB signaling pathway [102]. These findings collectively illustrate the complex roles of MDSCs in RA pathogenesis, with an overall predominance of immunosuppressive activity. Modulating MDSC function may also alleviate RA‐induced bone loss, as certain subsets possess the potential to further differentiate into osteoblasts.

##### 3.1.2.2 Tregs

Tregs, a distinct immunosuppressive subset of T cells, play a crucial protective role against various autoimmune diseases. In RA, however, the frequency and function remain controversial. Some studies report reduced frequencies of protective Tregs in RA [103], with disease improvement observed following their stimulation—such as via upregulation of aryl hydrocarbon receptor transcription factor [104] and TNFR2 (TNF receptor 2) [105]. Conversely, a more recent study indicates increased Treg numbers during RA flares compared with remission, yet these Tregs display impaired regulatory capacity, characterized by decreased expression of CD39, FOSB, and TRBC2 (T cell receptor [TCR] beta constant 2), alongside increased expression of interferon‐induced transmembrane protein 2 [106]. Functional defects in RA Tregs include decreased production of IL‐10 and TGFβ, along with abnormalities in CTLA4 and the IL‐2 receptor CD25, which collectively compromise their ability to suppress effector T cell activity [107, 108]. Additionally, atypical Treg subtypes, such as Th17‐like Tregs [109] and eosinophil‐suppressing Tregs expressing IL‐33 receptor [110], may paradoxically exacerbate RA pathology. These complex and sometimes contradictory roles underscore the need for precise characterization of Tregs in developing Treg‐based immunotherapies for RA.

##### 3.1.2.3 Bregs

Bregs represent a modulatory subgroup of CD19^+^CD24^high^CD38^high^ B cells that play pivotal role in maintaining immune tolerance and restraining excessive inflammation [111, 112]. Their suppressive functions on T cells and other proinflammatory immune cells are mainly mediated through the secretion of cytokines like IL‐10, IL‐35, and TGFβ [113]. In RA, reduced numbers and impaired function of IL‐10‐producing Bregs—commonly observed in patients—are associated with uncontrolled inflammation and aggravated disease activity [114, 115, 116, 117, 118, 119] and the presence of RF [120]. However, some studies have reported contradictory findings [121, 122, 123], although higher levels of Breg in these cohorts still correlated with improved therapeutic responses [121]. The protective role of Bregs in RA is primarily attributed to the secretion of IL‐10, a potent T cell suppressor triggered by pathways involving ERK (extracellular signal‐regulated kinase), p38, cyclic adenosine monophosphate (cAMP) response element binding protein, and pSTAT3 (phosphorylated signal transducer and activator of transcription 3) [115]. Additionally, the expression of IL‐21 [116], granzyme B (GZMB) [116, 117], PD‐L1 [118], and Treg‐specific Foxp3 [124] also contribute to Breg‐mediated immunoregulation in RA. In summary, Breg‐mediated immune tolerance is crucial for maintaining joint homeostasis and preventing inflammatory destruction in RA.

### 3.2 Ankylosing Spondylitis

**FIGURE 2:**
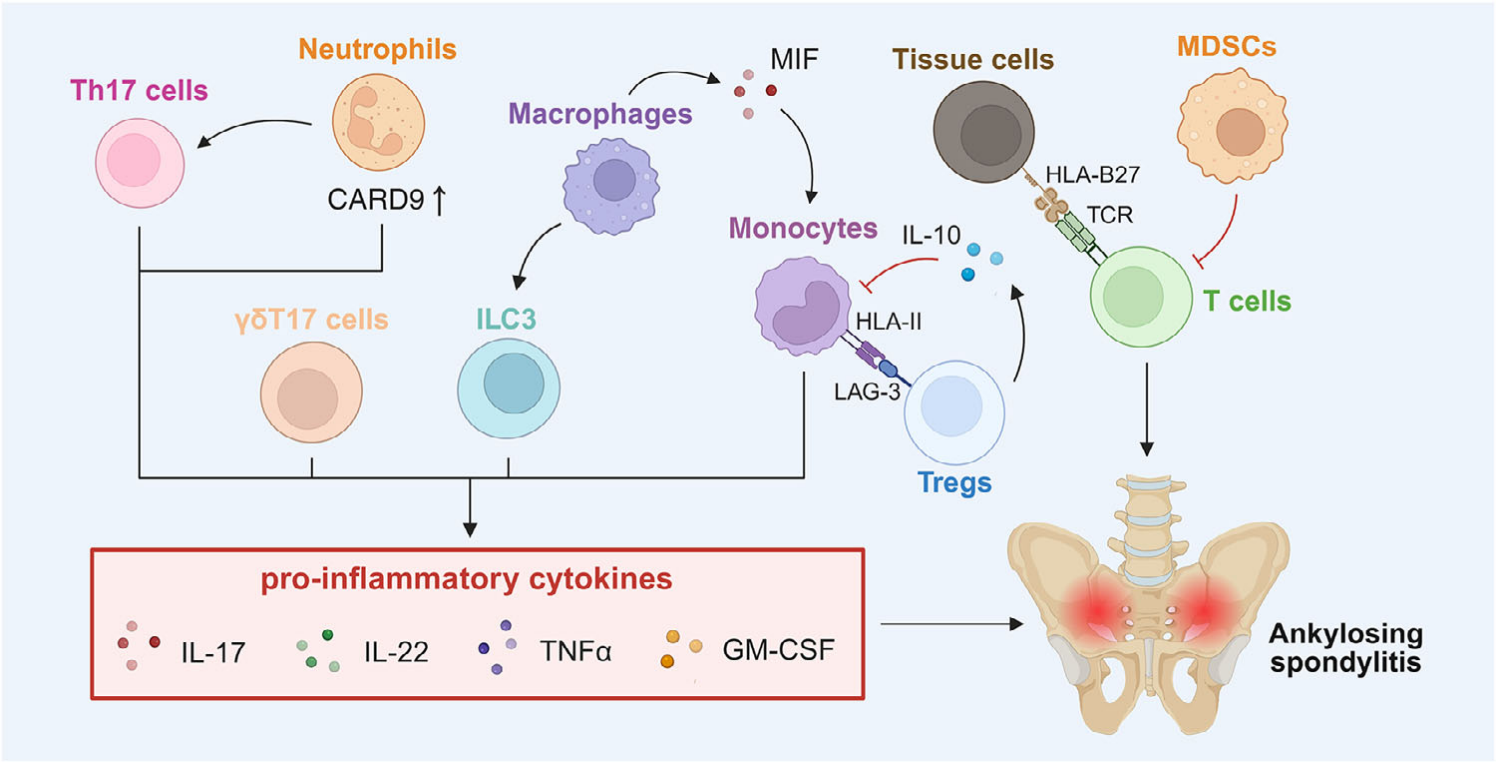
Immune‐modulatory factors in ankylosing spondylitis. IL‐17–producing Th17 cells, γδT17 cells, and ILC3s drive osteitis and enthesopathy via IL‐17, IL‐22, TNFα, and GM‐CSF. TRBV9⁺CD8⁺ T cells recognize HLA‐B27‐presented peptides, contributing to AS development. Neutrophils release preformed IL‐17 to inflamed sites, amplifying inflammation; activate through CARD9 signaling and promote Th17 polarization. CX3CR1⁺CD59⁺ macrophages activate ILC3s, inducing IL‐17 and IL‐22 production. Macrophage‐derived MIF promotes TNFα secretion from monocytes. Tregs exert immunosuppression via IL‐10 and LAG‐3, inhibiting TNFα, IL‐12, and IL‐23 production by monocytes. MDSCs suppress T cell responses via pSTAT3–arginase‐I signaling. *Abbreviations*: ILC3s, group 3 innate lymphoid cells; TNFα, tumor necrosis factor alpha; GM‐CSF, granulocyte–macrophage colony‐stimulating factor; TRBV9, T cell receptor beta variable 9; HLA‐B27, human leukocyte antigen B27; CARD9, caspase recruitment domain‐containing protein 9; MIF, macrophage migration inhibitory factor; LAG‐3, lymphocyte activation gene‐3; MDSCs, myeloid‐derived suppressor cells; pSTAT3, phosphorylated signal transducer and activator of transcription 3; arginase‐I, arginase type I.

AS is a chronic inflammatory disorder mainly affecting the axial skeleton, causing severe pain, stiffness, and eventual spine fusion [125]. The immune microenvironment has been indicated to play key a role in the pathogenesis of AS, with HLA‐B27 (human leukocyte antigen B27) and the IL‑23/IL‐17 axis being representative [126] (Figure 2).

#### 3.2.1 Immuno‐Stimulatory Factors in AS

##### 3.2.1.1 T Cells

T cells act as central mediators of inflammation in AS, orchestrating pathological immune responses through cytokine production and activation signaling. Their proinflammatory functions are primarily mediated by IL‐17‐producing subsets—including Th17 cells, γδT17 cells, and group 3 innate lymphoid cells (ILC3s)—which promote osteitis and enthesopathy through the secretion of IL‐17, IL‐22, TNFα, and granulocyte–macrophage CSF (GM‐CSF) [127]. In AS, expanded Th17 cell populations with enhanced survival capacity infiltrate joints and entheses, and their abundance positively correlates with disease severity [128, 129, 130]. Notably, the role of CD8^+^ T cells in AS remains controversial. Oligoclonal expansions of CD8^+^ T cells suggest antigen‐specific involvement [131, 132, 133]; however, experimental depletion of these cells does not prevent disease in animal models [134, 135], indicating they may not be indispensable for pathogenesis. Nevertheless, recent evidence identifies autoreactive TRBV9^+^CD8^+^ T cells that recognize HLA‐B27‐presented peptides as key contributors to AS development [136]. Selective depletion of these cells represents a novel, potentially curative immunotherapeutic approach.

##### 3.2.1.2 Neutrophils

Neutrophils contribute to AS pathogenesis through innate immune hyperactivation, promotion of Th17 polarization, and induction of tissue inflammation. In AS facet joints, neutrophils constitute the predominant IL‐17^+^ cell population, capable of storing and releasing preformed IL‐17 into inflamed tissues, thereby amplifying local inflammatory responses [125, 137, 138]. A more recent study reports that neutrophils drive AS progression through CARD9 (caspase recruitment domain‐containing protein 9)‐dependent activation and direct Th17 polarization, establishing them as key mediators of the “mixed‐pattern” inflammation that bridges both innate and adaptive immune responses. Targeting the neutrophil–CARD9–IL‐17A axis represents a promising therapeutic strategy, especially for early‐stage AS or patients carrying the CARD9^S12N^ variant [139]. However, the functional heterogeneity of neutrophil subtypes, their crosstalk with other IL‐17‐producing cells, and their potential involvement in bone formation and resorption remain poorly defined, warranting further investigation.

##### 3.2.1.3 Macrophages

Macrophages play a pivotal role in AS by bridging innate immune activation, biomechanical stress, and structural damage, thereby representing attractive therapeutic targets [125]. A distinct subset of CX3CR1^+^CD59^+^ macrophages produces IL‐23 and TL1A (TNF ligand superfamily member 15, also known as TNFSF15) in the gut and migrates to joints and bone marrow via CCR9 (chemokine C‐C motif receptor 9), where they activate ILC3s and induce IL‐17/IL‐22 production [140, 141]. Additionally, macrophage‐derived MIF (macrophage migration inhibitory factor) promotes TNF secretion, enhances osteoblast mineralization, and contributes to radiographic disease progression [142]. CD68⁺ macrophages infiltrate the entheses and sacroiliac joints, with their abundance correlating with the severity of MRI‐detected inflammation [143, 144].

#### 3.2.2 Immuno‐Suppressive Factors in AS

##### 3.2.2.1 Tregs

Tregs are central regulators of immune homeostasis and play a critical role in restraining excessive inflammation in AS. However, exosomes derived from patients with AS have been shown to inhibit Treg proliferation [145], suggesting a potential functional impairment of these cells in the disease context. A meta‐analysis reports decreased peripheral Treg proportion in AS patients [146], whereas another study identifies a positive correlation between the frequency of peripheral Tregs and disease activity, suggesting the existence of a compensatory feedback mechanism in which Tregs act as natural brakes to restrain overactive immune responses [147]. Mechanistically, Tregs mediate their immunosuppressive effects through the production of IL‐10 and the expression of LAG‐3 (lymphocyte activation gene‐3), which potently suppresses the secretion of inflammatory cytokines such as TNFα, IL‐12, and IL‐23 by monocytes [148]. Additionally, a study on spondyloarthritides (SpA), a group that includes AS as a key subtype, demonstrated that blockade of surface molecule ICOS (inducible costimulator) on Tregs enhanced their production of IL‐10 and mitigated disease progression [149]. These findings underscore the insufficient protective role of Tregs in AS and highlight their potential as therapeutic targets.

##### 3.2.2.2 MDSCs

MDSCs remain relatively understudied in the context of AS. In 2018, Liu et al. [150] reported elevated levels of both M‐MDSCs and PMN‐MDSCs in the peripheral blood of AS patients, with M‐MDSC abundance positively correlating with disease severity. Functionally, these MDSCs suppress T cell responses in vitro via the pSTAT3/arginase‐I signaling pathway [150]. Further investigation into the phenotypic characteristics, tissue distribution, and functional dynamics of MDSCs in AS—particularly within inflamed joints and entheses—is essential for elucidating their role in disease progression.

### 3.3 Psoriatic Arthritis

PsA, a subtype of SpA affecting up to 30% of patients with psoriasis, severely involves both axial and peripheral joints [151]. The synovial membrane inflammation in PsA is characterized by hypervascularization, immune cell infiltration, and excessive release of proinflammatory mediators that stimulate FLSs to invade adjacent cartilage and bone [152]. Consequently, the exploration of the immune microenvironment within the synovium is crucial to understanding PsA pathogenesis and identifying novel therapeutic targets.

#### 3.3.1 Immuno‐Stimulatory Factors in PsA

##### 3.3.1.1 DCs

DCs serve as central orchestrators of inflammation in PsA. Their aberrant activation, together with the production of key proinflammatory cytokines, particularly IL‐23, IL‐12, and TNFα, and their ability to prime and polarize pathogenic T cell subsets (Th17, Th17‐like cytotoxic T cells, and Th1), are fundamental drivers of synovio–entheseal inflammation, joint destruction, and the systemic manifestations characteristic of PsA [153, 154]. Therapeutic targeting of DC‐derived cytokines, especially IL‐23 (e.g., guselkumab, risankizumab) and IL‐12/23 axis (e.g., ustekinumab), has proven effective in directly counteracting the proinflammatory functions of DCs in PsA [155, 156, 157, 158].

##### 3.3.1.2 T Cells

T cells, comprising diverse functional subsets, are pivotal in driving inflammation in PsA. CD8^+^ T cells, enriched in the synovial fluid and tissue, exhibit clonal expansion and produce proinflammatory cytokines such as IFNγ and TNFα, directly contributing to joint destruction [152]. Among CD4^+^ T cells, the Th17 subset plays a critical role by secreting IL‐17, which activates synovial fibroblasts, promote osteoclastogenesis, and facilitate neutrophil recruitment [159, 160, 161]. Additional subsets, including Th22 and Th9 cells, further amplify inflammation through IL‐22 and IL‐9 production, respectively [159, 162]. Moreover, systemic T cell trafficking and exosome‐mediated signaling have been implicated in the dissemination and perpetuation of inflammation across tissues [163]. Notably, the emerging concept of “inflammazone” underscores compartmentalized nature of T cell‐driven inflammation in PsA and supports the rationale for zone‐specific therapeutic interventions. Inspiring examples include the use of dNP2–ctCTLA‐4 to rebalance Treg/effector T cell dynamics in the skin, and IL‐23R antagonists to limit joint involvement and disease progression [164]. These insights position T cells not merely as initiators of PsA inflammation but as spatially adaptable drivers linking skin and joint pathology, thereby making them uniquely suited for targeted, compartment‐specific immunotherapies.

#### 3.3.2 Immuno‐Suppressive Factors in PsA

**Tregs**

Although not extensively studied, Tregs remain the best characterized immunosuppressive population within the PsA microenvironment. The immunosuppressive properties of Tregs in PsA mirror those observed in AS, primarily through the expression of IL‐10 and LAG‐3, which inhibit inflammatory cytokine production [148]. However, Tregs in PsA are frequently compromised both numerically and functionally. While some studies report enhanced Treg recruitment to inflamed joints relative to peripheral blood [165], others demonstrate a reduction in Treg numbers within the synovium [166]. This contrasts with the relatively stable Treg population observed in psoriatic skin lesions [166], suggesting a site‐specific divergence in Treg suppressive capacity. Functionally, Treg impairment in PsA is marked by upregulated expression of CTLA4, TIGIT (T cell immunoreceptor with Ig and ITIM domains), PD‐1 (programmed cell death protein 1), and GITR (glucocorticoid‐induced TNFR‐related protein), accompanied by reduced TIM‐3 (T cell immunoglobulin and mucin‐domain containing‐3) expression. Additionally, disrupted Treg–OCP crosstalk via CD244/CD48 signaling further contributes to defective immune regulation [167]. Encouragingly, therapeutic interventions such as anti‐TNF [168] or IL‐23p19 [169] blockade have been shown to restore Treg function and attenuate disease progression. Of particular concern is the identification of a subset of noncanonical proinflammatory Tregs in severe PsA. These cells express IL‐17, CD161, and RORγt (retinoic acid‐related orphan receptor gamma t), exhibiting even greater osteoclastogenic activity than conventional Th17 cells [170]. Collectively, findings highlight the multifaceted and context‐dependent roles of Tregs in PsA, emphasizing the need for nuanced therapeutic approaches that preserve or reprogram protective Treg functions while restraining their pathogenic phenotypes.

### 3.4 Dermatomyositis

DM, one of the principal subtypes of idiopathic inflammatory myopathies (IIMs), is characterized by distinctive cutaneous manifestations—such as Gottron's sign—and by muscular symptoms, most notably symmetric proximal muscle weakness, typically without prominent myalgia [171]. Although the precise pathogenesis of DM remains incompletely understood, accumulating evidence implicates aberrant activation of the complement system as a central event, leading to capillary injury and subsequent ischemia, microinfarction, hypoperfusion, and perifascicular muscle atrophy [172]. Myositis‐specific antibodies (MSAs)—including anti‐Mi‐2, anti‐melanoma differentiation‐associated protein 5 (MDA5), anti‐nuclear matrix protein 2, anti‐transcription intermediary factor 1 (TIF1)—have emerged as valuable biomarkers for the diagnosis and subclassification of DM [173]. The immune microenvironment of DM is characterized by infiltration of macrophages, CD4⁺ T cells, B cells, and plasma cells within both perimysial and perivascular regions [171].

#### 3.4.1 Immuno‐Stimulatory Factors in DM

##### 3.4.1.1 Macrophages

Macrophages play a crucial role in the pathogenesis of DM [174], with studies reporting increased infiltration of both M1 and M2 phenotypes [175]. Biomarkers associated with macrophage activation—such as soluble CD163, CD206, neopterin, and galectin (Gal)‐3/9—are frequently used to evaluate disease activity [176]. Transcriptomic analyses of skin lesions further demonstrated upregulated inflammatory responses, with M1 macrophage infiltration positively correlating with the expression of CXCL10 and CXCL11 [177]. However, most of these findings are derived from bioinformatic analyses, and the precise molecular mechanisms remain unclear. A study involving 19 DM and 6 PM patients identified elevated serum levels of IFNγ and IL‐17A, and proposed a protective role for miR‐146a. Decreased miR‐146a expression promoted REG3A (regenerating islet‐derived protein 3 alpha) production and enhanced inflammatory macrophage migration [178]. Unfortunately, this study did not clearly distinguish the pathogenic mechanisms between DM and PM.

Macrophages activity has also been implicated in specific DM subtypes. For example, in anti‐MDA5^+^ DM, monocyte‐derived macrophages activated by type I interferons secrete IP‐10 (IFNγ‐induced protein 10), forming a self‐amplifying loop that promotes macrophage infiltration [179]. Elevated levels of macrophage‐related cytokines—including IL‐6, IL‐8, IL‐18, and CCL2—correlate positively with anti‐MDA5 antibody titers [180], implicating this autoantibody in macrophage inflammation. Moreover, monocyte‐derived alveolar macrophages exhibit potent proinflammatory and profibrotic functions in anti‐MDA5‐associated interstitial lung disease (ILD) [181]. Additionally, in anti‐Mi2^+^ DM, a significantly higher endomysial macrophage infiltration score has been reported, further linking macrophage accumulation to this specific MSA subtype. Taken together, current evidence supports a strong association between macrophage infiltration and DM pathogenesis and its complications. Nevertheless, the underlying cellular and molecular mechanisms remain largely unexplored and warrant further mechanistic investigation.

##### 3.4.1.2 T Cells

T cells play diverse roles across DM subtypes, exhibiting distinct functional characteristics depending on both disease context and their phenotypic subtype. Among them, an increased frequency of TIGIT⁺CD226⁺CD4⁺ T cells with enhanced effector function has been observed, particularly in patients with ILD [182]. Peripheral helper T cells are enriched in inflamed DM muscle tissue and are believed to contribute to local B cell activation and disease pathogenesis [183]. In contrast, TIF1γ‐induced experimental myositis is primarily driven by CD8⁺ T cells and type I IFN signaling, independent of CD4⁺ T cells or B cells [184]. In juvenile DM (JDM), peripheral T cells display heightened type I IFN responses and may interact with inflammatory monocytes to exacerbate tissue damage [185]. Meanwhile, CXCR5^−^ Th2 cells have been indicated to correlate with disease activity and contribute to JDM pathogenesis, serving as potential biomarkers for monitoring disease progression [186]. In anti‐MDA5^+^ DM, characterized by a reduction in peripheral T cell counts, the expression of retinoic acid‐inducible gene‐I in T cells promotes apoptosis and inhibits proliferation [187]. However, the proportions of peripheral activated CD38^+^CD4^+^ and CD38^+^CD8^+^ T cells—both positively correlated with elevated serum IFNα levels—are significantly increased, especially in patients with rapidly progressive ILD [188]. A high frequency of circulating ISG15^+^CD8^+^ T cells, defined by expression of IFN‐stimulated gene product 15, has also been associated with poor prognosis [189]. Moreover, reduced levels of both CD4⁺ and CD8⁺ T cells are indicative of increased risk for ILD development in anti‐MDA5^+^ DM [190]. Mechanistically, CD4⁺CXCR4⁺ T cells are elevated in both the peripheral blood and bronchoalveolar lavage fluid of patients with IIM‐related ILD, where they promote pulmonary fibroblast proliferation in an IL‐21‐dependent manner. However, this observation does not distinguish between DM, PM, and other IIM subtypes. In conclusion, current studies largely focus on the phenotypic characterization of T cell subsets in DM, yet the precise molecular and immunological mechanisms underlying their roles in disease pathogenesis remain to be fully elucidated.

##### 3.4.1.3 B Cells

B cells are potent antibody producers and are critically involved in the pathogenesis of DM. In DM patients, increased frequencies of CD20^+^ B cells [191] and CD38^+^ plasma cells [192] have been observed, along with elevated levels of the B cell‐activating factor BAFF [193, 194]. An elevated B cell percentage has also been proposed as a biomarker of disease activity in JDM [195]. Radke et al. [196] classified adult DM into three histopathological subtypes based on B cell infiltration patterns: classic (occasional B cells without clusters), B cell‐rich, and follicle‐like (indicative of B cell maturation and activated immunity), and established a correlation between B cell content and type I IFN‐related gene expression, thereby linking humoral immunity to IFN‐I‐driven pathogenesis. Functionally, B cells in DM display dual roles: subsets producing proinflammatory cytokines (e.g., IL‐6, IFNγ) coexist with immune‐regulatory TGFβ⁺ B cells [191]. In JDM, the B cell compartment is skewed toward an immature naïve and transitional phenotype, characterized by high CD24 and CD5 expression and reduced CD39 [197], accompanied by inflammatory Th2 polarization [198] and heightened type I IFN signatures [199]. Activation of B cells via Toll‐like receptor (TLR)7 signaling in the presence of IFNα promotes a proinflammatory skew with diminished IL‐10 production [199]. Increased otoferlin expression—primarily in unswitched B cells and plasmablasts—correlates positively with disease activity and muscle weakness, suggesting its potential as a biomarker for JDM [200]. Moreover, in anti‐MDA5^+^ DM, certain autoreactive B cell clones produce monoclonal antibodies—distinct from anti‐MDA5—that directly stimulate IFNγ secretion from peripheral blood cells via a monocyte‐dependent pathway, linking B cell‐derived autoantibodies to disease severity [201]. In addition, therapeutic strategies targeting B cells and plasma cells—such as anti‐CD19 CAR‐T cells [202, 203], belimumab (anti‐BAFF) [204], and daratumumab (anti‐CD38 plasma cells) [205]—have shown encouraging results in clinical trials, underscoring the therapeutic potential of B cell‐directed interventions in DM. Taken together, these findings highlight that B cells in DM are not merely antibody producers but exhibit diverse pathogenic and regulatory functions, representing both valuable biomarkers and promising therapeutic targets.

#### 3.4.2 Immuno‐Suppressive Factors in DM

**Tregs**

Tregs are the predominant immunosuppressive cell population within the DM microenvironment. In DM, Treg numbers are often reduced [206, 207, 208], accompanied by a skewed Th17/Treg ratio that favors Th17‐driven inflammation [209, 210]. This imbalance is further exacerbated by decreased levels of the anti‐inflammatory cytokines TGF and IL‐10 [206]. IL‐2 has been demonstrated to promote Treg expansion and help restore the Th17/Treg balance [207, 208, 211]. In JDM, Tregs are detected in approximately 30% of cases, suggesting limited but noteworthy involvement [212]. However, these cells exhibit reduced CTLA‐4 expression, indicating functional impairment [213]. In DM, Treg inactivity may result from the upregulation of acid sphingomyelinase [214]—an enzyme involved in lipid metabolism—as well as increased expression of the long noncoding RNA HAGLR (homeobox D gene cluster antisense growth‐associated long noncoding RNA), which suppresses Runt‐related transcription factor (RUNX)3 and in turn downregulates the Treg‐defining transcription factor Foxp3 [215]. However, the precise mechanisms through which Tregs mitigate DM inflammation—such as suppressing effector T cells and secreting protective cytokines— remain poorly defined. Paradoxically, a study involving both DM and PM patients (without distinguishing between the two) found that Treg numbers in inflamed muscle decreased after immunosuppressive therapy, although their relative loss may contribute to poor clinical outcomes due to impaired muscle tissue regeneration [216]. Taken together, Tregs represent a promising target for cell‐based immunomodulation in pathological DM microenvironment and merit further investigation.

### 3.5 Polymyositis

PM was historically used as an umbrella term for IIMs based on clinical presentation, until distinct subtypes such as DM and inclusion body myositis were clearly defined [217]. With advances in histopathology and immunology, entities previously classified as PM—such as antisynthetase syndrome and immune‐mediated necrotizing myopathies—have been reclassified, resulting in a lower reported incidence of true PM cases [218]. Today, PM is generally defined by specific pathological features, notably the endomysial infiltration of CD8⁺ cytotoxic T cells targeting MHC class I‐expressing muscle fibers, forming the “CD8⁺/MHC‐I complex” [219].

#### 3.5.1 Immuno‐Stimulatory Factors in PM

##### 3.5.1.1 T Cells

T cells play an active role in the pathogenesis of PM, particularly CD8^+^ T cells, which directly invade non‐necrotic muscle fibers, leading to muscle fiber death and tissue injury [220]. The formation of CD8^+^/MHC‐I complexes around muscle fibers triggers the release of perforin granules, leading to muscle lysis [219]. Transcriptomic analyses of CD4^+^ and CD8^+^ T cells from peripheral blood of DM and PM patients reveal minimal gene expression differences in CD4^+^ T cells but substantial differences in CD8^+^ T cells, mainly involving lymphocyte migration and T cell differentiation pathways [221]. These results suggest that CD8^+^ T cells, rather than CD4^+^ T cells, are key players distinguishing the immune mechanisms of PM and DM [221]. During PM, CD8^+^ T cells exhibit increased STAT and pZAP70 (phosphorylated zeta‐chain‐associated protein kinase 70) activation, which improves notably following clinical remission [222]. The recruitment of CD8^+^ T cells into inflamed tissues may be mediated by CXCR3 signaling, as evidenced by the observed reductions of CD8^+^CXCR3^+^ T cells in the peripheral blood of PM patients [223], although this requires further validation. A murine model of PM, based on C protein peptide‐induced myositis—in which autoaggressive CD8+ T cells are activated independently of CD4+ T cell help—demonstrated that blockade of the costimulatory molecules CD80/86 has therapeutic effects, underscoring the critical role of T cells in PM pathogenesis [224]. Moreover, CD28^null^ CD4^+^ and CD8^+^ T cells have been identified as potent effectors in PM, mediating perforin‐dependent cytotoxicity against muscle cells, and can be further influenced by IFNγ‐induced HLA expression on muscle cells [225]. Furthermore, PM has been reported as an immune‐related adverse events following cancer immunotherapies targeting PD‐1 and CTLA‐4, characterized by intense T cell infiltration indicative of inflammatory myopathy [226, 227]. Collectively, T cells—especially CD8^+^ subsets—play a central role in the pathological imbalance in PM, mediating direct cytotoxic effects on muscle fibers.

##### 3.5.1.2 Macrophages

Macrophages play a prominent role in the pathogenesis of PM. Studies have reported an increased proportion of CD68⁺ macrophages accompanied by elevated NLRP3 (nucleotide‐binding domain, LRR and pyrin domain‐containing protein 3) expression, which activates the NLRP3/caspase‐1/IL‐1β axis that upregulates MHC I molecules on muscle cells, making them more susceptible to T cell‐mediated attack [228]. The extent of macrophage infiltration and their polarization status are critical determinants of the immune microenvironment in PM. For example, estrogen promotes inflammatory macrophage infiltration by downregulating miR‐21, which in turn enhances CXCL10 expression [229]; IL‐15 facilitates macrophage infiltration through activation of the NF‐κB pathway and upregulation of MMP‐9 [230]; meanwhile, miR‐381 suppresses inflammation and macrophage infiltration via downregulation of HMGB1 (high mobility group box 1) [231]. Inhibition of gC1qR (globular C1q receptor) has been shown to promote M2 macrophage polarization, underscoring the complement system's involvement in macrophage‐related PM pathogenesis [232]. Additionally, CD44, expressed on macrophages of both M1 and M2 phenotypes, is observed in the PM microenvironment [233]. Serum levels of soluble CD163 have been found to be significantly elevated and correlate with disease severity in patients with PM, highlighting its potential as a valuable biomarker for disease assessment [234, 235]. Notably, this finding underscores the dual effects of this macrophage activation marker, despite its common association with M2 polarization. Furthermore, a case of JPM with UNC13D (Unc‐13 homolog D) mutation complicated by recurrent macrophage activation syndrome characterized by overwhelming macrophage activation and cytokine storm further emphasizes the critical role of macrophages in PM etiology [236]. In conclusion, these findings that interventions aimed at limiting proinflammatory macrophage infiltration and disrupting macrophage‐mediated amplification of T cell responses may hold therapeutic promise in PM management.

##### 3.5.1.3 B Cells

Although PM pathogenesis is primarily T cell‐mediated, B cell contributions, while relatively limited, cannot be entirely excluded. Importantly, studies have reported elevated BAFF concentrations in anti‐Jo‐1^+^ PM, which positively correlates with autoantibody titers [193]. Moreover, individual case reports suggest potential involvement of B cells and plasma cells. For example, in a case of chronic graft‐versus‐host disease‐related PM, muscle biopsy revealed muscle fiber destruction accompanied by concurrent infiltration of CD20⁺ B cells and T cells [237]. Another PM case presenting with markedly elevated serum IgG4 levels and IgG4⁺ plasma cell infiltration has been described, highlighting the possible role of plasma cells in PM pathogenesis [238]. However, such isolated observations are insufficient to establish a definitive association between B cell infiltration and PM pathogenesis, leaving this an underexplored area in need of further investigation.

#### 3.5.2 Immuno‐Suppressive Factors in PM

**Tregs**

Although there are only a limited number of studies addressing regulatory immune cells in the PM immune microenvironment, existing research has reported a decline of Tregs in the peripheral blood of PM patients, a deficit that low‐dose IL‐2 treatment has been shown to effectively counteract [207, 208]. The level of Tregs in muscle biopsies is also considered a marker of treatment response, where higher Treg infiltration correlates with improved clinical outcomes [239]. Tregs are implicated in myositis‐associated ILD as well. Research involving PM/DM patients (without distinguishing between the two) demonstrated a significant reduction in both the absolute count and proportion of peripheral Tregs compared with healthy controls, particularly in patients with ILD. Notably, decreased circulating Tregs have been identified as an independent risk factor for ILD onset [240]. In summary, Tregs probably exert a protective role within the PM immune microenvironment, yet remain insufficiently studied. Their functional modulation may offer promising avenues for therapeutic intervention.

## 4 Degenerative MSDs

Degenerative MSDs, such as OA, osteoporosis, intervertebral disc (IVD) degeneration (IDD), sarcopenia, and tendinopathy, are primarily characterized by the progressive deterioration of structural tissues. This process arises from an imbalance among cumulative mechanical stress, age‐related changes, and diminished repair capacity, which is often propelled by a chronic, immune‐mediated, low‐grade inflammation that disrupts normal tissue homeostasis and fostering a procatabolic environment. Understanding this immuno‐degenerative axis is key to deciphering the pathophysiology of these conditions.

### 4.1 Osteoarthritis

**FIGURE 3:**
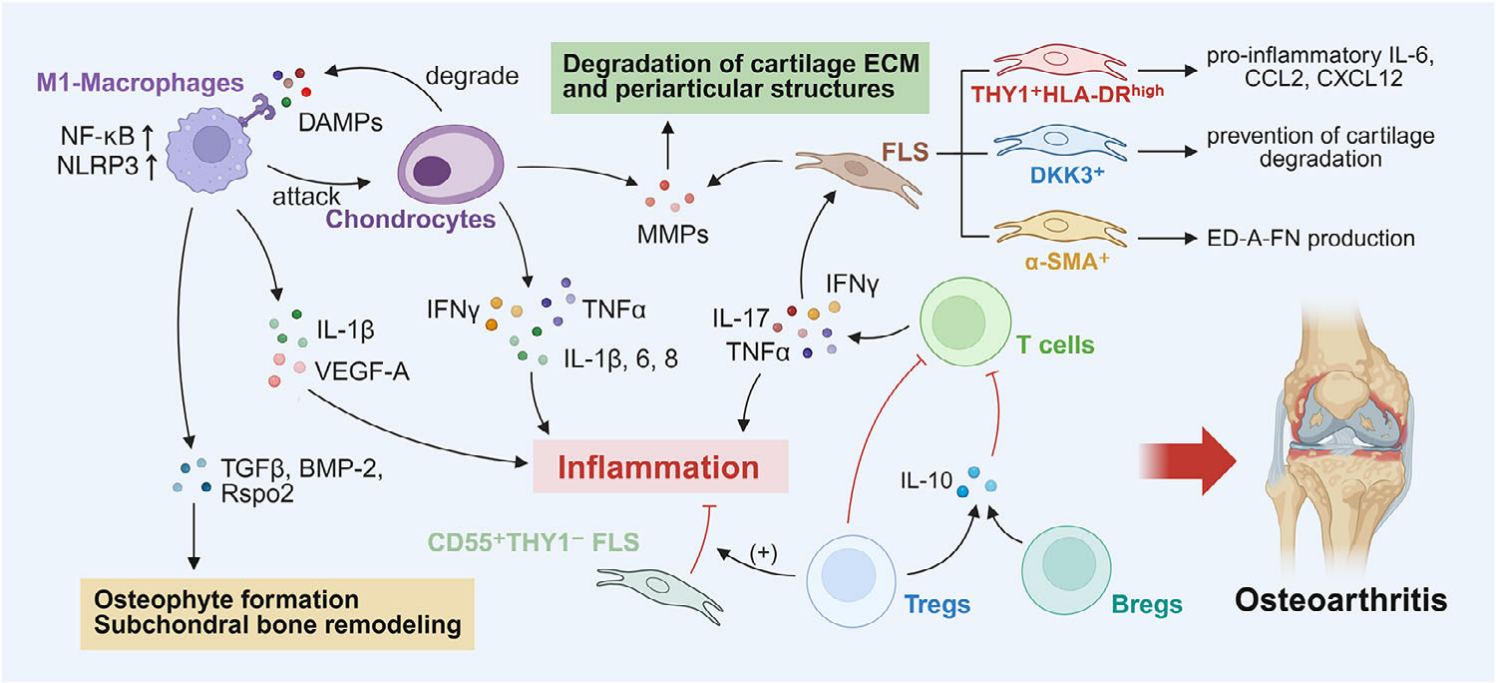
Immune‐modulatory factors in osteoarthritis. M1 macrophages attack tissue cells and are activated by ECM degradation products acting as DAMPs. These bind to TLRs and trigger NF‐κB and NLRP3 inflammasome signaling, reinforcing M1 polarization. Macrophages upregulate MMPs (e.g., MMP‐1, ‐3, ‐9, ‐13) and inflammatory cytokines (IL‐1β, TNFα, IL‐6, IL‐8, IFNγ) in chondrocytes; chondrocytes, in turn, stimulate macrophages to produce IL‐1β and VEGF‐A. M1 macrophages contribute to osteophyte formation and subchondral bone remodeling via TGFβ, BMP‐2, and Rspo2. FLS produce inflammatory cytokines (e.g., IL‐6, IL‐8, TNFα) and MMPs (e.g., MMP‐1, ‐3, ‐13), promoting cartilage degradation and synovial fibrosis. THY1⁺HLA‐DR^high^ FLS in the sublining layer exhibit proinflammatory phenotypes (e.g., IL‐6, CCL2, CXCL12), while DKK3⁺ FLS may counter cartilage breakdown. α‐SMA⁺ myofibroblast‐like FLS in both lining and sublining layers promote ED‐A‐F overproduction, driving TNFα expression in macrophages. CD55⁺THY1^−^ lining FLS mediate aberrant bone formation through BMP‐6 signaling. T cells secrete IL‐17 and TNFα, enhancing MMP production (e.g., MMP‐9) by synovial fibroblasts and resident cells, thereby accelerating ECM degradation and damage to periarticular structures. Tregs and Bregs suppress inflammation through IL‐10 secretion. *Abbreviations*: ECM, extracellular matrix; DAMPs, damage‐associated molecular patterns; TLRs, Toll‐like receptors; NF‐κB, nuclear factor kappa B; NLRP3, NOD‐, LRR‐ and pyrin domain‐containing protein 3; MMPs, matrix metalloproteinases; TNFα, tumor necrosis factor alpha; IFNγ, interferon gamma; VEGF‐A, vascular endothelial growth factor A; TGFβ, transforming growth factor beta; BMP‐2, bone morphogenetic protein 2; Rspo2, R‐spondin 2; FLS, fibroblast‐like synoviocytes; THY1, thymocyte antigen 1; HLA‐DR, human leukocyte antigen–DR isotype; DKK3, dickkopf WNT signaling pathway inhibitor 3; α‐SMA, alpha‐smooth muscle actin; ED‐A‐F, extra domain A fibronectin; BMP‐6, bone morphogenetic protein 6.

OA, a highly prevalent age‐related degenerative joint disease that commonly affects individuals aged over 65 years worldwide, is characterized by progressive impairment of synovial joints through cartilage degradation, subchondral bone remodeling, and synovitis [241, 242]. Inflammation in OA is characterized by low‐grade systemic and local inflammaging (chronic sterile inflammation associated with aging), driven by innate immune activation, macrophage and T cell infiltration, and excessive release of proinflammatory mediators [242]. These processes stimulate FLSs and chondrocytes to secrete catabolic enzymes, leading to ECM destruction, osteophyte formation, and progressive structural deterioration of the joint (Figure 3).

#### 4.1.1 Immuno‐Stimulative Factors in OA

##### 4.1.1.1 Macrophages

Macrophages, as pivotal innate immune cells within synovial tissues, play a central role in driving inflammation and structural degeneration in OA. Although macrophages exhibit dual phenotypes, OA synovium is predominantly infiltrated by M1‐polarized macrophages, which exacerbate disease progression through multiple pathological mechanisms. ECM degradation products—such as cartilage fragments and fibronectin—function as damage‐associated molecular patterns that engage pattern recognition receptors, including TLRs, on macrophages, thereby activating NF‐κB signaling and the NLRP3 inflammasomes, which further promotes M1 polarization in a feed‐forward manner [243, 244, 245, 246].

In OA, chondrocytes and activated macrophages engage in reciprocal proinflammatory crosstalk. In coculture, macrophages increase the expression of matrix‐degrading metalloproteinases (e.g., MMP‐1, MMP‐3, MMP‐9, MMP‐13) and inflammatory cytokines (IL‐1β, TNFα, IL‐6, IL‐8, IFNγ) in chondrocytes, while chondrocytes stimulate macrophages to produce more IL‐1β and VEGF‐A, indicating mutual amplification of inflammation and tissue degradation [247]. In addition to their proinflammatory effects, M1 macrophages contribute to osteophyte formation and subchondral bone remodeling through the secretion of TGFβ, BMP‐2, and R‐spondin‐2 (Rspo2) [248, 249]. While the pathogenic role of M1 macrophages is well recognized, the effects of global macrophage depletion remain controversial. Studies using clodronate liposomes suggest that complete macrophage depletion reduces MMPs and osteophyte development [250, 251]. However, others show that complete macrophage ablation, such as in Fas‐induced apoptosis transgenic mice, exacerbates synovitis and fail to preserve cartilage integrity [252]. Collectively, these findings underscore that OA severity correlates more closely with the M1/M2 macrophage ratio than with the overall macrophage count [253]. The remarkable plasticity of macrophages underscores the need for nuanced, phenotype‐specific therapies that modulate macrophage polarization, rather than indiscriminate depletion.

##### 4.1.1.2 Fibroblast‐Like Synoviocytes

FLSs are key stromal cells within the synovial membrane that maintain joint homeostasis under physiological conditions but drive pathological processes during OA [254]. FLSs undergo a phenotypic shift toward a proinflammatory and matrix‐degrading state, driving synovitis, cartilage degradation, and synovial fibrosis. OA‐associated FLSs are characterized by increased production of inflammatory cytokines (e.g., IL‐6, IL‐8, TNFα) and matrix‐degrading enzymes (e.g., MMP‐1, MMP‐3, MMP‐13), perpetuating cartilage degradation and synovial fibrosis [255, 256]. Importantly, FLSs are functionally heterogeneous, comprising distinct subsets with divergent inflammatory profiles. THY1⁺HLA‐DR^high^ FLS subsets in the sublining layer display proinflammatory properties (e.g., IL‐6, CCL2, CXCL12), while DKK3⁺ FLSs may contribute to cartilage protection [257, 258]. In addition, α‐SMA⁺ myofibroblast‐like FLSs in both lining and sublining layers contribute to ED‐A‐FN (extra domain A fibronectin) overproduction, which may drive TNFα production in macrophages [259]. Moreover, metabolic reprogramming of FLSs, including upregulated glycolysis (Warburg effect) and mitochondrial dysfunction, enhances ROS production and sustains chronic inflammation, further promoting fibroblast‐to‐myofibroblast transition [260, 261]. Interestingly, while some FLS subsets (e.g., CD55⁺THY1^−^ lining cells) retain lubricative roles, their expansion in OA may paradoxically promote aberrant bone formation via BMP‐6 signaling [258, 262]. Moreover, persistent activation of the TGFβ/Smad signaling pathway has been shown to drive irreversible myofibroblast differentiation [263]. In summary, the heterogeneity and functional plasticity of FLSs represent a significant challenge for developing targeted therapies in OA, necessitating subset‐specific and context‐dependent intervention strategies.

##### 4.1.1.3 T Cells

T cells play a pivotal role in driving inflammation and tissue degeneration in OA through cytokine production, activation of catabolic pathways, and orchestration of localized immune responses within the joint. Among infiltrating lymphocytes, CD4^+^ Th cells constitute the predominant T cell population in the synovium during early stages of OA. Within the OA joint, T cells secrete potent proinflammatory cytokines, such as IL‐17 and TNF [264, 265], which stimulate synovial fibroblasts and other resident cells to produce matrix‐degrading enzymes (e.g., MMP‐9) [266, 267]. These enzymes degrade the cartilage ECM and also affect periarticular structures, such as the infrapatellar fat pad, promoting the release of proinflammatory adipokines (e.g., leptin, resistin), which further accelerate cartilage catabolism and exacerbate synovitis [268, 269]. In addition to CD4^+^ T cells, CD8^+^ T cell subtypes, including IFNγ^+^ Tc1 and IL‐17A^+^ Tc17 subtypes, are significantly enriched in OA synovial fluid compared with peripheral blood, indicating local activation and effector differentiation of T cells within the inflamed joint [270]. Notably, the presence of synovial T–B cell aggregates surrounding plasma cells in late‐stage OA, along with observed alterations in circulating memory T cell populations, suggests a potential autoimmune‐like component in OA pathogenesis involving T cell activation [271]. In conclusion, T cells play key roles in OA pathogenesis by driving inflammation and tissue damage, highlighting their potential as therapeutic targets.

#### 4.1.2 Immuno‐Suppressive Factors in OA

##### 4.1.2.1 Tregs

Tregs, particularly the CD4⁺CD25⁺Foxp3⁺ subset, play a crucial role in modulating immune responses in OA. Mechanistically, Tregs exert anti‐inflammatory effects primarily through the secretion of IL‐10, which suppress inflammatory and autoimmune responses, thereby facilitating immunosuppression and symptom alleviation in OA [272]. An in vitro study further demonstrated that chondrocytes and synoviocytes cocultured with Tregs exhibit increased expression of tissue inhibitor of metalloproteinases 1, a protective molecule in OA [273].

Investigations into the OA microenvironment have revealed elevated CD4^+^CD25^+/high^CD127^low/−^ Treg frequencies in the synovial membrane during early‐stage knee OA, suggesting a compensatory immune mechanism in response to local proinflammatory conditions [274]. A large cohort study in European hip OA patients identified a protective association between higher CD4⁺CD25⁺ Treg frequencies, enhanced CD25 expression in the CD39⁺ Treg subset, and reduced OA risk [275]. In addition, lower Treg proportions, especially in the synovial membrane, are associated with greater pain and functional impairment, indicating that intra‐articular Treg infiltration may play an important role in OA pathogenesis and symptom modulation [276]. Moreover, OA patients often exhibit higher Treg levels in affected joints compared with peripheral blood [276], reflecting preferential recruitment of Tregs into the OA microenvironment. However, this increased recruitment appears insufficient to restore immune balance, since Tregs from OA patients often show reduced IL‐10 and Tim‐3 expression [277]. Consistently, a study found that higher resting Treg counts were causally associated with an increased risk of spine OA, suggesting insufficient Treg activation [278].

Treg levels, similar to those in other immune‐mediated bone diseases, serve as indicators of treatment response in OA. For example, in a randomized controlled trial involving 30 patients, those receiving Sinacurcumin (80 mg daily) for 3 months showed a significant increase in Tregs and an improved Treg/Th17 balance. These immunological changes, along with improvements in OA visual analog scores and inflammatory biomarkers (e.g., CRP), support the therapeutic efficacy of this intervention [279].

##### 4.1.2.2 Bregs

As key players in immune modulation, Bregs remain relatively understudied in the context of OA. Their ability to produce immunosuppressive cytokines, particularly IL‐10, is a defining functional feature. Studies have shown that higher synovial abundance of Bregs is inversely correlated with OA severity, suggesting a protective role. Interestingly, phenotypical analysis of IL‐10‐producing Bregs in OA reveals predominant expression of IgM and CD27, rather than conventional Breg markers such as CD24^high^ or CD38^high^, indicating a potentially unique Breg phenotype in the OA microenvironment [280]. However, due to the limited number of related studies, the precise role of Bregs in OA remains poorly defined and warrants further investigation.

### 4.2 Osteoporosis

**FIGURE 4:**
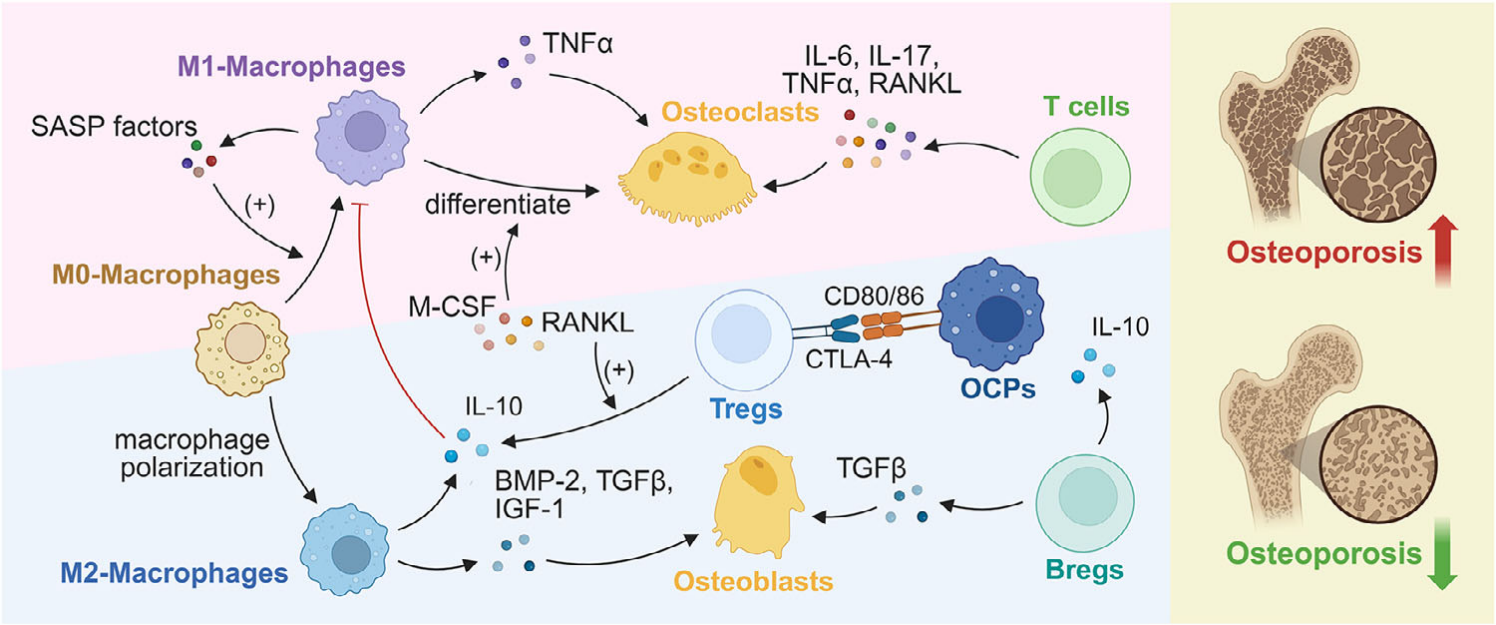
Skewed immune scenario in osteoporosis. Macrophages differentiate into osteoclasts under stimulation by M‐CSF and RANKL. TNFα secreted by macrophages amplifies RANK/RANKL signaling to promote osteoclastogenesis while concurrently suppressing osteoblast differentiation via downregulation of IGF‐1 and RUNX2. Aging leads to the accumulation of senescent macrophages that release SASP factors, driving M1 polarization and paracrine senescence, forming a self‐perpetuating loop contributing to senile osteoporosis. M2 macrophages promote osteoblast differentiation via secretion of BMP‐2, TGFβ, and IGF‐1, and exert anti‐inflammatory effects through IL‐10. Activated T cells, especially Th17 cells, secrete RANKL, TNFα, IL‐6, and IL‐17, directly enhancing osteoclast differentiation and activity. CTLA‐4 expressed on Tregs binds to OCPs and inhibits their differentiation in a dose‐dependent manner. Treg‐derived IL‐10 mediates suppression of T cell proliferation and contributes to bone‐protective immune regulation. Treg migration and the production of IL‐10 and TGFβ are dependent on RANKL signaling. Bregs suppress osteoporosis via IL‐10 secretion and modulate osteoblast activity through TGFβ. *Abbreviations*: M‐CSF, macrophage colony‐stimulating factor; RANKL, receptor activator of nuclear factor κB ligand; TNFα, tumor necrosis factor alpha; IGF‐1, insulin‐like growth factor 1; RUNX2, runt‐related transcription factor 2; SASP, senescence‐associated secretory phenotype; BMP‐2, bone morphogenetic protein 2; TGFβ, transforming growth factor beta; CTLA‐4, cytotoxic T‐lymphocyte‐associated protein 4; OCPs, osteoclast precursors.

Osteoporosis is an age‐related degenerative bone disease characterized by reduced bone mass, microarchitectural deterioration, and increased skeletal fragility, predominantly affecting postmenopausal women [281]. As the global population continues to age, the incidence of osteoporosis is steadily rising [282], underscoring its significant social and economic burden. Numerous studies have highlighted immune dysregulation within the bone microenvironment, with particular emphasis on the imbalance of Tregs and Th17 cells [283]. Further investigation in this field holds great potential for uncovering novel strategies for the prevention and treatment of osteoporosis (Figure 4).

#### 4.2.1 Immuno‐Stimulative Factors in Osteoporosis

##### 4.2.1.1 Macrophages

Macrophages play a dual role in osteoporosis but predominantly contribute to proinflammatory and bone‐resorptive processes [284]. Their activation and cytokine production are key drivers of bone loss. As members of the monocyte–macrophage lineage, macrophages can differentiate into osteoclasts under the stimulation of specific cytokines such as M‐CSF and RANKL [284]. In addition, macrophages are a major source of TNFα, which promotes osteoclastogenesis by amplifying RANK/RANKL signaling, while concurrently inhibiting osteoblast differentiation through suppression of key osteogenic factors such as IGF‐1 (insulin‐like growth factor‐1) and RUNX2 [285]. Under conditions of iron overload, intracellular accumulation of metal ions has been observed in macrophages. This state is often accompanied by elevated serum levels of proinflammatory cytokines TNFα and IL‐6, suggesting a link between iron dysregulation, systemic inflammation, and oxidative stress‐associated bone loss [286]. Moreover, aging leads to the accumulation of senescent macrophages within the bone microenvironment. These cells acquire a senescence‐associated secretory phenotype (SASP) that promotes M1 polarization and induces senescence in neighboring macrophages, thereby establishing a self‐amplifying cycle contributing to age‐related osteoporosis [287]. Despite the predominance of M1 macrophages in osteoporotic conditions, recent studies have highlighted the role of M2‐polarized macrophages in counteracting bone loss. For example, M2 macrophages facilitate osteoblast differentiation by secreting BMP‐2, TGFβ, IGF‐1, and the anti‐inflammatory cytokine IL‐10 [285, 288]. However, the specific signaling pathways and temporal dynamics regulating macrophage polarization and function in osteoporosis—particularly across distinct disease stages and etiological subtypes—remain poorly defined, highlighting the need for further in‐depth mechanistic and in vivo investigations.

##### 4.2.1.2 T Cells

T cells play a complex yet critical proinflammatory and bone‐resorptive role in the development of osteoporosis, primarily through cytokine production and interactions with OCPs. Activated T cells, particularly Th17 cells, secrete proinflammatory cytokines such as RANKL, TNFα, IL‐6, and IL‐17, which directly promote osteoclast differentiation and activation. Among them, RANKL serves as the central regulator of osteoclastogenesis, establishing a direct mechanistic link between T cell‐mediated inflammation and bone resorption. Interestingly, the gut microbiome also influences T cell‐driven bone loss. For example, segmented filamentous bacteria induce the expansion of intestinal TNF⁺ T cells and Th17 cells, which mediate bone loss under continuous parathyroid hormone (cPTH) exposure [289, 290]. In contrast, germ‐free or antibiotic‐treated mice—lacking typical T cell populations—are protected from cPTH‐induced bone loss, highlighting the interplay between gut immunity and skeletal health [290]. However, the precise molecular mechanisms linking the gut–immune–bone axis in both physiological and pathological contexts remain poorly defined and require further elucidation.

#### 4.2.2 Immuno‐Suppressive Factors in Osteoporosis

##### 4.2.2.1 Tregs

Tregs play a protective role in osteoporosis by maintaining immune tolerance and suppressing excessive osteoclast activity. The imbalance between Tregs and Th17 cells is a key contributor to osteoclast overstimulation in osteoporosis [291]. Reduced levels of Tregs are frequently observed in patients with osteoporosis [292], and impaired Treg‐mediated immune tolerance has been shown to exacerbate postmenopausal osteoporosis in ovariectomized (OVX) mouse models [32, 293]. Notably, the frequency of CD4^+^CD25^+^CD127^−/low^ Tregs has been identified as a reliable predictor for the development of osteoporosis [292]. Tregs exert their protective effects through both direct cell‐to‐cell contact and the secretion of immunomodulatory cytokines. One key mechanism involves the expression of CTLA‐4 on Tregs, which binds to OCPs and inhibits their differentiation in a dose‐dependent manner; conversely, blockade of CTLA‐4 reverses this inhibition [294]. Among secreted cytokines, IL‐10 is critical for Treg‐mediated immune regulation, acting as a key suppressor of T cell proliferation [295]. Interestingly, RANKL itself has been shown to exert dual roles in bone metabolism. Treg‐specific inhibition of RANKL has been found to impair Treg migration and reduce the production of key cytokines such as IL‐10 and TGFβ, highlighting the context‐dependent functions of RANKL within Tregs [296]. Tregs are also emerging as a promising target for novel therapeutic strategies in osteoporosis. Interventions such as oral administration of probiotics [297, 298], supplementation with gut bacterial metabolites [32], or injection of substance P [299] have been reported to enhance Treg activity and consequently alleviate bone loss in OVX mice. In conclusion, Tregs represent a critical regulatory population that counterbalances osteoclast activation in osteoporosis. Therapeutic strategies aimed at enhancing Treg function may offer novel avenues for both preventing and treating osteoporosis by restoring immune homeostasis and reducing bone resorption.

##### 4.2.2.2 Bregs

In addition to Tregs, Bregs have emerged as important immunomodulators in the pathogenesis of osteoporosis. In murine models, OVX mice exhibit a marked reduction in both Breg populations and IL‐10 levels, highlighting a strong association between Breg deficiency and postmenopausal osteoporosis [300]. Bregs suppress the progression of osteoporosis primarily through the secretion of the immunomodulatory cytokine IL‐10. Additionally, Breg‐derived TGFβ has also been shown to modulate osteogenic differentiation [301], further supporting their role in maintaining bone integrity. In vitro studies conducted under estrogen‐deficient conditions further emphasize the link between hormonal dysregulation and the immunopathogenesis of osteoporosis [302]. Notably, both adoptive transfer of Bregs [303] and in vivo induction of Bregs [304] have demonstrated therapeutic potential in mitigating bone loss in OVX models, underscoring their promise as immunotherapeutic targets for osteoporosis.

### 4.3 IVD Degeneration

IDD is among the most common degenerative MSDs, representing the leading cause of chronic low back pain and imposing substantial socioeconomic burdens. The IVD consists of the nucleus pulposus (NP), annulus fibrosus (AF), and cartilage endplate, which are indispensable for spinal mobility and mechanical load bearing [305]. Current mainstream perspectives hold that the pathological shift in ECM metabolism—favoring catabolism over anabolism—primarily driven by metabolic dysregulation of NP cells, is the major contributor to disc degeneration [306]. Due to the inherently avascular nature of the disc, the immune system's role has long been overlooked; however, accumulating evidence highlights its critical involvement in the pathogenesis of IDD.

#### 4.3.1 Immuno‐Stimulatory Factors in IDD

##### 4.3.1.1 M1 Macrophages

While macrophages are indispensable for the elimination of apoptotic NP cells through efferocytosis, M1‐polarized macrophages are key contributors to the inflammatory microenvironment within the IVD, thereby driving degeneration. Degenerated NPs, particularly at advanced stages, exhibit increased macrophage infiltration with a marked bias toward M1 polarization [307]. In healthy IVDs, the avascular architecture and the so‐called blood–disc barrier maintain an immune‐privileged environment [308]. By contrast, degenerative conditions are characterized by prominent macrophage infiltration, which is largely attributable to postinjury neovascularization and elevated chemokine production. The polarization of M1 macrophages is mediated by multiple factors, including proinflammatory cytokines such as IL‐1β and IL‐6 secreted by inflamed IVD cells with excessive NF‐κB activation [309], exosomal signaling such as miR‐27a‐3p enriched in exosomes from degenerative NP cells [310], and oxidative stress associated with elevated ROS levels [311]. Once activated, infiltrating M1 macrophages—originating from either peripheral blood or adjacent tissues—release a broad spectrum of proinflammatory cytokines and mediators, including TNFα, IL‐1β, IL‐6, and IL‐18, which stimulate resident IVD cells to produce additional inflammatory factors, thereby establishing a self‐amplifying cascade that exacerbates local inflammation [312].

In addition to their inflammatory role, M1 macrophages also contribute directly to ECM catabolism. MMPs are the major mediators of macrophage‐related matrix degradation. For instance, coculture with M1 macrophages enhances MMP13 expression while downregulating ECM‐anabolic genes such as aggrecan and collagen IIα1 in NP cells [313]. Conversely, overexpression of LGR6 (LRR‐containing G‐protein coupled receptor 6) in macrophages reduces MMP13 expression, indicating a potential protective effect against disc degeneration [314]. In addition, cytokines derived from M1 macrophages—including IL‐1β, TNFα, and IL‐6—further drive ECM degradation, although their precise cellular sources in vivo remain to be fully clarified [312]. More recently, secreted phosphoprotein 1 (SPP1) has been identified as a pivotal mediator that exacerbates degeneration through the protein kinase RNA‐like endoplasmic reticulum kinase/activating transcription factor 4/IL‐10 signaling axis [315]. Taken together, M1‐polarized macrophages, typically regarded as proinflammatory, are crucial contributors to the progression of IDD, driving both persistent inflammation and ECM breakdown. Therapeutic strategies aimed at modulating their polarization status, while harnessing their beneficial functions such as efferocytosis, could offer novel avenues for the management of this age‐related disorder.

##### 4.3.1.2 T Cells

Despite being the predominant effector cells orchestrating immune cytotoxicity, T cells have received relatively little attention in IDD research. The identification of specific T cell subtypes is particularly important. Bioinformatic analyses have revealed an increased proportion of CD8⁺ T cells in IDD, whereas CD4⁺ memory T cells and Tfh cells appear much less abundant [316]. Interestingly, γδT cells, a subset with potential anti‐inflammatory functions in IDD, are more prevalent in female mice and may underline gender‐specific differences that favor females in IDD progression [317]. Although mechanistic studies on T cell function in IDD remain limited, evidence from endplate inflammation—a key pathological process of IDD—suggests that the transcription factors TBX21 (encoding T‐bet, a Th1 lineage marker) and RORC2 (encoding RORγt, a Th17 lineage marker) in activated CD4⁺ T cells are critical contributors. Notably, these transcriptional programs can be suppressed by CD24^high^CD38^high^ Bregs from healthy donors, in an IL‐10‐ and PD‐L1‐dependent manner, highlighting a potential immunoregulatory intervention strategy for IDD [318]. In summary, investigations into the roles of T cells in IDD remain at an early stage, underscoring a largely uncharted yet highly promising frontier for future exploration.

##### 4.3.1.3 Neutrophils

Neutrophils, as rapid responders of the innate immune system, serve as indicators of systemic inflammatory status. In IDD, a high neutrophil‐to‐lymphocyte ratio (NLR) correlates positively with severe disc degeneration, serving as a potential biomarker for disease severity [319]. Recent studies have highlighted the heterogeneity of neutrophils in IDD, identifying an MIF⁺ neutrophil subpopulation functionally enriched in ECMO. These ECMO‐neutrophils secrete MIF, which interacts with atypical chemokine receptor 3 on NP cells, promoting matrix degradation and disc degeneration [320]. Bioinformatic analyses have also revealed elevated neutrophil infiltration [321] and enhanced NETs formation [322] in degenerated discs. Moreover, PRTN3, encoding proteinase 3—a neutrophil serine protease—is consistently upregulated and correlates with neutrophil activity, serving as a shared biomarker for both IDD and diabetes mellitus, suggesting a role for neutrophil‐mediated inflammation in the comorbidity of these diseases [321]. In addition, a subset of NP cells coexpressing high levels of SPP1 and intercellular adhesion molecule 1 appears to interact with neutrophils via the ANXA1–FPR1 (annexin A1–formyl peptide receptor 1) signaling pathway, potentially limiting their own inflammatory response [323]. These findings suggest that neutrophils may also exert anti‐inflammatory and immunoregulatory functions within the IDD immune microenvironment, helping to restrain excessive NP cell inflammation. However, these findings are largely based on bioinformatic analyses without experimental validation, which limits their interpretability and reliability. Nevertheless, the significant involvement of neutrophils in IDD appears indisputable.

#### 4.3.2 Immuno‐Suppressive Factors in IDD

##### 4.3.2.1 M2 Macrophages

The polarization state of macrophages critically shapes the pathogenesis of IDD, with M2 macrophages generally exerting protective and anti‐inflammatory effects. In the outer AF and endplate, infiltrating bone marrow‐derived macrophages predominantly exhibit an M2 phenotype, characterized by elevated IL‐4 and reduced IL‐1β expression, suggesting a regulatory and protective function in these regions [324]. Both in vivo and in vitro studies have demonstrated that M2 polarization alleviates IDD by inhibiting Rspo2 production, reducing NP cell apoptosis and suppressing ECM catabolism [325]. M2 macrophages also maintain the ECM phenotype of NP cells via the OPN–CD44 axis, which regulates pSmad2/3 nuclear translocation and thereby preserves IVD homeostasis [326]. In addition, coculture with M2‐conditioned media promotes NP cell proliferation and ECM synthesis, even under inflammatory conditions induced by TNFα [327]. M2‐derived exosomes also contribute to disc protection by delivering specific microRNAs, such as miR‐124‐3p, which suppresses cartilage intermediate layer protein and enhances TGFβ/Smad3 signaling [328], and miR‐221‐3p, which inhibits NP cell pyroptosis [329]. Notably, reciprocal crosstalk between NP cells and M2 macrophages have been observed, with NP cells capable of inducing M2 polarization [326]. However, as previously discussed, a proinflammatory feed‐forward loop also exists between M1 macrophages and inflamed IVD cells, making the regulatory landscape more complex and warranting further investigation. Despite their protective roles, M2 macrophages may also contribute to pathological changes in IDD. For example, IL‐10 secreted by M2 macrophages activates the Janus kinase (JAK)2/STAT3 pathway in degenerated NP cells, promoting aberrant microangiogenesis through upregulation of the VEGF/VEGFR axis [330]. Nevertheless, therapeutic strategies aimed at promoting M2 polarization are currently an active area of research. Examples include platelet‐rich plasma‐derived exosomes, which enhance M2 polarization via STAT6 phosphorylation [331], and knockdown of FCGR2A (Fc gamma receptor IIa), which suppresses M1 polarization and NF‐κB signaling while promoting M2 polarization and STAT3 activation [332]. In summary, precise modulation of macrophage polarization—especially enhancement of the M2 phenotype—holds promise as a novel therapeutic strategy for IDD.

##### 4.3.2.2 MDSCs

MDSCs have only recently been identified as novel immune participants in IDD. These immunosuppressive cells are predominantly observed during the early stages of degeneration, suggesting that they act as immune‐modulatory participants in the initial phases of disease. For example, G‐MDSCs characterized by CD11b and oxidized low‐density lipoprotein receptor 1 expression have been detected in mildly, but not severely, degenerated NP tissues. In vitro studies further demonstrated that G‐MDSCs can attenuate ECM degradation by modulating NP cell activity [333]. Another study revealed a dynamic shift in the immune landscape of IDD, from a dominance of LCN2 (lipocalin‐2)^high^ MDSCs during early degeneration to the prevalence of inflammatory IL‐1β^+^ macrophages in advanced disease [334]. Moreover, in patients with lumbar disc herniation, the frequency of circulating G‐MDSCs increased with degeneration severity, particularly in clinical stages III and IV [335]. Despite limited current research, emerging evidence positions MDSCs as pivotal early‐stage immune modulators in IDD, highlighting their potential as therapeutic targets for halting disease progression.

### 4.4 Sarcopenia

Sarcopenia is a progressive and generalized degenerative disorder of skeletal muscle that primarily, though not exclusively, affects the elderly population. Risk factors include genetic predisposition, sedentary behavior, nutritional deficiencies, and coexisting skeletal disorders [336]. Clinically, sarcopenia manifests as reduced muscle mass, diminished muscle strength, and impaired physical performance. Current research has identified myofiber alterations, insufficient blood perfusion, neuronal degeneration, ECM degradation, and tissue inflammation as key drivers of sarcopenia pathophysiology [337]. Notably, instead of acute or excessive immune activation, sarcopenia is primarily driven by a chronic low‐grade inflammatory milieu associated with immune aging. This underscores the critical role of the immune system in the progression of this aging‐related condition [338].

#### 4.4.1 Immuno‐Stimulatory Factors in Sarcopenia

##### 4.4.1.1 T Cells

T cells are important regulators of normal muscle metabolism. In adult muscle, scattered sarcolemmal MHC‐I and MHC‐II expression is accompanied by infiltration of CD8^+^ and CD4^+^ T cells, which contribute to the clearance of damaged myofibers and promote myoblast proliferation [338]. Studies have shown that CD3^+^ T cells, including both CD4^+^ and CD8^+^ subsets, progressively decline with advancing age, underscoring age‐related T cell impairment in sarcopenia [339]. In tumor‐associated sarcopenia, such as in pancreatic ductal adenocarcinoma and hepatocellular carcinoma, tumor‐infiltrating CD8^+^ T cells are strongly associated with sarcopenia and poor clinical outcomes [340, 341]. This association may, at least in part, be attributable to reduced levels of key myokines that exert important immunomodulatory effects, though this mechanism requires further validation. Additionally, exercise combined with nutritional supplementation improves muscle strength in sarcopenia, partly by modulating T cell‐related inflammatory gene expression [342]. Moreover, immunosenescent T cell phenotypes, such as CD4^+^CD28^null^ T cells, exhibit a negative correlation with skeletal muscle mass index in patients with sarcopenia [343]. Nonetheless, in very old adults (≥85 years), a senescent‐like T‐cell phenotype has been identified but shows no association with muscle strength decline or incident sarcopenia, suggesting that the precise contribution of T cells to age‐related sarcopenia remains to be fully clarified [344]. Taken together, T cells are pivotal regulators of muscle maintenance, and both their phenotypic alterations and tissue‐infiltrating status appear to influence the development and progression of sarcopenia.

##### 4.4.1.2 Macrophages

Macrophages constitute one of the most abundant immune cell populations within the muscle microenvironment. Shen et al. [345] analyzed skeletal muscle transcriptomes and identified LYVE1^+^ (lymphatic vessel endothelial hyaluronan receptor 1) resident macrophages, revealing pronounced macrophage‐enriched inflammation in sarcopenic muscle. Flow cytometry analysis demonstrated an increased proportion of M1 macrophages and a concomitant decrease in M2 macrophages in aged mice [346]. Administration of gamma‐aminobutyric acid effectively suppressed M1 macrophage activation in both the gastrocnemius muscle and spleen, reduced proinflammatory cytokines TNFα and IL‐6, and consequently alleviated sarcopenia [347]. M1 macrophages also mediate hyperphosphatemia‐induced sarcopenia by producing TNFα, which impairs myogenic differentiation, promotes myoblast senescence, and suppresses IL‐15 expression—a myokine critical for muscle regeneration and metabolic function—linking macrophage‐driven inflammation to age‐related muscle loss [348]. Interestingly, other studies have reported that increased levels of IL‐12, a cytokine characteristic of M1 macrophages, are associated with reduced sarcopenia risk [349]. A plausible explanation is that M1 macrophages exhibit context‐dependent dual roles: acutely, they clear damaged fibers and release signals that recruit satellite cells for repair, but under chronic stress or aging conditions, persistent M1 activation disrupts myogenic differentiation, promotes cellular senescence, and remodels the muscle microenvironment, thereby contributing to sarcopenia progression. This hypothesis, however, warrants further experimental validation.

Therapeutic interventions targeting macrophages have been explored for sarcopenia. For example, the nano‐adjuvant MACL@UA enhances metabolic crosstalk between macrophages and myosatellite cells and promotes macrophage‐derived glutamine nourishment [350]. Localized intramuscular treatment with calcium silicate hydrogel inhibits M1 polarization via NF‐κB deacetylation and promotes M2 polarization through STAT3 deacetylation, collectively reducing inflammatory (TNFα, IL‐6) and fibrotic (IL‐10, TGFβ) factors [351]. Overall, modulation of M1 macrophages represents a promising strategy for managing sarcopenia.

##### 4.4.1.3 Neutrophils

Neutrophils have long been recognized as active players in sarcopenia. For instance, in patients undergoing robotic gastric cancer surgery, circulating neutrophil counts were elevated in those with sarcopenia [352]. Rather than absolute neutrophil counts, the NLR is more widely adopted as a clinical indicator of poor prognosis in sarcopenia [353, 354]. Immunosenescence is associated with phenotypic alterations and functional impairment of neutrophils, with increased infiltration possibly representing a compensatory response [338]. Despite the broad clinical application of NLR in prognosis prediction, the mechanisms by which neutrophils contribute to sarcopenia remain underexplored. Evidence suggests that neutrophils in sarcopenia display increased spontaneous NET formation, thereby fueling chronic inflammation, but show impaired ROS production and diminished NET responses to phorbol 12‐myristate 13‐acetate stimulation, while their phagocytic capacity is largely preserved [355]. Furthermore, bioinformatic analyses have identified three NETosis‐ and chemokine signaling‐related genes (CXCR1, CXCR2, and LPL) as robust diagnostic markers for sarcopenia, providing further insight into the role of neutrophils [356]. Nevertheless, deeper mechanistic investigations are required to clarify these associations.

#### 4.4.2 Immuno‐Suppressive Factors in Sarcopenia

##### 4.4.2.1 M2 Macrophages

In sarcopenia, M2 macrophages show increased infiltration [357] and exert dual, context‐dependent effects on disease pathogenesis. On the one hand, their immunosuppressive properties limit excessive immune activation and facilitate muscle regeneration. For instance, IL‐25 ameliorates sarcopenia by promoting M2 macrophage polarization, which enhances satellite cell function and muscle regeneration through the sonic hedgehog/AKT (protein kinase B, PKB)/mTOR (mechanistic target of rapamycin) signaling pathway [358]. Similarly, sarcosine—downregulated in sarcopenia—activates the GCN2 (general control nonderepressible 2) signaling pathway to promote anti‐inflammatory macrophage polarization, thereby promoting adipose thermogenesis and muscle regeneration [359]. Moreover, *Magnoliae cortex*, a medicinal herb, has been reported to alleviate cisplatin‐induced sarcopenia by increasing M2 macrophage abundance [360]. On the other hand, premature or sustained activation of anti‐inflammatory cytokines within damaged tissues can impair muscle repair, and promote excessive adipose and fibrotic tissue deposition—hallmarks of aging muscles [338]. Intramuscular M2‐biased macrophages contribute to age‐related muscle fibrosis and sarcopenia, whereas their reduction, achieved by myeloid‐specific mutation of Spi1 (a transcription factor essential for myeloid development), prevents these deleterious effects [361]. Elevated levels of TGFβ, associated with M2 polarization, have also been linked to enhanced sarcopenic features [349]. Taken together, these findings suggest that therapeutic modulation of macrophage polarization should take into account not only the balance between pro‐ and anti‐inflammatory states, but also the hierarchical and time‐dependent nature of this transition to optimize muscle repair and mitigate sarcopenia progression.

##### 4.4.2.2 Tregs

CD4⁺Foxp3⁺ Tregs play a critical role in maintaining skeletal muscle integrity and modulating muscle regeneration, with their dysfunction tightly linked to disrupted muscle homeostasis. In injured muscle, Tregs accumulate in response to signals such as IL‐33 and promote muscle growth by releasing growth factors like amphiregulin [362]. Their activity depends on IL‐6/IL6Rα signaling, which is essential for Treg maturation and the maintenance of satellite cell function [362]. In pathological contexts, such as hypertension‐related sarcopenia, restoration of Treg function via interventions like Ecklonia cava extract or dieckol improves the Th17/Treg balance, reduces inflammation, and attenuates muscle atrophy [363]. However, Tregs can also have detrimental effects: overstimulation of satellite cells by Tregs may lead to sustained proliferation with delayed differentiation, potentially impairing muscle regeneration [364]. In cancer patients, overactivated Tregs are linked to impaired antitumor immunity, with Treg frequency negatively correlating with lean mass index [365]. Moreover, tumor‐associated myosteatosis is associated with increased CTLA‐4 expression in Tregs [366], suggesting that excessive or dysregulated Treg activity may contribute to muscle loss in certain disease settings. Overall, Tregs exhibit a dual, context‐dependent influence on skeletal muscle, providing protection and promoting regeneration under physiological or inflammatory conditions, but potentially contributing to muscle wasting when dysregulated or in tumor‐associated environments.

### 4.5 Tendinopathy

**FIGURE 5:**
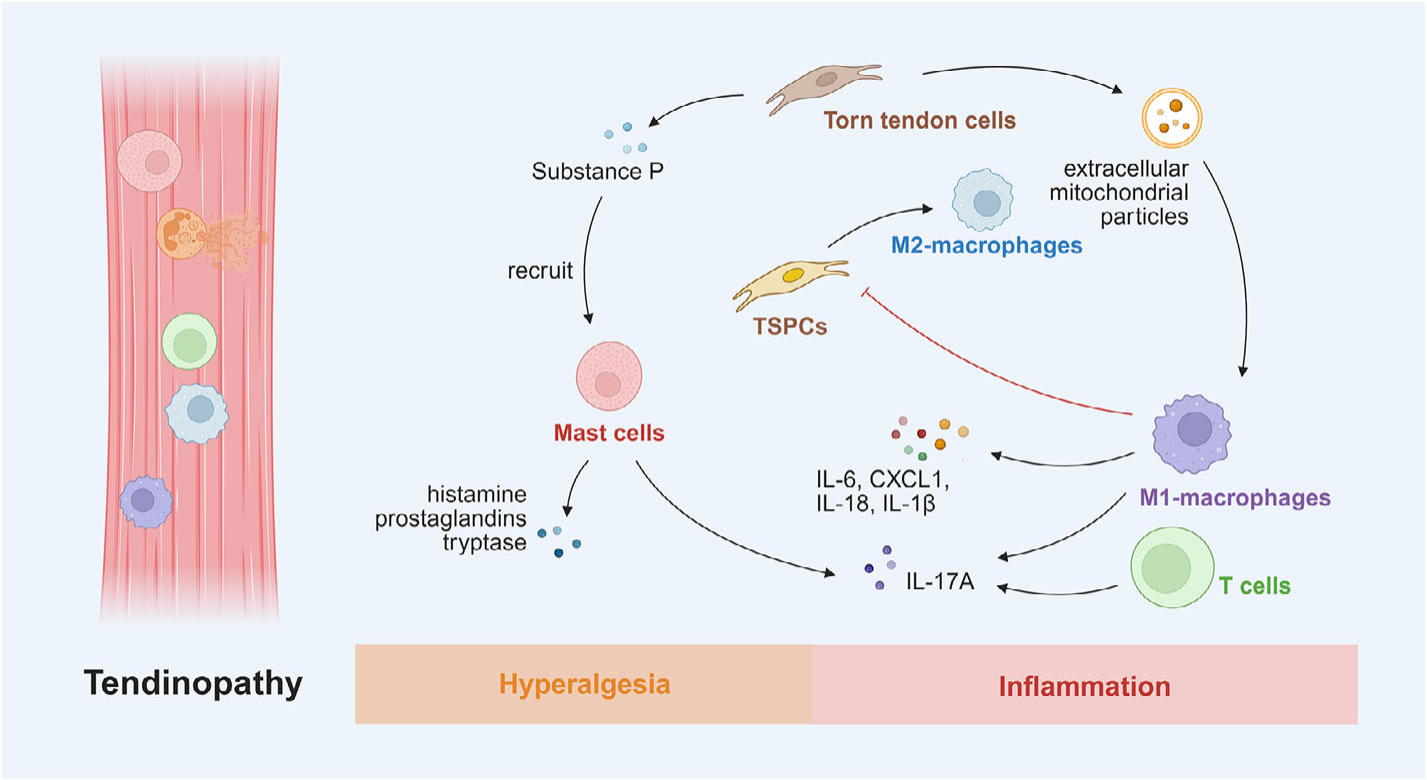
Mechanistic overview of immune cell contributions to tendinopathy. Extracellular mitochondrial particles released from mechanically overloaded tendon cells recruit and activate inflammatory macrophages, driving IL‐6, CXCL1, and IL‐18 production and enhancing chemotaxis. Infiltrating M1 macrophages amplify inflammatory signaling, impair TSPC differentiation, and release IL‐1β via NF‐κB/NLRP3 pathways, exacerbating degeneration; together with mast cells and T cells, they also promote IL‐17A production, further amplifying inflammation, stimulating type III collagen synthesis, and disrupting matrix remodeling. Mast cells, recruited by substance P, contribute to inflammation and pain through IL‐17A secretion and nerve interactions, while degranulation releases histamine, prostaglandins, and tryptase, inducing hyperalgesia, fibrosis, and neovascularization. Mast cell‐derived mediators further upregulate COX‐2/PGE2 and MMPs in tenocytes, suppressing type I collagen synthesis and accelerating ECM degradation. Neutrophils infiltrate peritendinous tissue early after injury, initiating the inflammatory cascade. In contrast, CD146⁺ TSPCs promote M2 macrophage polarization and repair, but diseased tendons show reduced M2 populations, sustaining a proinflammatory milieu. *Abbreviations*: NF‐κB, nuclear factor kappa B; NLRP3, NOD‐, LRR‐, and pyrin domain‐containing protein 3; TSPCs, tendon stem/progenitor cells; COX‐2, cyclooxygenase‐2; PGE2, prostaglandin E2; MMPs, matrix metalloproteinases; ECM, extracellular matrix.

Tendinopathy is a complex, multifactorial tendon disorder characterized by pain and functional impairment, most often resulting from overuse injuries. The body regions most frequently affected include the shoulder, elbow, knee, hip, and heel [11]. Healthy tendons are low‐metabolism, low‐cellularity tissues primarily composed of collagen‐producing tenocytes, with stem/progenitor cells, endothelial cells, and resident immune cells supporting tissue homeostasis and repair [367]. The pathophysiology of tendinopathy involves aberrant healing responses to repetitive microtrauma, manifested by disorganized collagen fibers and an abnormal increase in type III collagen [368]. Crucially, immune cells and inflammatory mediators play key roles in both the initiation and progression of this degenerative condition (Figure 5).

#### 4.5.1 Immuno‐Stimulatory Factors in Tendinopathy

##### 4.5.1.1 M1 Macrophages

Among immune cells, macrophages are the most extensively studied in the context of tendinopathy. Increased infiltration of CD68⁺ macrophages has been reported in tendinopathic tendons compared with healthy controls [369]. Notably, macrophage infiltration is already evident in early tendinopathy, as demonstrated in patellar tendon samples from early disease [370] and in subscapularis tendon samples obtained from patients with a torn supraspinatus tendon [371]. In cases with calcific tendinopathy, macrophages accumulate at the sites of calcific deposits, underscoring their roles in both inflammation and tissue remodeling [372]. Mechanistically, recruitment and activation of inflammatory macrophages are driven by extracellular mitochondrial particles released from mechanically overloaded tendon cells, which enhance proinflammatory cytokine production (IL‐6, CXCL1, IL‐18) and chemotaxis [373]. Recruited M1 macrophages, particularly the CCL4L2⁺ subset, display heightened inflammatory activity, amplify proinflammatory signaling, and impair TSPC differentiation [374]. Furthermore, M1 macrophages exacerbate tendon inflammation and degeneration by producing IL‐1β through NF‐κB activation and NLRP3 inflammasome signaling [375]. In addition, inflammatory macrophages contribute to IL‐17A production in tendinopathic tissues, together with mast cells and T cells. IL‐17A further amplifies inflammation, stimulates type III collagen synthesis, and disrupts matrix remodeling in tenocytes [376]. Pharmacological inhibition of M1 macrophages has shown therapeutic potential. For instance, pristimerin, a quinone methide triterpenoid, alleviates tendinopathy by promoting autophagic degradation of AIM2 (absent in melanoma 2) in a PYCARD/ASC (PYD and CARD domain containing/apoptosis‐associated speck‐like protein containing a CARD)‐dependent manner [377]. Taken together, these findings highlight that macrophage activation and polarization are central to the pathogenesis of tendinopathy. Thus, precise modulation of macrophage phenotypes represents a promising therapeutic strategy to attenuate inflammation and preserve tendon function.

##### 4.5.1.2 Mast Cells

Mast cell infiltration has been reported in multiple tendinopathies, including supraspinatus tears [371] and chronic nonruptured Achilles tendinopathy [369]. These cells, potentially recruited by neuropeptides such as substance P released from tenocytes or peripheral nerves [378], contribute to local inflammation by producing IL‐17A [376] and mediate pain through interactions with nerve fibers. Mast cell degranulation releases histamine, prostaglandins, and tryptase, which induce hyperalgesia [378, 379]. Additionally, mast cell‐derived tryptase acts on tenocytes and vascular structures via protease‐activated receptors, promoting fibrosis and neovascularization [378]. This proangiogenic role is supported by the correlation between mast cell density and CD34⁺ vascular markers in early human tendinopathy [371]. Mast cells also exacerbate tendon pathology by stimulating cyclooxygenase‐2/prostaglandin E2 production in tenocytes, which suppresses type I collagen synthesis, and by upregulating MMP1 and MMP7, enhancing ECM degradation and remodeling [380]. Importantly, small extracellular vesicles derived from induced pluripotent stem cell‐derived MSCs alleviate tendinopathy‐associated pain by inhibiting mast cell infiltration, degranulation, and cytokine release via suppression of hypoxia‐inducible factor signaling [379], highlighting a potential therapeutic strategy. In summary, mast cells represent a key target for early intervention and pain management in tendinopathy.

##### 4.5.1.3 Neutrophils

Although studies specifically addressing neutrophils in tendinopathy are limited, existing evidence highlights their involvement in disease pathogenesis. In rotator cuff tendinopathy, proteins with upregulated phosphorylation sites are predominantly associated with neutrophil‐mediated immunity, suggesting that neutrophil activation and related signaling pathways contribute to the inflammatory microenvironment [381]. In a rat model of Achilles tendinopathy, CD11b⁺ neutrophils were rapidly recruited to the peritendinous tissue, appearing as early as day 1 following elastase or collagenase injection, particularly under treadmill exercise. This early neutrophil infiltration indicates that these cells play a pivotal role in initiating the inflammatory cascade in response to ECM disruption and mechanical stress [382]. Nonetheless, the precise mechanisms underlying their recruitment and activation remain underexplored.

#### 4.5.2 Immuno‐Suppressive Factors in Tendinopathy

**M2 Macrophages**

In contrast to their proinflammatory M1 counterparts, M2 macrophages act to restrain excessive immune activation and facilitate tissue repair. In tendinopathy, CD146⁺ TSPCs, recruited to the paratenon following tendon injury, have been shown to promote macrophage polarization toward an M2 phenotype [383]. Nevertheless, diseased tendons typically exhibit a relative reduction in M2 macrophages, reflecting a shift toward a persistently proinflammatory microenvironment [374]. Furthermore, single‐cell and spatial transcriptomics have identified an SPP1⁺ macrophage subset with M2‐like, but not classical M2, characteristics. These cells are abnormally enriched in lesioned tendons and contribute to tendon heterotopic ossification, suggesting that aberrant M2‐like responses may also drive maladaptive tissue remodeling [384].

Modulation of macrophage polarization—particularly promoting M2 phenotypes—has shown therapeutic potential in tendinopathy. One promising strategy is the use of cell‐derived exosomes. For instance, DC‐derived exosomes promote tendon repair by inducing M1‐to‐M2 polarization through activation of the phosphatidylinositol 3 kinase (PI3K)/AKT pathway [385]. Similarly, adipose stem cell‐derived extracellular vesicles, especially those from young donors, enhance macrophage M2 polarization via the nicotinamide phosphoribosyltransferase/sirtuin 1/NF‐κB/NLRP3 pathway, thereby strengthening macrophage–tenocyte crosstalk and facilitating tendon repair [386, 387]. Large extracellular vesicles derived from induced pluripotent stem cell‐derived MSCs exert comparable effects by delivering dual‐specificity phosphatases 2/3, which suppress p38 MAPK signaling and promote M2 polarization [388]. In addition, an injectable tendon‐derived decellularized ECM hydrogel has been found to skew macrophages toward the M2 phenotype and exert anti‐inflammatory effects in vivo [389]. A lipid–polymer hybrid nanoparticle system coloaded with budesonide and serpine1 siRNA has also been used to target macrophages, promoting M2 polarization and exerting both immunomodulatory and antifibrotic effects in tendinopathy [390]. In conclusion, although the molecular mechanisms underlying M2 macrophage‐mediated tendon regeneration remain to be fully elucidated, emerging polarization‐targeted strategies underscore their promise in advancing clinical management of tendinopathy.

## 5 Neoplastic MSDs

Neoplastic MSDs, including osteosarcoma, chondrosarcoma, Ewing sarcoma, and STSs, comprise a group of malignancies arising from bone and soft tissues. Their pathogenesis frequently involves a critical failure of antitumor immunity, in which tumor cells evade immune surveillance by establishing an immunosuppressive microenvironment. A thorough understanding of these immune‐evasive mechanisms is essential for the development of novel immunotherapeutic strategies.

### 5.1 Osteosarcoma

**FIGURE 6:**
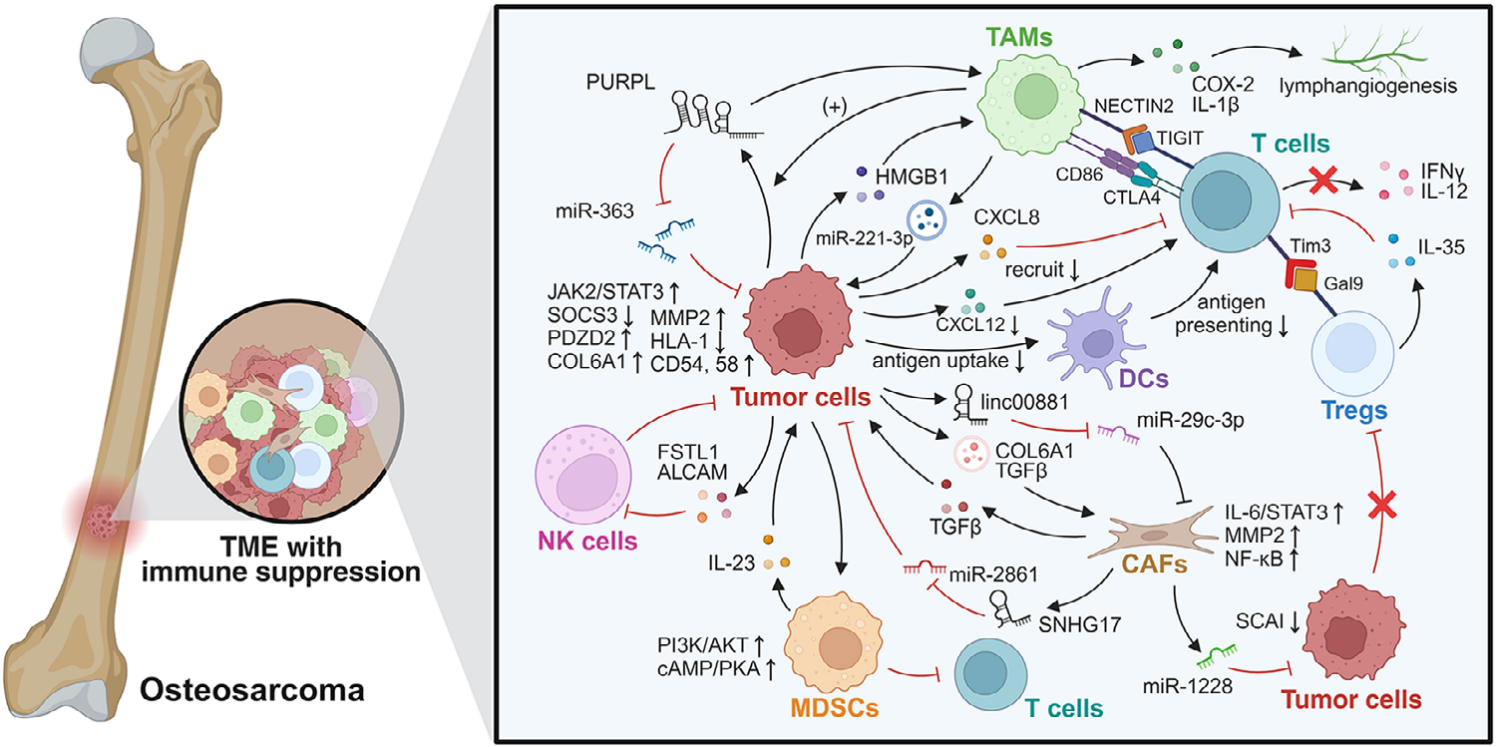
Mechanisms of immune evasion in the osteosarcoma microenvironment. T cell recruitment impaired by epigenetic silencing of CXCL12 in osteosarcoma cells. Osteosarcoma cells upregulate CXCL8, inducing PD‐L1 expression on CD8⁺ T cells and suppressing cytotoxicity. NK cells recognize tumor cells via HLA class I downregulation and CD54/CD58 upregulation. FSTL1 overexpression in osteosarcoma induces NK cell apoptosis and activates the ALCAM–CD6 immunosuppressive axis. DC dysfunction linked to immune evasion. TAMs promote tumor progression via exosomal miR‐221‐3p (↓SOCS3, ↑JAK2/STAT3), NECTIN2–TIGIT and CD86–CTLA4 suppression of T cells, secretion of COX‐2/IL‐1β for lymphangiogenesis, and stimulation of PURPL release (↓miR‐363, ↑PDZD2). HMGB1 and PURPL from tumor cells drive TAM polarization. CXCL12/CXCR4 chemotaxis recruits MDSCs and promotes survival via PI3K/AKT signaling; cAMP/PKA pathway further activates MDSCs. Activated MDSCs inhibit T cells and secrete IL‐23 to promote tumor growth. Tregs suppress IFNγ and IL‐12 production via IL‐35 and Gal9–Tim3 interaction. Osteosarcoma promotes CAF differentiation via exosomal COL6A1 and TGFβ, and through linc00881 (↓miR‐29c‐3p, ↑NF‐κB). Activated CAFs secrete TGF to upregulate COL6A1, promoting EMT and metastasis. CAF‐derived SNHG17 (↓miR‐2861) upregulates MMP2; miR‐1228 downregulates SCAI to enhance invasion. *Abbreviations*: PD‐L1, programmed death‐ligand 1; HLA, human leukocyte antigen; FSTL1, follistatin‐like 1; ALCAM, activated leukocyte cell adhesion molecule; TAMs, tumor‐associated macrophages; SOCS3, suppressor of cytokine signaling 3; JAK2, Janus kinase 2; STAT3, signal transducer and activator of transcription 3; NECTIN2, nectin cell adhesion molecule 2; TIGIT, T cell immunoreceptor with Ig and ITIM domains; CTLA4, cytotoxic T‐lymphocyte‐associated protein 4; COX‐2, cyclooxygenase‐2; PURPL, p53 upregulated regulator of p53 levels; PDZD2, PDZ domain containing 2; HMGB1, high‐mobility group box 1; MDSCs, myeloid‐derived suppressor cells; PI3K, phosphoinositide 3‐kinase; AKT, protein kinase B; cAMP, cyclic adenosine monophosphate; PKA, protein kinase A; IFNγ, interferon gamma; Gal9, galectin‐9; Tim3, T cell immunoglobulin and mucin domain‐containing protein 3; CAF, cancer‐associated fibroblast; COL6A1, collagen type VI alpha 1 chain; TGFβ, transforming growth factor beta; NF‐κB, nuclear factor kappa B; SNHG17, small nucleolar RNA host gene 17; MMP2, matrix metalloproteinase 2; SCAI, suppressor of cancer cell invasion.

Osteosarcoma is the most common primary malignant bone tumor, with peak incidence occurring in children and young adults [391]. The high recurrence rate and limited overall survival (OS) achieved with conventional therapies [392] underscore the urgent need for novel treatment strategies, including targeted drug delivery systems, molecularly targeted therapies, and approaches that modulate the immune microenvironment. The tumor microenvironment (TME) of osteosarcoma is widely recognized as profoundly immunosuppressive, being dominated by TAMs (tumor‐associated macrophages), MDSCs, and Tregs. Meanwhile, immune effector cells—including CTLs, NK cells and M1‐polarized macrophages—also infiltrate the tumor, albeit often insufficiently to overcome the prevailing immunosuppressive milieu. These cells collectively define the overall immune status of osteosarcoma tissues and are closely associated with patient prognosis and survival [393] (Figure 6).

#### 5.1.1 Immuno‐Stimulative Factors in Osteosarcoma

##### 5.1.1.1 T Cells

Though central to antitumor immunity in many malignancies, T cells appear to be relatively inactive and poorly recruited in the context of osteosarcoma. In particular, CD8^+^ cytotoxic T cells frequently exhibit functional exhaustion, characterized by high expression of inhibitory receptors such as PD‐1 and TIM‐3 (T cell immunoglobulin and mucin‐domain containing‐3), likely resulting from the immunosuppressive influence of TAMs [393]. One mechanism underlying the poor recruitment of CD8^+^ T cells involves epigenetic silencing of CXCL12, a key chemokine that mediates T cell trafficking via the CXCL12/CXCR4 signaling axis. This silencing is driven by DNMT1 (DNA methyltransferase 1)‐mediated DNA hypermethylation in osteosarcoma cells [394]. Conversely, osteosarcoma cells may upregulate CXCL8, which enhances PD‐L1 expression on CD8^+^ T cells and further suppresses their cytotoxic function [395]. However, the precise mechanisms underlying T cell exclusion, particularly the contributions of tumor–stroma interactions and stromal‐derived physical barriers, remain insufficiently understood and merit further investigation.

Efforts to restore T cell activity in osteosarcoma have shown promising results. For example, MYC inhibition enhances T cell infiltration and activates the CD40–CD40L costimulatory axis [396], whereas TLR4 stimulation effectively augments CD8^+^ T cell activity [397]. Moreover, adoptive cell therapies are currently under active investigation. Memory T cells engineered with NKG2D‐CARs (chimeric antigen receptors) exhibit strong cytotoxicity [398], and in vitro‐expanded tumor‐infiltrating lymphocytes (TILs) have shown efficacy in preclinical models [399]. In addition, increasing attention has been directed toward unconventional cytotoxic T cell subsets in osteosarcoma immunotherapy. Notably, gamma‐delta T cells, featuring a distinct TCR composed of gamma and delta chains and a characteristic lack of CD4 and CD8 expression, have also demonstrated antitumor potential [400] and may offer an alternative immunotherapeutic strategy, particularly in cases where conventional T cell‐based therapies fail.

##### 5.1.1.2 NK Cells

The innate immune system serves as a crucial first line of defense against tumor development. While macrophages are typically identified as TAMs facilitating tumor progression, NK cells are involved in immune surveillance and antitumor immunity. Clinical studies have reported reduced NK cell infiltration in osteosarcoma patients, particularly those with poor prognosis [401], although active NK cells have been observed in osteosarcoma‐associated malignant pleural effusions [402]. Therapies aimed at NK cell stimulation, such as IL‐2 administration, have been associated with improved clinical outcomes [403]. Recognition of osteosarcoma cells by NK cells is closely tied to the downregulation of HLA class I [404] and altered expression of adhesion molecules (e.g., CD54, CD58) on tumor cells [403]. Mechanistically, osteosarcoma cells evade NK cell–mediated cytotoxicity through overexpression of follistatin‐like 1, which induces NK cell apoptosis and activates the immunosuppressive activated leukocyte cell adhesion molecule–CD6 immunosuppressive axis [405]. Notably, blockade of CD155, another inhibitory ligand expressed on tumor cells, enhances DNAM‐1 (DNAX accessory molecule‐1)‐mediated NK cell activation and cytotoxicity following allogeneic bone marrow transplantation, further supporting the therapeutic potential of targeting NK cell regulatory pathways [406]. Together, these findings highlight the significant role of NK cells in osteosarcoma and highlight the therapeutic promise of strategies aimed at restoring their effector function.

##### 5.1.1.3 DCs

As key antigen‐presenting cells (APCs), DCs initiate antitumor immunity by presenting antigens and activating T cells. Although they comprise less than 5% of all myeloid cells recruited into the osteosarcoma TME [407], their functional impairment is closely linked to disease progression and immune evasion [393]. The subclassification of DCs remains an important yet context‐dependent area of ongoing research. Commonly identified DC subsets include conventional DCs (cDCs), plasmacytoid DCs, and LAMP3^+^ mature regulatory DCs (mregDCs) [408, 409]. Within the osteosarcoma TME, cDC2s (CD1c^+^CLEC10A^+^ DCs) are more abundant than cDC1s (XCR1^+^CLEC9A^+^ DCs) and mregDCs [409]. In addition, cDC2s have been explored as a source for vaccine‐based immunotherapy [407], demonstrating promising immunologic and clinical outcomes and representing a viable therapeutic target. In contrast, mregDCs play an immunosuppressive role, likely through interactions with Tregs mediated by CD274/PD‐1 and poliovirus receptor/TIGIT signaling [408]. Collectively, these findings underscore the dual roles of DC subpopulations in osteosarcoma immunity and highlight their potential in future immunotherapeutic strategies.

#### 5.1.2 Immuno‐Suppressive Factors in Osteosarcoma

##### 5.1.2.1 TAMs

TAMs represent the most abundant immune cell population within the osteosarcoma TME, predominantly exhibiting an M2‐polarized or “alternatively activated” phenotype [410, 411]. These immunosuppressive M2 macrophages outnumber their proinflammatory M1‐polarized counterparts [402, 412] and are frequently clustered around blood vessels [413]. Notably, a dynamic shift from M1 to M2 polarization occurs within the TME [402], reflecting a transition toward an immunosuppressive milieu. TAMs promote osteosarcoma progression and metastasis through multiple mechanisms, including exosome‐mediated signaling [414], suppression of T cell cytotoxicity [402], enhancement of tumor lymphangiogenesis [415], and formation of physical barriers that impede immune cell infiltration [416]. In addition, reciprocal interactions between TAMs and tumor cells further drive malignancy. For example, He et al. [417] discovered that TAMs facilitate the secretion of the self‐protective long noncoding RNA (lncRNA) PURPL (p53 upregulated regulator of p53 levels) from osteosarcoma cells, which in turn promotes tumor proliferation and reinforces TAM recruitment and polarization. Collectively, these findings underscore the central role of TAMs in remodeling the osteosarcoma TME and driving malignant progression. Targeting TAM‐tumor interactions may therefore represent a promising therapeutic avenue for osteosarcoma intervention.

##### 5.1.2.2 MDSCs

MDSCs, characterized by their immunosuppressive properties, have been long implicated as key contributors to tumorigenesis since their initial characterization [418, 419, 420]. In osteosarcoma, elevated levels of MDSCs have been observed in both primary [421] and metastatic tissues [416], as well as in peripheral blood [422, 423]. Single‐cell RNA sequencing in osteosarcoma‐bearing dogs has revealed the presence of both G‐MDSC and M‐MDSC subtypes, accompanied by widespread transcriptional dysregulation in myeloid compartments [424].

MDSC recruitment and activation are essential for their tumor‐promoting function in osteosarcoma. IL‐18 has been identified as a key driver of MDSC infiltration into the osteosarcoma TME [422], while the CXCR4/CXCL12 chemokine axis and PI3K/AKT signaling are critical for their chemotaxis and functional activation [421]. Notably, preclinical models have demonstrated that combining CXCR4 antagonists with anti‐PD‐1 antibodies yields synergistic antitumor effects [421]. Moreover, neoadjuvant chemotherapy has been shown to significantly reduce MDSC accumulation at tumor sites [425], indicating their potential susceptibility to cytotoxic agents.

Tumor cells actively contribute to MDSC activation via the cAMP/protein kinase A pathway, leading to enhanced secretion of the protumorigenic cytokine IL‐23 [426]. Upon activation, MDSCs effectively suppress T cell expansion, infiltration and cytotoxic function, thereby enabling immune evasion and facilitating osteosarcoma progression [421, 423]. These observations collectively underscore the pivotal role of MDSCs in shaping the immunosuppressive landscape of the osteosarcoma microenvironment.

##### 5.1.2.3 Tregs

The abundant infiltration of Tregs in osteosarcoma tissues, together with their association with poor prognosis [427, 428], implicates their critical role in osteosarcoma pathophysiology. However, emerging studies suggest that it is not merely the quantity, but the functional status of Tregs that drives disease progression [429]. Mechanistically, Tregs promote osteosarcoma cell proliferation primarily by suppressing tumor‐eliminating immune subsets, including CD4^+^ and CD8^+^ T cells as well as monocytes. For example, Treg‐derived cytokine IL‐35 impairs both cytolytic and noncytolytic activities of CD8^+^ T cells [430]. In addition, Treg‐expressed Gal9 inhibits the secretion of IFNγ and IL‐12 from CD4^+^ T cells and monocytes, respectively, via Gal9/Tim3 interactions [431]. Importantly, therapeutic strategies targeting Tregs—including antibodies against PD‐1 [432, 433], CD25 [434], CD40 [435], and CTLA‐4 [432]—have demonstrated promising clinical potential in the treatment of osteosarcoma. Further investigations into Treg‐specific mechanisms may offer novel targets to disrupt the immune‐privileged niche supporting tumor progression.

##### 5.1.2.4 Cancer‐Associated Fibroblasts

Cancer‐associated fibroblasts (CAFs) are specialized fibroblasts within the TME, originating from diverse sources including tissue‐resident fibroblasts, recruited mesenchymal cells, adipocyte‐derived precursor cells, endothelial cells, mesothelial cells, and pericytes [436]. CAFs exhibit remarkable heterogeneity, and their roles in tumor progression can be paradoxical [437]. Traditionally, CAFs have been viewed as tumor‐promoting owing to their secretion of immunosuppressive cytokines and exosomes [438], but recent evidence has also identified immunostimulatory CAF subsets capable of restraining tumor development [439, 440]. In osteosarcoma, CAF activation is frequently induced by tumor‐derived factors, such as COL6A1 [441] and TGFβ [442]. Activated CAFs are characterized by robust IL‐6/STAT3 signaling activity [443]. Moreover, tumor cells can modulate CAF behavior through noncoding RNAs. For example, osteosarcoma‐derived lncRNA linc00881 regulates the expression of MMP2 in lung fibroblasts by competing with miR‐29c‐3p, thereby activating the NF‐κB signaling pathway and facilitating CAF differentiation [444].

Despite increasing attention, the reciprocal effects of CAFs on osteosarcoma progression remain incompletely understood. Interestingly, recent single‐cell RNA sequencing studies have begun to unravel osteosarcoma‐specific CAF subtypes. For instance, Zhou et al. [407] identified three distinct CAF clusters characterized by the expression of COL14A1, smooth muscle‐like traits, and osteoblast‐like myofibroblastic phenotypes, respectively. Xu et al. [445] further delineated six CAF subtypes, with TOP2A (DNA topoisomerase II alpha)‐high cells exhibiting the greatest oncogenic potential and most frequent tumor–stroma interactions. These CAF subpopulations appear to exhibit differential associations with osteosarcoma metastasis and tumor–stroma crosstalk [407, 445]. Together, these findings highlight the functional diversity of CAFs and their emerging relevance in osteosarcoma biology.

##### 5.1.2.5 Tumor‐Associated Neutrophils

Tumor‐associated neutrophils (TANs) exhibit remarkable plasticity and actively participate in the progression of malignancies [446]. They are phenotypically classified into antitumor subtype TAN1 and protumor subtype TAN2 [447]. TAN1 cells are recruited to tumor sites by G‐CSF, while TAN2 cells are activated by tumor‐derived cytokine TGFβ and contribute to the establishment of an immunosuppressive TME [448]. Transition of TAN2 to TAN1 can be facilitated by IFNγ and TNFα [449]. Despite the intense research focus on TANs, their roles in primary bone tumors remain poorly characterized. In osteosarcoma, pioneering work by Tan and Chao [450] identified TAN infiltration in both metastatic and recurrent lesions, with markedly higher accumulation in metastatic sites. These findings suggest a potential involvement of TANs in osteosarcoma progression and metastasis, although further mechanistic studies are needed.

### 5.2 Chondrosarcoma

Chondrosarcoma is the second most common bone malignancy, arising from cartilage‐like tissue of mesenchymal origin. It can be classified as either primary or secondary, the latter resulting from malignant transformation of benign cartilage tumors such as enchondromas or osteochondromas [451]. Surgical resection has long remained the only effective treatment, as both chemotherapy and radiotherapy have shown limited efficacy [452]. Within the chondrosarcoma TME, cytotoxic CD8^+^ T cells and CD163^+^ TAMs represent the two predominant immune populations, predominantly localized at the tumor margins and exerting opposing effects on tumor progression. Disruption of CD8^+^ T cells accelerates tumor growth, while depletion of CD163^+^ TAMs inhibits tumor progression [453]. In a cohort of 98 newly diagnosed chondrosarcoma patients, Li et al. [452] identified three immunologically distinct subtypes: subtype I dominated by G‐MDSCs and HLA‐DR^−^ CD14^−^ myeloid cells; subtype II characterized by an immune‐exhausted phenotype; and subtype III presenting minimal immune infiltration (immune‐desert type). These findings underscore the heterogeneity and complexity of the chondrosarcoma immune microenvironment, suggesting the need for stratified immunotherapeutic approaches.

#### 5.2.1 Immuno‐Stimulative Factors in Chondrosarcoma

**T Cells**

Research on immunostimulatory factors in chondrosarcoma remains limited, with T cell infiltration within the TME being one of the few reported aspects. A positive correlation has been observed between T cell infiltration and PD‐L1 expression on tumor cells, accompanied by clear spatial colocalization. In a study involving 22 chondrosarcoma samples, 45% showed substantial T cell infiltration. Notably, several cases exhibited significantly higher T cell density in peritumoral region than in intratumoral areas, indicating a state of immune exclusion [454]. This phenomenon should be carefully considered in future investigations of checkpoint blockade therapies for chondrosarcoma.

#### 5.2.2 Immuno‐Suppressive Factors in Chondrosarcoma

**TAMs**

TAMs constitute the most abundant immune population within the TME of chondrosarcoma and, so far, represent the only immunosuppressive population that has been partially explored in this context. Previous studies have revealed a strong correlation between the density of CD68^+^ TAMs and both metastasis‐free and progression‐free survival (PFS). Moreover, the CD68^+^/CD8^+^ ratio also influence prognosis, indicating the protective role of T cells and the tumor‐promoting function of TAMs [455]. Interestingly, TAMs are predominantly localized in dedifferentiated regions of chondrosarcoma, whereas they are rarely observed in well‐differentiated areas [454, 456]. Recent findings indicate that TAMs can be activated by chondrosarcoma‐derived exosomal lncRNA TUG1 (taurine upregulated gene 1), and in turn facilitates the proliferation and metastasis of chondrosarcoma [457]. Considering the significant role of TAMs in the pathophysiology of chondrosarcoma, Quoniou et al. [458] developed a three‐dimensional tumoroid coculture model combining chondrosarcoma and leukemic monocytic cell lines. Within this model, an immunomodulatory agent targeting TAMs was shown to exert therapeutic effects against chondrosarcoma [458]. This approach provides a promising and intuitive platform for drug screening and personalized treatment strategies in chondrosarcoma. Taken together, the immunosuppressive landscape of chondrosarcoma remains underexplored and warrants further investigation.

### 5.3 Ewing Sarcoma

Ewing sarcoma is the second most common primary bone malignancy in children, following osteosarcoma, and characterized by high aggressiveness and frequent metastatic presentation at diagnosis. A hallmark of Ewing sarcoma is the presence of oncogenic chimeric fusion genes, most commonly involving Ewing sarcoma breakpoint region 1 and Friend leukemia integration 1, which serve as key biomarkers in disease management [459]. Recent studies have identified a distinct subpopulation of CD73^+^ tumor cells exhibiting CAF‐like characteristics in Ewing sarcoma. These cells are characterized by abundant ECM deposition and play a crucial role in TME remodeling, thereby potentially facilitating tumor invasion [460]. In addition, accumulating evidence suggests that immune cell infiltration substantially contributes to the pathogenesis of Ewing sarcoma, highlighting the importance of the immune landscape in disease progression.

#### 5.3.1 Immuno‐Stimulative Factors in Ewing Sarcoma

**T Cells**

Unlike many other malignancies, Ewing sarcoma is regarded as an “immune‐cold” tumor, primarily due to its low CD8^+^ T cell abundance and minimal PD‐L1 expression. Most studies examining these features have reported limited or no prognostic significance, reinforcing the concept of an immunologically inert TME [461]. The paucity of TILs, particularly T cells, may stem from deficient HLA expression on tumor cells, which is essential for effective adaptive immune responses [462]. However, recent research has increasingly focused on converting the immune microenvironment from “cold” to “hot,” offering a promising strategy to enhance the efficacy of immunotherapy. For example, Henrich et al. [463] demonstrated the tumor‐suppressive role of ubiquitin‐specific protease 6, highlighting the potential of immune modulation in the therapeutic management of Ewing sarcoma.

#### 5.3.2 Immuno‐Suppressive Factors in Ewing Sarcoma

##### 5.3.2.1 TAMs

Although TAMs constitute a smaller fraction of immune cells in Ewing sarcoma than in osteosarcoma, their infiltration correlates positively with poor prognosis. In a comparative study involving 20 samples each of Ewing sarcoma and osteosarcoma, the median percentage of CD163^+^/CD68^+^ M2‐polarized TAMs was approximately 3% of the tumor area in Ewing sarcoma, notably lower than the ∼15% observed in osteosarcoma [412]. TAMs are also implicated in mediating tumor resistance, in part by suppressing NK cell activity. However, the underlying mechanisms remain largely unclear [464]. Further studies are warranted to elucidate the roles of these key immune components in Ewing sarcoma progression.

##### 5.3.2.2 MDSCs

MDSCs remain insufficiently studied in Ewing sarcoma; nevertheless, existing evidence suggests their significant contribution to tumor progression [461]. A specialized MDSC subset with angiogenic capabilities, known as F2 fibrocytes, originates from healthy monocytes under IL‐4 stimulation and has been shown to proliferate markedly in metastatic Ewing sarcoma tissues. These cells strongly suppress T cell function through the production of indoleamine 2,3‐dioxygenase [465]. Moreover, targeting MDSCs holds therapeutic potential in Ewing sarcoma. For instance, trabectedin treatment suppresses the growth of Ewing sarcoma in murine models, an effect partly mediated by MDSC depletion and the inhibition of their differentiation into TAMs [466].

##### 5.3.2.3 Tregs

In Ewing sarcoma, elevated levels of Tregs correlate with an increased propensity for metastasis [467]. Accordingly, Tregs are widely regarded as effective biomarkers for evaluating responses to chemotherapy and immunotherapy [468], although their precise functions remain under investigation. For example, the combined treatment with oncolytic adenovirus XVir‐N‐31 and a cyclin‐dependent kinase 4/6 inhibitor effectively suppressed Ewing sarcoma growth both in vitro and in vivo, concomitant with a reduction in Treg infiltration [469].

### 5.4 Soft Tissue Sarcomas

STSs represent a heterogeneous group of mesenchymal malignancies arising from connective tissues. While surgery remains the cornerstone of treatment, high rates of local recurrence and distant metastasis continue to pose significant clinical challenges [470]. Accumulating evidence indicates that dynamic interactions between tumor cells and immune components within the TME play a pivotal role in facilitating tumor progression and adaptation to environmental stress [471]. Given the marked histological and biological heterogeneity of STSs, deciphering their immunological landscape has emerged as a critical priority for advancing therapeutic strategies and improving patient outcomes.

#### 5.4.1 Immuno‐Stimulatory Factors in STS

##### 5.4.1.1 T Cells

T cells play a central role in antitumor immunity across various STSs, acting as key effectors in controlling tumor progression. In the IMMUNOSARC trial, CD8⁺ T cells emerged as crucial drivers of therapeutic response, with their infiltration and activation forming a molecular signature predictive of clinical outcomes in sarcoma [472]. High infiltration of CD4⁺ T cells correlates with better prognosis in dedifferentiated liposarcoma [473], and elevated densities of CD3⁺ and CD8⁺ tumor‐infiltrating T cells—particularly when combined with low NLR—are associated with improved outcomes in pulmonary metastases of uterine leiomyosarcoma [474]. Adoptive transfer of in vitro‐expanded tumor‐infiltrating T cells, predominantly CD8⁺, has demonstrated efficacy in autologous murine models of liposarcoma [399], highlighting the therapeutic potential of T cell–based interventions in STSs. Importantly, certain STS subtypes, including gastrointestinal stromal tumor (GIST), myxofibrosarcoma, and pleomorphic sarcoma, harbor abundant CD8⁺ T cells; however, in GIST, these T cells are less differentiated, insufficiently activated, and express low levels of costimulatory ligands [475]. In addition, neoadjuvant radiotherapy in undifferentiated pleomorphic sarcoma (UPS) induces cytotoxic T cell infiltration, whereas myxofibrosarcoma shows minimal T cell recruitment [476], emphasizing the heterogeneity of T cell‐mediated immunity and the necessity of tailored immunotherapeutic approaches. These findings underscore that improving T cell activation and recruitment is a central strategy for enhancing treatment efficacy in STSs.

Impaired T cell function arises from multiple immunosuppressive mechanisms, reflecting the complex interplay between tumors and the immune system. Spatial profiling has linked tumor‐intrinsic Wnt/β‐catenin activation to T cell exclusion, compromising the efficacy of transgenic T‐cell therapy in synovial sarcoma [477]. In UPS, ECM remodeling drives CD8⁺ T cell dysfunction: Yes‐associated protein 1‐mediated collagen VI deposition induces immune evasion, whereas collagen I counteracts this effect to support antitumor immunity [478]. In leiomyosarcoma lung metastases, epithelial cellular adhesion molecule upregulation inhibits CD8⁺ T cell migration [479], indicating that structural and adhesion molecules within the TME directly regulate T cell access and activity. Conversely, PI3K/mTOR inhibition promotes infiltration and reinvigoration of exhausted PD‐1⁺CD8⁺ T cells, sustains a CD4⁺ Th1 niche, amplifies cytotoxic T cell responses, and sensitizes uterine leiomyosarcomas to PD‐1 blockade [480]. Transforming acidic coiled‐coil containing protein promotes CD8⁺ T cell infiltration and activation via upregulation of CCL3 and CCL4, strengthening antitumor immunity and improving PD‐1 blockade efficacy [481]. Similarly, RIPK1 (receptor‐interacting protein kinase 1)‐induced immunogenic cell death of tumor cells activates CD8⁺ T cells and augments checkpoint blockade efficacy in STSs [482]. Together, these mechanisms illustrate that both tumor‐intrinsic and microenvironmental factors dictate T cell functionality, emphasizing their central therapeutic relevance.

Although immunosuppressive factors, such as tumor PD‐L1 expression, impair effector T cell cytotoxicity, higher posttreatment densities of intratumoral and peritumoral T cells—including intratumoral PD‐1⁺ CD8⁺ subsets—correlate with improved disease‐free survival, highlighting the central prognostic and therapeutic role of T cells [483]. These findings reinforce that strategies preserving or restoring T cell effector functions may yield substantial clinical benefits in STSs. Collectively, the active involvement of T cells in STS antitumor immunity, coupled with their suppressed functional state, underscores their potential as both biomarkers and therapeutic targets, positioning T cells at the core of future STS immunotherapy development.

##### 5.4.1.2 NK Cells

NK cells are innate immune effectors critical for antitumor responses. In STSs, NK cells can be activated by RIPK1‐induced immunogenic cell death of tumor cells, contributing to tumor control [482]. However, patients with STSs often display impaired NK‐mediated immunity, characterized by reduced CD56^dim^ NK cells and lower expression of perforin 1, GZMB, and killer cell lectin‐like receptor K1/NKG2D, correlating with unfavorable immune phenotypes and poorer survival [484]. This functional impairment is further associated with decreased degranulation, reduced IFNγ production, and altered receptor repertoire, including increased CD27 expression indicative of a regulatory NK cell phenotype [485]. Regulatory NK cells secrete IL‐6, which activates STAT3 signaling in MDSCs, thereby suppressing T cell responses and promoting tumor immune escape [486].

NK cell subsets also exert prognostic influence in STSs. Intratumoral CD56^dim^ and NKp46^+^ NK cells are linked to improved survival, whereas CD56^bright^ NK cells are associated with worse outcomes. Spatial analyses reveal that NK cells preferentially cluster near MHC‐I^–^ cells and other NK cells rather than T cells, highlighting their distinct organization within the TME [487]. Certain tumor models further demonstrate the capacity of NK cell activation. For instance, a novel UPS cell line, JBT19, stimulates NK cell expansion and cytotoxicity [488], while synovial sarcoma cells express ligands for NK‐activating receptors, including NKp44 and NKp30 [489]. Importantly, NK cell activity can be therapeutically enhanced. In humanized NSG mice xenografted with dedifferentiated liposarcoma, the efficacy of anti‐PD‐1 therapy was associated with the enrichment of activated CD56^+^ NK cells expressing NKp46 and NKG2D, indicating a pivotal role for NK cells in mediating antitumor effects [490]. Similarly, panobinostat suppresses STS cell growth by enhancing NK cell‐mediated cytotoxicity via upregulation of MICA/MICB (MHC class I chain‐related protein A/B) through increased histone acetylation at the β‐catenin promoter, highlighting its potential as an adjuvant to NK cell‐based immunotherapy [491]. In summary, NK cells in STS exhibit both functional impairment and subset‐dependent prognostic relevance, and strategies to activate NK cells, including immune checkpoint blockade and epigenetic modulation, hold considerable promise as promising therapeutic avenues.

##### 5.4.1.3 B Cells

The extent of B cell infiltration varies across STS subtypes. Retroperitoneal liposarcoma exhibits pronounced CD8⁺ T cell infiltration [492], whereas myxosarcomas display a B cell‐dominant immune milieu [493]. B cell abundance in STSs is also age‐dependent, as immunohistochemical analyses reveal reduced B cell proportions in patients older than 62.5 years [494]. The functions of B cells in STSs are increasingly recognized as being associated with favorable prognosis. CD20/membrane‐spanning 4‐domains subfamily A member 1⁺ B cells act as context‐dependent prognostic factors, linked to improved survival through antigen presentation and T cell activation, but only in tumors without a strongly immunosuppressive TME characterized by high IL‐10 expression [495]. Moreover, B cell infiltration has been identified as a key discriminative feature associated with prolonged survival and enhanced responsiveness to PD‐1 blockade with pembrolizumab [496]. In dedifferentiated liposarcoma, neoadjuvant treatment increases tumor‐associated B cell infiltration, which correlates with improved outcomes [497]. Similarly, findings from the DAPPER clinical trial in advanced leiomyosarcoma suggest that heightened B cell activity may identify patients more likely to benefit from immune checkpoint blockade [498]. Nevertheless, the mechanisms underlying B‐cell‐mediated immune modulation in STSs are still poorly defined, underscoring the need for mechanistic studies to translate their prognostic value into therapeutic applications.

#### 5.4.2 Immuno‐Suppressive Factors in STS

##### 5.4.2.1 Tregs

Tregs act as suppressors of antitumor immunity in STSs. Lower baseline densities of Tregs prior to treatment predict a higher likelihood of achieving a major pathological response [497]. In addition, FoxP3⁺ Treg infiltration correlates significantly with PD‐L1 expression, and both serve as independent adverse prognostic factors for survival, suggesting a synergistic role in tumor immune evasion [499]. In myxofibrosarcoma, Treg infiltration independently predicts a higher risk of local recurrence, irrespective of surgical margins [500]. Importantly, different STS subtypes exhibit distinct immune profiles: myxosarcomas elicit a B cell‐ and Treg‐rich immune milieu, whereas perivascular wall tumors display a T cell‐dominant, Treg‐poor immune phenotype [493]. Bioinformatic analyses indicate that the activation of the IL‐33/ST2 axis may counteract Treg‐ and MDSC‐mediated immunosuppression while promoting antitumor immunity [501]. Moreover, Treg presence can limit therapeutic efficacy in STSs, as expanded Treg populations restrict the effectiveness of PD‐1‐based combination therapies [502]. Targeting Treg‐mediated immunosuppressive mechanisms therefore remains a major challenge in designing future STS treatment strategies.

##### 5.4.2.2 MDSCs

MDSCs remain insufficiently characterized in STSs. Nevertheless, their prognostic significance as an immunosuppressive subset is increasingly recognized. For instance, elevated levels of M‐MDSCs in the peripheral blood of sarcoma patients are associated with poorer clinical outcomes [503]. Their increased frequency, along with higher expression of arginase 1 and immunosuppressive cytokines such as IL‐10, correlates with impaired cytotoxic activity of NK and CD8⁺ T cells [484], underscoring MDSCs as key mediators of immune evasion in STSs. In a clinical trial combining intratumoral administration of DCs with fractionated external beam radiation in high‐risk STS patients, MDSCs—but not regulatory T cells—were significantly enriched in nonresponders [504], highlighting their role in limiting tumor‐specific immune responses. Bioinformatic analyses further suggest that activation of IL‐33/ST2 axis may counteract MDSC‐mediated immunosuppression [501], indicating potential targets for intervention. Overall, the precise functions and therapeutic relevance of MDSCs in STSs warrant further investigation.

##### 5.4.2.3 TAMs

The presence of TAMs, typically displaying an M2‐like phenotype, is generally associated with adverse outcomes in STSs. High levels of TAM infiltration predict an increased risk of local recurrence [494], and TAM enrichment represents a hallmark of therapeutic nonresponse in STSs [505]. In both synovial sarcoma [506] and dedifferentiated liposarcoma [473], TAM infiltration correlates with unfavorable survival. The abundance and phenotypic characteristics of TAMs also vary according to tumor grade. For instance, high‐grade myxoid liposarcoma displays a highly vascularized architecture with pronounced CD163^+^ TAM infiltration, whereas low‐grade tumors contain few macrophages [507]. Interestingly, TAMs upregulate PD‐L1 following preoperative radiotherapy, and this increase is associated with a higher risk of distant metastasis, underscoring their potential role in mediating radiotherapy‐induced adverse effects [508]. Conversely, prechemotherapy TAM infiltration correlates with baseline tumor metabolic activity, predicting improved chemotherapy responsiveness [509], possibly reflecting the greater chemosensitivity of metabolically active tumor cells.

Bidirectional crosstalk between TAMs and STS cells further promotes tumor progression. STS cells express MIF, which activates TAMs through CD74 signaling [510]. In synovial sarcoma, PI3K‐driven M‐CSF secretion promotes TAM recruitment [511]. Myxoid liposarcoma cells release soluble factors that induce M2 polarization of TAMs, which in turn facilitate ECM invasion and transendothelial migration of tumor cells [507]. Liposarcoma‐derived extracellular vesicles carrying miR‐25‐3p and miR‐92a‐3p stimulate TAMs via TLR7/8, triggering IL‐6 secretion that enhances tumor proliferation, invasion, and metastasis [512]. In addition, STS cells frequently overexpress CD47, the canonical “don't eat me” signal, enabling immune evasion by engaging SIRPα (signal regulatory protein alpha) on macrophages [513]; notably, infiltration by SIRPα^+^ macrophages has also been linked to poor prognosis [514]. Collectively, TAMs emerge as central mediators of the immunosuppressive TME in STSs, collaborating with tumor cells to drive progression and therapeutic resistance. Concurrently, these cells represent promising therapeutic targets for future interventions.

##### 5.4.2.4 CAFs

Fibroblasts skewed by tumor‐derived factors play a pivotal role in driving STS proliferation. Elevated expression of CAF markers in both intratumoral and peripheral regions has been linked to poor prognosis in STS patients [515]. In liposarcoma, tumor‐derived thrombospondin‐2 activates CAFs, which subsequently promote tumor cell proliferation and migration via the MAPK/ERK signaling pathway [516]. Similarly, overexpression of minichromosome maintenance 2 (MCM2) in liposarcoma enhances CAF formation, thereby facilitating tumor proliferation, migration, and invasion [517]. In uterine leiomyosarcoma, tumor‐secreted extracellular vesicles containing miR‐654‐3p and miR‐369‐3p similarly induce CAF formation [518].

Beyond tumor–CAF interactions, crosstalk between CAFs and mast cells has also been implicated. In fibrosarcoma, tumor‐resident mast cells stimulate CAF proliferation and ECM stiffening, contributing to an immunosuppressive TME and therapeutic resistance. Pharmacological inhibition of mast cell activation with the antihistamine ketotifen suppresses CAF activity, reduced matrix stiffness, and improves vascular perfusion and oxygenation, thereby enhancing T cell infiltration and the efficacy of chemoimmunotherapy [519]. Moreover, a distinct CAF subset, termed glycolytic CAFs (glyCAFs), has been identified in STSs. These cells depend on GLUT1‐mediated expression of CXCL16 to restrict cytotoxic T cell infiltration into the tumor parenchyma. Targeting glycolytic metabolism reduces glyCAF accumulation at the tumor margins, thereby enhancing T cell infiltration and potentiating chemotherapy efficacy [520]. Collectively, CAFs are increasingly recognized as central drivers of tumor progression and immune evasion in STSs. Their multifaceted roles—in promoting proliferation, remodeling the ECM, shaping immune infiltration, and mediating therapeutic resistance—highlight CAFs as not only prognostic indicators but also as one of the most promising and rapidly emerging research frontiers in STS immunobiology.

### 5.5 Other Tumors

In addition to the malignancies discussed above, other musculoskeletal tumors also pose significant challenges in orthopedic oncology. Owing to their relative rarity and limited research attention, these tumors remain largely understudied. Although several studies have begun to characterize their immune landscapes, the underlying mechanisms remain poorly defined.

For instance, in chondroblastoma, increased infiltration of TAMs correlates with enhanced tumor proliferation, local invasion, and suboptimal response to adjuvant radiotherapy [521]. In diffuse‐type tenosynovial giant cell tumors, clinical benefit has been achieved through the depletion of infiltrating TAMs by CSF1R inhibition, albeit with adverse effects such as facial edema [522].

Collectively, these findings highlight a recurring theme: the TME is characterized by profound immune suppression, with TAMs, Tregs, and other immunoregulatory components contributing to disease progression, metastasis, and treatment resistance. Despite significant advances in understanding the genomic and cellular features of bone tumors, the immune landscape remains incompletely defined and therapeutically underexplored. Future efforts to dissect and therapeutically reprogram this landscape will be critical to realizing the potential of immunotherapy in bone oncology.

## 6 Therapeutic Advances for MSDs Targeting the Immune System

Modulation of the immune system has emerged as a central therapeutic paradigm in MSDs. This chapter catalogs a spectrum of immune‐targeted therapies, from molecular and pathway‐specific inhibitors to innovative cell‐based and delivery platforms, and critically evaluate their clinical translation prospects and ongoing challenges.

### 6.1 Therapeutic Strategies Against Molecular Targets

#### 6.1.1 Inflammation‐Modulatory Cytokine Targets

Targeting inflammation‐modulatory cytokines—both proinflammatory and immunosuppressive—has yielded major therapeutic breakthroughs in immune‐related MSDs. For example, TNFα inhibitors are widely used in RA, effectively reducing joint inflammation and bone erosion [523, 524]. An integrated analysis of three clinical trials demonstrated that ozoralizumab, an anti‐TNF nanobody, consistently maintained ACR20 response rates over a mean treatment duration of 200 weeks without introducing new safety concerns in RA patients [525]. Dazukibart, a monoclonal antibody specifically targeting IFNβ, effectively reduced disease activity in adults with DM and was generally well tolerated. In addition, ILs constitute another critical group of cytokines that profoundly shape the immune landscape of MSDs. In a phase III trial, olokizumab—an anti‐IL‐6 monoclonal antibody—significantly improved ACR20 response rates at 12 weeks in RA patients with an inadequate response to methotrexate and demonstrated noninferiority to adalimumab [526]. Long‐term data from the EXTEND study further confirmed the sustained efficacy of sarilumab (anti‐IL‐6R) over 5 years, with manageable neutropenia (15.3 events per 100 patient‐years) in patients refractory to TNF inhibitor therapy [527]. Moreover, ABT‐122, a dual TNF/IL‐17A inhibitor, demonstrated favorable safety and reductions in inflammatory biomarkers in a phase I trial involving RA patients on stable methotrexate treatment [528]. Conversely, anti‐inflammatory ILs such as IL‐10 are also being explored for their regenerative and immunomodulatory potential. A preclinical study using a fusion protein of IL‐10 and albumin (SA‐IL‐10) demonstrated enhanced lymph node targeting and joint protection comparable to anti‐TNF therapy, with more flexible administration routes, in murine RA models [529]. These findings highlight the clinical potential of cytokine modulation in restoring immune balance and preserving skeletal integrity in immune‐dysregulated MSDs.

#### 6.1.2 Bone‐Regulatory Targets

Directly targeting bone‐regulatory mechanisms—particularly through therapeutic strategies aimed at the RANK/RANKL/OPG axis—is critical for preventing structural damage in MSDs. RANKL serves as a key regulator of osteoclast differentiation and bone resorption. Monoclonal antibodies targeting RANKL have demonstrated efficacy in reducing radiographic progression and cortical bone erosion in RA, as well as preventing bone loss in osteoporosis. For example, the DESIRABLE trial showed that denosumab significantly reduced erosion scores and inhibited joint space narrowing in RA patients with an inadequate response to methotrexate, compared with continued methotrexate alone [530]. Additionally, a phase II trial showed that narlumosbart significantly increased lumbar spine bone mineral density at 12 months in postmenopausal women with osteoporosis, compared with placebo [531].

In addition to symptom‐relieving strategies, cartilage‐regenerative agents targeting growth factor pathways have shown promising progress. Sprifermin, a recombinant human fibroblast growth factor 18, has demonstrated the ability to promote cartilage thickening and provide sustained structural benefits over placebo for up to 5 years in patients with knee OA, as shown in the FORWARD trial, with sustained pain improvements observed in patients at high risk of disease progression [532]. These bone‐targeted therapies offer a valuable complement to conventional immunomodulatory treatments, contributing to a more comprehensive management paradigm in MSDs.

#### 6.1.3 Immune Checkpoint and Costimulatory Targets

Immune checkpoint and costimulatory targets—particularly the PD‐1/PD‐L1 and CD28‐CD80/86 pathways—represent transformative therapeutic avenues for recalcitrant MSDs by restoring immune tolerance. Abatacept, a CTLA‐4‐Ig fusion protein, competitively inhibits CD28 binding to CD80/CD86 on APCs, thereby blocking T cell costimulation [533, 534]. For instance, the phase IIb APIPPRA trial demonstrated that abatacept significantly reduced progression to RA at 12 months compared with placebo in ACPA/RF‐positive individuals at high risk of disease onset [535]. Similarly, the phase II ARIAA trial showed that abatacept significantly improved subclinical MRI‐detected inflammation at 6 months in ACPA‐positive individuals presenting with arthralgia and subclinical MRI‐detected synovitis [536].

Beyond costimulation blockade, modulation of the PD‐1/PD‐L1 immune checkpoint pathway has shown emerging therapeutic promise. A phase IIa trial showed that peresolimab, an agonistic PD‐1 antibody, significantly improved DAS28‐CRP scores at 12 weeks compared with placebo in adults with moderate‐to‐severe RA who had an inadequate response to disease‐modifying antirheumatic drugs (DMARDs) [537]. Conversely, inhibitory PD‐1 antibodies have also been explored in musculoskeletal tumors. A phase II trial showed that camrelizumab, combined with standard neoadjuvant chemotherapy, was safe and tolerable in patients with resectable osteosarcoma, with 48.4% achieving a good tumor necrosis response, and an estimated 2‐year PFS and OS rates of 69.6 and 89.4%, respectively, although the impact on tumor necrosis rate requires further validation [538]. In contrast, a separate phase II trial evaluating pembrolizumab in adults with advanced osteosarcoma showed no meaningful clinical benefit, with a median PFS of 1.7 months and OS of 6.6 months, respectively, suggesting that future studies should prioritize biomarker‐guided combination strategies [539]. These strategies collectively exemplify the potential of precision immunomodulation to preserve joint integrity and achieve tumor control while minimizing collateral immune perturbation.

### 6.2 Therapeutic Strategies Against Signaling Pathways

#### 6.2.1 JAK/STAT Signaling Pathway

Targeting the JAK/STAT signaling cascade with selective small‐molecule inhibitors has revolutionized treatment of immune‐driven MSDs. By blocking JAK1/2/3 activity, these agents effectively suppress proinflammatory cytokine signaling (e.g., IL‑6, IFNγ), thereby reducing synovitis, joint damage, and systemic inflammation in conditions such as RA [540]. For example, a phase III trial showed that ivarmacitinib (4 or 8 mg once daily) significantly improved ACR20 response rates at 24 weeks compared with placebo in patients with moderate‐to‐severe RA who had an inadequate response to conventional synthetic DMARDs, with sustained efficacy and a generally manageable safety profile over 52 weeks [541]. Similarly, a phase II trial showed that ritlecitinib (200 mg once daily) significantly improved SDAI scores at 8 weeks versus placebo in adults with RA unresponsive to methotrexate, with a favorable safety profile [542]. Another phase III trial showed that filgotinib (100 or 200 mg once daily) significantly improved ACR20 responses at 12 weeks compared with placebo in patients with moderate‐to‐severe RA who had failed or were intolerant to one or more biologic DMARDs, including those previously treated with ≥3 biologic DMARDs [543]. However, long‑term safety surveillance remains essential, as postmarketing data have flagged potential risks of cardiovascular events and malignancies associated with this therapeutic class.

#### 6.2.2 Wnt/β‐Catenin Signaling Pathway

The Wnt/β‐catenin signaling pathway plays a central role in bone formation and remodeling. Inhibition of endogenous Wnt antagonists, such as Sost, enhances osteoblast activity, promotes bone formation, and increases bone mineral density. Several therapeutic agents targeting the Wnt pathway have progressed into clinical development. For instance, a phase IIb trial showed that lorecivivint, a Wnt pathway modulator, significantly improved pain and function in patients with moderate‐to‐severe knee OA compared with placebo, identifying a 0.07 mg single intra‐articular injection as the optimal effective dose for further evaluation [544]. Moreover, in the context of osteoporosis, anabolic agents targeting the Wnt pathway have shown promising efficacy. Romosozumab, an inhibitory antibody against Sost, has been shown to significantly increase bone formation and provide superior fracture risk reduction compared with alendronate in postmenopausal women [545, 546]. A recent clinical study further demonstrated that romosozumab treatment for 2 and 12 months significantly increased bone formation and improved microstructural remodeling parameters in postmenopausal women with osteoporosis, supporting its dual effect on bone formation and resorption at the tissue level [547].

#### 6.2.3 Isocitrate Dehydrogenase 1 Pathway

Isocitrate dehydrogenase 1 (IDH1) mutations, which lead to the accumulation of the oncometabolite 2‐hydroxyglutarate (2‐HG), are frequently observed in chondrosarcoma, particularly in the conventional (diffuse‐type) subtype. Ivosidenib (AG‐120), an oral selective inhibitor of mutant IDH1, has shown promise in targeting this molecular alteration. A phase I/expansion study conducted in 2020 enrolled 21 patients with advanced chondrosarcoma harboring IDH1 mutations. Treatment with ivosidenib demonstrated a favorable safety profile, with most adverse events being grade 1–2, and resulted in marked reductions in plasma 2‐HG levels. The median PFS was 5.6 months, with a 6‐month PFS rate of 39.5%, and 52% of patients achieved stable disease, durable disease control in this molecularly defined subgroup [548]. Furthermore, a recent phase I trial evaluating ivosidenib at 500 mg daily reported a 23.1% objective response rate and a median PFS of 7.4 months in patients with conventional chondrosarcoma, with manageable toxicity, further supporting its ongoing investigation as a targeted therapy in this setting [451] (Table 1).

**TABLE 1:** Immune‐modulatory targeted therapies for musculoskeletal diseases.

Classification of targets	Registration number	Trail phase	Objective	Compounds and administration route	Dose administered	Condition or disease	Outcome	References
Inflammation‐modulatory cytokine targets	jRCT2080223971, jRCT2080223973, NCT04077567	Phase 2/3	To evaluate long‐term safety and efficacy of ozoralizumab in RA	Ozoralizumab (anti‐TNF), SC injection	30 or 80 mg every 4 weeks	RA	Maintained ACR20 response rates	[525]
	NCT02760407	Phase 3	To assess the efficacy and safety of olokizumab vs placebo and adalimumab in RA	Olokizumab (anti‐IL‐6) or adalimumab (anti‐TNF), SC injection	64 mg every 2 or 4 weeks (olokizumab); 40 mg every 2 weeks (adalimumab)	RA	Increased ACR20 response rates at 12 weeks	[526]
	NCT03181893	Phase 2	To assess the efficacy, safety, and target engagement of dazukibart in moderate‐to‐severe adult DM	Dazukibart (anti‐IFNβ), i.v.	600 or 150 mg every 4 weeks for 8 weeks	DM	Reduced CDASI‐A scores	[549]
	NCT01709578, NCT01146652	Phase 3	To evaluate the long‐term safety and efficacy of sarilumab in RA refractory to TNF inhibitors	Sarilumab (anti‐IL‐6R)	150 or 200 mg every 2 weeks	RA	Maintained efficacy for 5 years	[527]
	NCT02141997	Phase 2	To evaluate the safety and efficacy of ABT‐122 in RA with inadequate responses to MTX	ABT‐122 (anti‐TNF/IL‐17A) or adalimumab (anti‐TNF), SC injection	60 mg every other week, 120 mg every other week, or 120 mg every week (ABT‐122); 40 mg every other week (adalimumab)	RA	Increased ACR20, ACR50, and ACR70 response rates	[528]
Bone‐regulatory targets	Not mentioned	Phase 3	To evaluate the efficacy of denosumab added to conventional synthetic DMARD therapy in RA	Denosumab (anti‐RANKL), SC injection	60 mg every 3 months or 60 mg every 6 months	RA	Reduced erosion scores and inhibited joint space narrowing	[530]
	NCT05278338	Phase 2	To explore the efficacy and safety of narlumosbart in postmenopausal osteoporosis	Narlumosbart (anti‐RANKL), SC injection	45, 60, or 90 mg once every 6 months over a 12‐month period	Osteoporosis	Increased lumbar spine bone mineral density	[531]
	NCT01919164	Phase 2	To assess the long‐term efficacy and safety of intra‐articular sprifermin in knee OA	Sprifermin (rhFGF‐18), intra‐articular injection	100 or 30 µg every 6 months or 12 months	Knee OA	Sustained cartilage thickness benefit and pain improving	[532]
Immune checkpoint and costimulatory targets	EudraCT 2013‐003413‐18	Phase 2b	To evaluate the feasibility, efficacy, and acceptability of abatacept in individuals at high risk of RA	Abatacept (targeting CD80/CD86), SC injection	125 mg weekly for 12 months	RA	Reduced progression to rheumatoid arthritis	[535]
	EudraCT 2014‐000555‐93	Phase 3	To assess whether 6‐month treatment with abatacept improves inflammation in preclinical RA	Abatacept (targeting CD80/CD86), SC injection	125 mg for 6 months	RA	Showed improvement in MRI subclinical inflammation	[536]
	NCT04634253	Phase 2	To evaluate the efficacy and safety of peresolimab in moderate‐to‐severe RA	Peresolimab (stimulatory PD‐1 antibody), i.v.	700 or 300 mg once every 4 weeks	RA	Improved DAS28‐CRP at 12 weeks	[537]
	NCT04294511	Phase 2	To investigate safety and activity of neoadjuvant chemotherapy with camrelizumab in resectable OS	Camrelizumab (anti‐PD‐1) combined with neoadjuvant chemotherapy	200 mg or 3 mg/kg if the body weight ≤40 kg, Day 1, 22, 43 (camrelizumab)	Osteosarcoma	48.4% achieved good tumor necrosis response; estimated 2‐year PFS and OS rates of 69.6 and 89.4%, respectively	[538]
	NCT03013127	Phase 2	To evaluate the activity and safety of pembrolizumab in advanced adult osteosarcoma	Pembrolizumab (anti‐PD‐1), i.v.	200 mg every 21 days	Osteosarcoma	Well‐tolerated but did not show clinically significant antitumor activity	[539]
JAK/STAT signaling pathway	NCT04333771	Phase 3	To assess the efficacy/safety of ivarmacitinib in moderate‐to‐severe active RA with inadequate response to conventional synthetic DMARDs	Ivarmacitinib (JAK1 inhibitor), p.o.	4 or 8 mg once daily	RA	Improved ACR20 response rates at 24 weeks	[541]
	NCT02969044	Phase 2	To evaluate the efficacy and safety of ritlecitinib) in RA	Ritlecitinib (JAK3 inhibitor), p.o.	200 mg once daily for 8 weeks	RA	Improved SDAI scores	[542]
	NCT02873936	Phase 3	To evaluate the effects of filgotinib on the signs and symptoms of refractory RA	Filgotinib (JAK1 inhibitor), p.o.	100 or 200 mg once daily for 24 weeks	RA	Improved ACR20 response at 12 weeks	[543]
Wnt/β‐catenin signaling pathway	NCT03122860	Phase 2b	To identify effective lorecivivint doses in moderate‐to‐severe knee OA	Lorecivivint intra‐articular injection	2 mL (0.03, 0.07, 0.15, or 0.23 mg)	Knee OA	Improved pain and function scores	[544]
	NCT01631214	Phase 3	To compare the efficacy and safety of romosozumab‐to‐alendronate vs. alendronate alone in reducing fracture risk in postmenopausal osteoporosis	Romosozumab (sclerostin inhibitor), SC injection	210 mg weekly for 12 months	Osteoporosis	Reduced fracture risk	[546]
	NCT01575834	Phase 3	To investigate the effects of romosozumab on bone balance and remodeling dynamics at the tissue level in postmenopausal osteoporosis	Romosozumab (sclerostin inhibitor), SC injection	210 mg monthly for 12 months	Osteoporosis	Increased bone formation and improved microstructural remodeling	[547]
IDH1 pathway	NCT02073994	Phase 1	To evaluate the safety and efficacy of ivosidenib in advanced IDH1‐mutant chondrosarcoma	Ivosidenib (mutant IDH1 inhibitor), p.o.	100 mg twice daily to 1,200 mg once daily in continuous 28‐day cycles	Chondrosarcoma	Decrease in plasma 2‐HG levels	[548]
	NCT06127407	Phase 1	To evaluate the safety and efficacy of ivosidenib in advanced IDH1‐mutant chondrosarcoma	Ivosidenib (mutant IDH1 inhibitor), p.o.	100 mg twice daily to 1,200 mg once daily in continuous 28‐day cycles	Chondrosarcoma	23.1% objective response rate and a median PFS of 7.4 months	[451]

*Abbreviations*: 2‐HG, 2‐hydroxyglutarate; ACR, American College of Rheumatology; DAS28‐CRP, disease activity score‐28 based on C‐reactive protein; DM, dermatomyositis; DMARD, disease‐modifying antirheumatic drug, OA, osteoarthritis; i.v., intravenous; IDH1, isocitrate dehydrogenase 1; IFNβ, interferon‐beta; JAK, Janus kinase; MTX, methotrexate; OS, overall survival; p.o., per os (oral administration); PD‐1, programmed cell death protein‐1; PFS, progression‐free survival; RA, rheumatoid arthritis; rhFGF18, recombinant human fibroblast growth factor 18; SC, subcutaneous; SDAI, Simplified Disease Activity Index; TNF, tumor necrosis factor; Wnt, wingless/integrated.

### 6.3 Novel and Emerging Therapies Under Investigation

#### 6.3.1 Cell‐Based Therapies

Cell‐based therapies are emerging as innovative strategies for the treatment of MSDs. Among them, MSC transplantation has shown substantial promise in promoting bone regeneration and modulating inflammatory responses. For example, a randomized, double‐blind clinical study showed that human umbilical cord MSC‐derived exosomes significantly reduced inflammation and promoted cartilage regeneration in patients with OA, showing both clinical and MRI evidence of safety and preliminary efficacy [550]. Moreover, engineered immune‐cell‐based therapies—such as CAR‐T cells—are being actively explored to target tumor‐induced bone destruction. A preclinical study showed that CAR‐T cells targeting ALPL‐1, a tumor‐specific isoform of alkaline phosphatase highly expressed in osteosarcoma, significantly suppressed tumor growth and exhibited potent, selective cytotoxicity against ALPL‐1‐expressing cells. This approach underscores the potential of CAR‐T therapy as a novel and effective approach for treating bone malignancies, offering a targeted strategy to eliminate tumor cells while sparing healthy tissue [551]. Additionally, CD19‐directed CAR‐T cells have shown efficacy in managing refractory JDM, achieving sustained B cell depletion and long‐term clinical and radiologic improvement without ongoing immunosuppressive therapy [202]. In summary, cell‐based therapies represent versatile and promising platforms that integrate regenerative and immunomodulatory functions for MSD treatment.

#### 6.3.2 Advanced Drug Delivery Systems

Advanced drug delivery systems have shown promise in the precision treatment of MSDs. Among these, nanoparticle‐mediated targeted delivery stands out as a particularly effective approach. For example, the Combo‐NPs@shGNE system uses liposomes modified with TAT peptides to codeliver Andrographolide, Icariside II, and shRNA targeting the sialic acid biosynthesis enzyme GNE. In osteosarcoma murine models, this system achieved a 56.44% release rate of tumor‐derived extracellular vesicles and enhanced immune responses by activating DCs, boosting T cell infiltration, reducing MDSCs, and shifting TAMs from the M2 to M1 phenotype [552]. Controlled‐release platforms have also gained increasing attention as complementary approaches. For instance, a pH‐responsive zein–poly(l‐lysine) dendron nanocarrier was developed for the codelivery of siRNA against astrocyte elevated gene‐1 (AEG‐1) and doxorubicin, achieving targeted gene silencing and controlled chemotherapeutic release with minimal cytotoxicity in osteosarcoma models [553]. These innovative delivery technologies offer distinct advantages in overcoming biological barriers, improving drug localization, and integrating therapeutic and immunomodulatory effects for more effective osteosarcoma management.

#### 6.3.3 Others

Novel therapeutic strategies for MSDs are increasingly focusing on the precision modulation of microbiome, metabolism, and RNA‐based therapies. Microbiome‑targeted interventions have been shown to influence osteoimmune balance. For example, oral administration of *Lactobacillus rhamnosus* GG significantly improved estrogen deficiency–induced osteoporosis in OVX rats by modulating the gut–bone axis [554]. In the field of nutrition, a randomized clinical trial demonstrated that daily supplementation with black barberry extract (1000 mg/day) for 12 weeks significantly reduced disease severity and regulated levels of key cytokines—by decreasing IL‐17 and increasing IL‐10—in women with active RA [555]. In addition, RNA‐based therapies have shown promise in overcoming immune resistance in bone‐associated tumors. Systemic delivery of RNA‐loaded lipid particles has been shown to enhance early type‐I interferon responses, promote epitope spreading, and sensitize immunotherapy‐resistant tumors to checkpoint blockade. This strategy may provide an effective approach to overcoming the immunosuppressive TME and enhancing responsiveness to immune checkpoint inhibitors [556].

### 6.4 Translational Perspectives and Clinical Challenges

Translational advances in the field of MSDs hold significant promise, yet they remain accompanied by substantial clinical challenges. Many emerging therapies—such as RNA‐based therapeutics, cell‐based therapies, and molecularly targeted treatments—remain largely confined to the preclinical stages, with limited success in clinical translation. For instance, siRNA‐loaded nanocarriers targeting oncogenes like AEG‐1 have demonstrated potent tumor‐suppressive effects in osteosarcoma animal models; however, concerns including off‐target toxicity and unintended immune activation continue to hinder their clinical application [553]. Similarly, the PI3K/mTOR inhibitor samotolisib demonstrated promising target‐specific efficacy in preclinical models of pediatric osteosarcoma and glioma with pathway alterations, but failed to elicit meaningful clinical responses in a phase II trial—highlighting the ongoing gap between molecular target validation and therapeutic efficacy in patients [557]. Looking ahead, integrating personalized treatment strategies will be critical for overcoming these translational barriers. To improve clinical applicability, future research must prioritize biomarker‐guided patient selection, scalable and reproducible manufacturing processes, and adaptive clinical trial designs that more effectively bridge the divide between bench and bedside.

## 7 Conclusion and Perspectives

MSDs encompass a broad spectrum of inflammatory, degenerative, and neoplastic conditions. Dysregulation of the immune system plays a pivotal role in their pathogenesis by driving aberrant bone and muscle metabolism, persistent inflammation, and immune evasion. At the current stage, several key scientific questions continue to drive this rapidly evolving field. These include elucidating the molecular mechanisms that orchestrate the immune–bone, immune–muscle, and immune–tendon crosstalk; defining the phenotypic and functional heterogeneity of immune cell subsets; and developing strategies that exploit immune cell plasticity to balance immune modulation and tissue regeneration. In this review, we systematically summarized the intricate crosstalk between immune cells and tissue cells within the physiological musculoskeletal microenvironment, followed by a comprehensive discussion of the immunological mechanisms underlying representative MSDs and recent advances in immunomodulatory therapies. Current research hotspots—such as single‐cell and spatial omics of the musculoskeletal immune microenvironment, macrophage polarization and plasticity, and the role of systemic senescence—are also highlighted.

Importantly, the dynamic and often paradoxical roles of immune cells add significant complexity to the immunological landscape of MSDs. Beyond the well‐characterized polarization states of macrophages, other examples highlight this duality: activated antigen‐presenting DCs coexist with tumor‐protective mregDCs in osteosarcoma [408], while classical Tregs with immunosuppressive functions are accompanied by noncanonical osteoclastogenic Th17‐like Tregs in severe PsA [170]. Moreover, immune microenvironments traditionally considered “immune‐overloaded,” as seen in inflammatory bone and muscle diseases, also harbor regulatory elements; conversely, the “immune‐excluded” TME of bone tumors can also harbor functionally active mediators that sustain residual immune surveillance. These observations underscore the necessity of accurately identifying functional immune cell subsets and states, as their plasticity and context‐dependent behavior critically influence disease outcomes and therapeutic responses.

As research advances, multidisciplinary approaches integrating immunology, molecular biology, and musculoskeletal metabolism are driving the field forward. Emerging strategies—such as nanocarrier‐based delivery of immunotherapeutics [558], exosome‐mediated immune modulation [559], and adoptive cell therapies—offer promising avenues for treating bone‐related disorders. Nevertheless, several critical challenges remain unresolved. First, it remains uncertain whether data derived from in vitro and murine models faithfully recapitulate the human immune–bone interface, particularly in chronic degenerative or malignant contexts. More physiologically relevant models, such as patient‐derived organoids that preserve the native immune environment, may yield more reliable insights. Second, the immense diversity of immune cell subsets necessitates more nuanced and standardized markers for subclassification. Yet, inconsistent criteria across studies hinder reproducibility and integrative understanding. Underexplored populations—such as Bregs, γδT cells, and ILCs—deserve greater attention in future investigations. Third, due to broad spectrum of MSDs, clinically significant conditions such as traumatic fractures remain insufficiently investigated from an immunological perspective. Furthermore, the immunological interplay becomes even more complex when inflammatory and neoplastic conditions coexist. For instance, adolescent osteosarcoma and Ewing sarcoma patients undergoing chemotherapy often exhibit increased susceptibility to osteoporosis [560, 561], and chronic localized infections have been associated with improved survival in osteosarcoma patients [562]—raising the intriguing possibility that seemingly adverse immune activation may exert protective effects in certain contexts, a hypothesis warranting deeper investigation.

In conclusion, decoding the immunological mechanisms driving MSDs not only enhances our understanding of their pathogenesis but also paves the way for novel therapeutic interventions. Looking ahead, future studies should prioritize advanced methodologies such as patient‐derived organoid models, high‐dimensional immunoprofiling, and real‐time in vivo imaging to better capture dynamic immune–bone interactions. Ultimately, bridging the gap between mechanistic discoveries and clinical translation will be essential for developing precise, durable, and tissue‐specific therapies tailored to the complexity and heterogeneity of MSDs.

## Author Contributions

XM, EY, and XC contributed equally to this work. NL, XC, and FW conceived and designed the study. XM, EY, and ZC drafted the manuscript and prepared the figures. SW, HZ, and YX revised the manuscript. JL, FC, and HL critically revised the manuscript, contributed to language polishing and logical refinement of the manuscript. SC, SZ, KW, and LC cross‐checked the literature sources, validated the accuracy of key references, and provided vital comments to the manuscript. JG, ZY, and NL supervised the study. NL acquired the funding. All authors have read and approved the final manuscript.

## Funding Information

This study was supported by the Key R&D Program of Zhejiang Province (2022C03105). The funding source did not play a role in the investigation.

## Conflicts of Interest

The authors declare no conflicts of interest.

## Ethics Statement

The authors have nothing to report.

## Data Availability

The authors have nothing to report.

## References

[1] C. Mancino , M. Franke , A. Greco , et al., “RNA Therapies for Musculoskeletal Conditions,” Journal of Control Release 377 (2025): 756–766. 10.1016/j.jconrel.2024.11.057 39617171

[2] D. Goltzman , “Discoveries, Drugs and Skeletal Disorders,” Nature Reviews Drug Discovery 1, no. 10 (2002): 784–796. 12360256 10.1038/nrd916

[3] M. Hayashi , T. Nakashima , M. Taniguchi , T. Kodama , A. Kumanogoh , and H. Takayanagi , “Osteoprotection by Semaphorin 3A,” Nature 485, no. 7396 (2012): 69–74. 22522930 10.1038/nature11000

[4] J. Sieper and D. Poddubnyy , “Axial Spondyloarthritis,” Lancet 390, no. 10089 (2017): 73–84. 28110981 10.1016/S0140-6736(16)31591-4

[5] J. S. Smolen , D. Aletaha , and I. B. McInnes , “Rheumatoid Arthritis,” Lancet 388, no. 10055 (2016): 2023–2038. 27156434 10.1016/S0140-6736(16)30173-8

[6] B. Yu and C. Y. Wang , “Osteoporosis and Periodontal Diseases—An Update on Their Association and Mechanistic Links,” Periodontology 2000 89, no. 1 (2022): 99–113. 35244945 10.1111/prd.12422 PMC9067601

[7] K. Okamoto , T. Nakashima , M. Shinohara , et al., “Osteoimmunology: The Conceptual Framework Unifying the Immune and Skeletal Systems,” Physiological Reviews 97, no. 4 (2017): 1295–1349. 28814613 10.1152/physrev.00036.2016

[8] H. Takayanagi , “Osteoimmunology: Shared Mechanisms and Crosstalk Between the Immune and Bone Systems,” Nature Reviews Immunology 7, no. 4 (2007): 292–304. 10.1038/nri2062 17380158

[9] H. Takayanagi , K. Ogasawara , S. Hida , et al., “T‐cell‐mediated Regulation of Osteoclastogenesis by Signalling Cross‐talk Between RANKL and IFN‐gamma,” Nature 408, no. 6812 (2000): 600–605. 11117749 10.1038/35046102

[10] N. L. Rosenberg , S. P. Ringel , and B. L. Kotzin , “Experimental Autoimmune Myositis in SJL/J Mice,” Clinical and Experimental Immunology 68, no. 1 (1987): 117–129. 3308207 PMC1542699

[11] N. L. Millar , K. G. Silbernagel , K. Thorborg , et al., “Tendinopathy,” Nature Reviews Disease Primers 7, no. 1 (2021): 1. 10.1038/s41572-020-00234-1 33414454

[12] M. N. Weitzmann and I. Ofotokun , “Physiological and Pathophysiological Bone Turnover—role of the Immune System,” Nature Reviews Endocrinology 12, no. 9 (2016): 518–532. 10.1038/nrendo.2016.91 PMC5857945 27312863

[13] S. L. Teitelbaum and F. P. Ross , “Genetic Regulation of Osteoclast Development and Function,” Nature Reviews Genetics 4, no. 8 (2003): 638–649. 10.1038/nrg1122 12897775

[14] Y. Xiong , B. B. Mi , Z. Lin , et al., “The Role of the Immune Microenvironment in Bone, Cartilage, and Soft Tissue Regeneration: From Mechanism to Therapeutic Opportunity,” Military Medical Research 9, no. 1 (2022): 65. 36401295 10.1186/s40779-022-00426-8 PMC9675067

[15] K. Horas , C. Menale , and A. Maurizi , “Editorial: The Bone/Bone Marrow Microenvironment: A Hub for Immune Regulation of the Tumor Cells Fate,” Frontiers in Immunology 13 (2022): 1019489. 36119043 10.3389/fimmu.2022.1019489 PMC9471548

[16] A. G. Robling and L. F. Bonewald , “The Osteocyte: New Insights,” Annual Review of Physiology 82 (2020): 485–506. 10.1146/annurev-physiol-021119-034332 PMC8274561 32040934

[17] Q. Chen , P. Shou , C. Zheng , et al., “Fate Decision of Mesenchymal Stem Cells: Adipocytes or Osteoblasts?,” Cell Death and Differentiation 23, no. 7 (2016): 1128–1139. 26868907 10.1038/cdd.2015.168 PMC4946886

[18] M. Ponzetti and N. Rucci , “Osteoblast Differentiation and Signaling: Established Concepts and Emerging Topics,” International Journal of Molecular Sciences 22, no. 13 (2021): 6651. 34206294 10.3390/ijms22136651 PMC8268587

[19] I. Takada , A. P. Kouzmenko , and S. Kato , “Wnt and PPARgamma Signaling in Osteoblastogenesis and Adipogenesis,” Nature Reviews Rheumatology 5, no. 8 (2009): 442–447. 19581903 10.1038/nrrheum.2009.137

[20] N. Udagawa , M. Koide , M. Nakamura , et al., “Osteoclast Differentiation by RANKL and OPG Signaling Pathways,” Journal of Bone and Mineral Metabolism 39, no. 1 (2021): 19–26. 33079279 10.1007/s00774-020-01162-6

[21] L. de la Rica , A. Garcia‐Gomez , N. R. Comet , et al., “NF‐kappaB‐direct Activation of microRNAs With Repressive Effects on Monocyte‐specific Genes Is Critical for Osteoclast Differentiation,” Genome Biology 16, no. 1 (2015): 2. 25601191 10.1186/s13059-014-0561-5 PMC4290566

[22] R. Leung , K. Cuddy , Y. Wang , J. Rommens , and M. Glogauer , “Sbds Is Required for Rac2‐mediated Monocyte Migration and Signaling Downstream of RANK During Osteoclastogenesis,” Blood 117, no. 6 (2011): 2044–2053. 21084708 10.1182/blood-2010-05-282574

[23] R. J. Miron , M. Bohner , Y. Zhang , and D. D. Bosshardt , “Osteoinduction and Osteoimmunology: Emerging Concepts,” Periodontology 2000 94, no. 1 (2024): 9–26. 37658591 10.1111/prd.12519

[24] Y. Li , G. Toraldo , A. Li , et al., “B Cells and T Cells Are Critical for the Preservation of Bone Homeostasis and Attainment of Peak Bone Mass in Vivo,” Blood 109, no. 9 (2007): 3839–3848. 17202317 10.1182/blood-2006-07-037994 PMC1874582

[25] A. Bozec , M. M. Zaiss , R. Kagwiria , et al., “T Cell Costimulation Molecules CD80/86 Inhibit Osteoclast Differentiation by Inducing the IDO/Tryptophan Pathway,” Science Translational Medicine 6, no. 235 (2014): 235ra60. 10.1126/scitranslmed.3007764 24807557

[26] L. W. Lai , K. C. Yong , and Y. H. Lien , “Pharmacologic Recruitment of Regulatory T Cells as a Therapy for Ischemic Acute Kidney Injury,” Kidney International 81, no. 10 (2012): 983–992. 22189844 10.1038/ki.2011.412 PMC3340526

[27] L. Goschl , C. Scheinecker , and M. Bonelli , “Treg Cells in Autoimmunity: From Identification to Treg‐based Therapies,” Seminars in Immunopathology 41, no. 3 (2019): 301–314. 30953162 10.1007/s00281-019-00741-8

[28] M. Arizon , I. Nudel , H. Segev , et al., “Langerhans Cells Down‐regulate Inflammation‐driven Alveolar Bone Loss,” Proceedings of the National Academy of Sciences of the United States of America 109, no. 18 (2012): 7043–7048. 22509018 10.1073/pnas.1116770109 PMC3344981

[29] A. Bozec and M. M. Zaiss , “T Regulatory Cells in Bone Remodelling,” Current Osteoporosis Reports 15, no. 3 (2017): 121–125. 28432597 10.1007/s11914-017-0356-1

[30] M. Noack and P. Miossec , “Th17 and Regulatory T Cell Balance in Autoimmune and Inflammatory Diseases,” Autoimmunity Reviews 13, no. 6 (2014): 668–677. 24418308 10.1016/j.autrev.2013.12.004

[31] Z. S. Buchwald , J. R. Kiesel , R. DiPaolo , M. S. Pagadala , and R. Aurora , “Osteoclast Activated FoxP3+ CD8+ T‐cells Suppress Bone Resorption in Vitro,” PLoS ONE 7, no. 6 (2012): e38199. 22701612 10.1371/journal.pone.0038199 PMC3368916

[32] L. Jia , Y. Tu , X. Jia , et al., “Probiotics Ameliorate Alveolar Bone Loss by Regulating Gut Microbiota,” Cell Proliferation 54, no. 7 (2021): e13075. 34101283 10.1111/cpr.13075 PMC8249787

[33] L. Gonzalez‐Osuna , A. Sierra‐Cristancho , C. Rojas , et al., “Premature Senescence of T‐cells Favors Bone Loss during Osteolytic Diseases. A New Concern in the Osteoimmunology Arena,” Aging and Disease 12, no. 5 (2021): 1150–1161. 34341698 10.14336/AD.2021.0110 PMC8279535

[34] M. K. Chang , L. J. Raggatt , K. A. Alexander , et al., “Osteal Tissue Macrophages Are Intercalated throughout human and Mouse Bone Lining Tissues and Regulate Osteoblast Function in Vitro and in Vivo,” Journal of Immunology 181, no. 2 (2008): 1232–1244. 10.4049/jimmunol.181.2.1232 18606677

[35] C. J. Li , Y. Xiao , Y. C. Sun , et al., “Senescent Immune Cells Release Grancalcin to Promote Skeletal Aging,” Cell Metabolism 33, no. 10 (2021): 1957–1973. e6. 34614408 10.1016/j.cmet.2021.08.009

[36] C. Schlundt , H. Fischer , C. H. Bucher , C. Rendenbach , G. N. Duda , and K. Schmidt‐Bleek , “The Multifaceted Roles of Macrophages in Bone Regeneration: A Story of Polarization, Activation and Time,” Acta Biomaterialia 133 (2021): 46–57. 33974949 10.1016/j.actbio.2021.04.052

[37] I. G. Winkler , N. A. Sims , A. R. Pettit , et al., “Bone Marrow Macrophages Maintain Hematopoietic Stem Cell (HSC) Niches and Their Depletion Mobilizes HSCs,” Blood 116, no. 23 (2010): 4815–4828. 20713966 10.1182/blood-2009-11-253534

[38] K. L. Spiller , R. R. Anfang , K. J. Spiller , et al., “The Role of Macrophage Phenotype in Vascularization of Tissue Engineering Scaffolds,” Biomaterials 35, no. 15 (2014): 4477–4488. 24589361 10.1016/j.biomaterials.2014.02.012 PMC4000280

[39] X. Cui , L. Xu , Y. Shan , J. Li , J. Ji , and E. Wang , “Piezocatalytically‐induced Controllable Mineralization Scaffold With Bone‐Like Microenvironment to Achieve Endogenous Bone Regeneration,” Science Bulletin (Beijing) 69, no. 12 (2024): 1895–1908. 10.1016/j.scib.2024.04.002 38637224

[40] X. Lin , S. Patil , Y. G. Gao , and A. Qian , “The Bone Extracellular Matrix in Bone Formation and Regeneration,” Frontiers in Pharmacology 11 (2020): 757. 32528290 10.3389/fphar.2020.00757 PMC7264100

[41] M. D. Shoulders and R. T. Raines , “Collagen Structure and Stability,” Annual Review of Biochemistry 78 (2009): 929–958. 10.1146/annurev.biochem.77.032207.120833 PMC2846778 19344236

[42] M. Murshed , “Mechanism of Bone Mineralization,” Cold Spring Harbor Perspectives in Medicine 8, no. 12 (2018): a031229. 29610149 10.1101/cshperspect.a031229 PMC6280711

[43] H. Fonseca , D. Moreira‐Goncalves , H. J. Coriolano , and J. A. Duarte , “Bone Quality: The Determinants of Bone Strength and Fragility,” Sports Medicine (Auckland, NZ) 44, no. 1 (2014): 37–53. 10.1007/s40279-013-0100-7 24092631

[44] A. Persson , M. Nikpour , E. Vorontsov , J. Nilsson , and G. Larson , “Domain Mapping of Chondroitin/Dermatan Sulfate Glycosaminoglycans Enables Structural Characterization of Proteoglycans,” Molecular & Cellular Proteomics 20 (2021): 100074. 33757834 10.1016/j.mcpro.2021.100074 PMC8724862

[45] P. G. Scott , P. A. McEwan , C. M. Dodd , E. M. Bergmann , P. N. Bishop , and J. Bella , “Crystal Structure of the Dimeric Protein Core of Decorin, the Archetypal Small Leucine‐rich Repeat Proteoglycan,” Proceedings of the National Academy of Sciences of the United States of America 101, no. 44 (2004): 15633–15638. 15501918 10.1073/pnas.0402976101 PMC524833

[46] A. Sorvina , M. Antoniou , Z. Esmaeili , and M. Kochetkova , “Unusual Suspects: Bone and Cartilage ECM Proteins as Carcinoma Facilitators,” Cancers (Basel) 15, no. 3 (2023): 791. 36765749 10.3390/cancers15030791 PMC9913341

[47] M. B. Chavez , M. H. Tan , T. N. Kolli , et al., “Bone Sialoprotein Is Critical for Alveolar Bone Healing in Mice,” Journal of Dental Research 102, no. 2 (2023): 187–196. 36377066 10.1177/00220345221126716 PMC9893390

[48] F. F. Mohamed , B. Hoac , A. Phanrungsuwan , et al., “Contributions of Increased Osteopontin and Hypophosphatemia to Dentoalveolar Defects in Osteomalacic Hyp Mice,” Bone 176 (2023): 116886. 37634682 10.1016/j.bone.2023.116886 PMC10529969

[49] B. Kirk , G. Lombardi , and G. Duque , “Bone and Muscle Crosstalk in Ageing and Disease,” Nature Reviews Endocrinology 21, no. 6 (2025): 375–390. 10.1038/s41574-025-01088-x 40011751

[50] Z. Zhang , S. Lin , W. Luo , et al., “Sox6 Differentially Regulates Inherited Myogenic Abilities and Muscle Fiber Types of Satellite Cells Derived From Fast‐ and Slow‐Type Muscles,” International Journal of Molecular Sciences 23, no. 19 (2022): 11327. 36232654 10.3390/ijms231911327 PMC9569562

[51] C. Rodriguez , F. Timoteo‐Ferreira , G. Minchiotti , S. Brunelli , and O. Guardiola , “Cellular Interactions and Microenvironment Dynamics in Skeletal Muscle Regeneration and Disease,” Frontiers in Cell and Developmental Biology 12 (2024): 1385399. 38840849 10.3389/fcell.2024.1385399 PMC11150574

[52] M. R. Hicks and A. D. Pyle , “The Emergence of the Stem Cell Niche,” Trends in Cell Biology 33, no. 2 (2023): 112–123. 35934562 10.1016/j.tcb.2022.07.003 PMC9868094

[53] C. Sciorati , E. Rigamonti , A. A. Manfredi , and P. Rovere‐Querini , “Cell Death, Clearance and Immunity in the Skeletal Muscle,” Cell Death and Differentiation 23, no. 6 (2016): 927–937. 26868912 10.1038/cdd.2015.171 PMC4987728

[54] B. N. VanderVeen , E. A. Murphy , and J. A. Carson , “The Impact of Immune Cells on the Skeletal Muscle Microenvironment during Cancer Cachexia,” Frontiers in Physiology 11 (2020): 1037. 32982782 10.3389/fphys.2020.01037 PMC7489038

[55] Z. You , X. Huang , Y. Xiang , J. Dai , J. Jiang , and J. Xu , “Molecular Feature of Neutrophils in Immune Microenvironment of Muscle Atrophy,” Journal of Cellular and Molecular Medicine 26, no. 17 (2022): 4658–4665. 35899367 10.1111/jcmm.17495 PMC9443939

[56] M. Becker , S. S. Joseph , F. Garcia‐Carrizo , et al., “Regulatory T Cells Require IL6 Receptor Alpha Signaling to Control Skeletal Muscle Function and Regeneration,” Cell Metabolism 35, no. 10 (2023): 1736–1751. e7. 37734370 10.1016/j.cmet.2023.08.010 PMC10563138

[57] R. P. Wohlgemuth , S. Sriram , K. E. Henricson , D. T. Dinh , S. E. Brashear , and L. R. Smith , “Strain‐dependent Dynamic Re‐alignment of Collagen Fibers in Skeletal Muscle Extracellular Matrix,” Acta Biomaterialia 187 (2024): 227–241. 39209134 10.1016/j.actbio.2024.08.035 PMC11804869

[58] R. P. Wohlgemuth , S. E. Brashear , and L. R. Smith , “Alignment, Cross Linking, and Beyond: A Collagen Architect's Guide to the Skeletal Muscle Extracellular Matrix,” American Journal of Physiology. Cell Physiology 325, no. 4 (2023): C1017–C1030. 37661921 10.1152/ajpcell.00287.2023 PMC10635663

[59] X. Ge , Y. Jin , J. He , Z. Jia , Y. Liu , and Y. Xu , “Extracellular Matrix in Skeletal Muscle Injury and Atrophy: Mechanisms and Therapeutic Implications,” Journal of Orthopaedic Translation 52 (2025): 404–418. 40485851 10.1016/j.jot.2025.03.004 PMC12145571

[60] P. Mohassel , H. Hearn , J. Rooney , et al., “Collagen Type VI Regulates TGF‐beta Bioavailability in Skeletal Muscle in Mice,” Journal of Clinical Investigation 135, no. 9 (2025): e173354. 40309777 10.1172/JCI173354 PMC12043086

[61] S. S. Rayagiri , D. Ranaldi , A. Raven , et al., “Basal Lamina Remodeling at the Skeletal Muscle Stem Cell Niche Mediates Stem Cell Self‐renewal,” Nature Communications 9, no. 1 (2018): 1075. 10.1038/s41467-018-03425-3 PMC5852002 29540680

[62] M. Gomez‐Florit , C. J. Labrador‐Rached , R. M. A. Domingues , and M. E. Gomes , “The Tendon Microenvironment: Engineered in Vitro Models to Study Cellular Crosstalk,” Advanced Drug Delivery Reviews 185 (2022): 114299. 35436570 10.1016/j.addr.2022.114299

[63] A. Dede Eren , S. Vermeulen , T. C. Schmitz , J. Foolen , and J. de Boer , “The Loop of Phenotype: Dynamic Reciprocity Links Tenocyte Morphology to Tendon Tissue Homeostasis,” Acta Biomaterialia 163 (2023): 275–286. 35584748 10.1016/j.actbio.2022.05.019

[64] A. Goto , S. Komura , K. Kato , et al., “PI3K‐Akt Signalling Regulates Scx‐lineage Tenocytes and Tppp3‐lineage Paratenon Sheath Cells in Neonatal Tendon Regeneration,” Nature Communications 16, no. 1 (2025): 3734. 10.1038/s41467-025-59010-y PMC12010001 40254618

[65] S. Chen , Y. Lin , H. Yang , et al., “A CD26(+) Tendon Stem Progenitor Cell Population Contributes to Tendon Repair and Heterotopic Ossification,” Nature Communications 16, no. 1 (2025): 749. 10.1038/s41467-025-56112-5 PMC11739514 39820504

[66] V. Russo , M. El Khatib , G. Prencipe , et al., “Tendon Immune Regeneration: Insights on the Synergetic Role of Stem and Immune Cells During Tendon Regeneration,” Cells 11, no. 3 (2022): 434. 35159244 10.3390/cells11030434 PMC8834336

[67] C. A. Bautista , A. Srikumar , E. D. Tichy , et al., “CD206+ tendon Resident Macrophages and Their Potential Crosstalk With Fibroblasts and the ECM During tendon Growth and Maturation,” Frontiers in Physiology 14 (2023): 1122348. 36909235 10.3389/fphys.2023.1122348 PMC9992419

[68] C. Lehner , G. Spitzer , R. Gehwolf , et al., “Tenophages: A Novel Macrophage‐Like Tendon Cell Population Expressing CX3CL1 and CX3CR1,” Disease Models & Mechanisms 12, no. 12 (2019): dmm041384. 31744815 10.1242/dmm.041384 PMC6918766

[69] M. A. Alim , M. Peterson , and G. Pejler , “Do Mast Cells Have a Role in Tendon Healing and Inflammation?,” Cells 9, no. 5 (2020): 1134. 32375419 10.3390/cells9051134 PMC7290807

[70] R. Mousavizadeh , C. M. Waugh , R. G. McCormack , B. E. Cairns , and A. Scott , “MRGPRX2‐mediated Mast Cell Activation by Substance P From Overloaded human Tenocytes Induces Inflammatory and Degenerative Responses in Tendons,” Scientific Reports 14, no. 1 (2024): 13540. 38866832 10.1038/s41598-024-64222-1 PMC11169467

[71] A. C. Noah , T. M. Li , L. M. Martinez , et al., “Adaptive and Innate Immune Cell Responses in Tendons and Lymph Nodes After Tendon Injury and Repair,” Journal of Applied Physiology (1985) 128, no. 3 (2020): 473–482. 10.1152/japplphysiol.00682.2019 PMC7099435 31944888

[72] K. T. Tam and K. Baar , “Using Load to Improve Tendon/Ligament Tissue Engineering and Develop Novel Treatments for Tendinopathy,” Matrix Biology 135 (2025): 39–54. 39645093 10.1016/j.matbio.2024.12.001

[73] M. Stanczak , B. Kacprzak , and P. Gawda , “Tendon Cell Biology: Effect of Mechanical Loading,” Cellular Physiology and Biochemistry 58, no. 6 (2024): 677–701. 39568406 10.33594/000000743

[74] H. Wolfenson , B. Yang , and M. P. Sheetz , “Steps in Mechanotransduction Pathways That Control Cell Morphology,” Annual Review of Physiology 81 (2019): 585–605. 10.1146/annurev-physiol-021317-121245 PMC7476682 30403543

[75] M. Li , T. Deng , Q. Chen , et al., “A Versatile Platform Based on Matrix Metalloproteinase‐sensitive Peptides for Novel Diagnostic and Therapeutic Strategies in Arthritis,” Bioactive Materials 47 (2025): 100–120. 39897588 10.1016/j.bioactmat.2025.01.011 PMC11787566

[76] P. Brown , A. G. Pratt , and K. L. Hyrich , “Therapeutic Advances in Rheumatoid Arthritis,” Bmj 384 (2024): e070856. 38233032 10.1136/bmj-2022-070856

[77] M. Kurowska‐Stolarska and S. Alivernini , “Synovial Tissue Macrophages in Joint Homeostasis, Rheumatoid Arthritis and Disease Remission,” Nature Reviews Rheumatology 18, no. 7 (2022): 384–397. 35672464 10.1038/s41584-022-00790-8

[78] J. S. Smolen , D. Aletaha , M. Koeller , M. H. Weisman , and P. Emery , “New Therapies for Treatment of Rheumatoid Arthritis,” Lancet 370, no. 9602 (2007): 1861–1874. 17570481 10.1016/S0140-6736(07)60784-3

[79] I. A. Udalova , A. Mantovani , and M. Feldmann , “Macrophage Heterogeneity in the Context of Rheumatoid Arthritis,” Nature Reviews Rheumatology 12, no. 8 (2016): 472–485. 27383913 10.1038/nrrheum.2016.91

[80] D. Kuo , J. Ding , I. S. Cohn , et al., “HBEGF(+) Macrophages in Rheumatoid Arthritis Induce Fibroblast Invasiveness,” Science Translational Medicine 11, no. 491 (2019): eaau8587. 31068444 10.1126/scitranslmed.aau8587 PMC6726376

[81] X. Zhang , Z. Zhang , Y. Zhao , L. Jin , Y. Tai , and Y. Tang , “Sodium Chloride Promotes Macrophage Pyroptosis and Aggravates Rheumatoid Arthritis by Activating SGK1 Through GABA Receptors Slc6a12,” International Journal of Biological Sciences 20, no. 8 (2024): 2922–2942. 38904021 10.7150/ijbs.93242 PMC11186373

[82] K. Ley , H. M. Hoffman , P. Kubes , et al., “Neutrophils: New Insights and Open Questions,” Science Immunology 3, no. 30 (2018): eaat4579. 30530726 10.1126/sciimmunol.aat4579

[83] V. Papayannopoulos , “Neutrophil Extracellular Traps in Immunity and Disease,” Nature Reviews Immunology 18, no. 2 (2018): 134–147. 10.1038/nri.2017.105 28990587

[84] L. J. O'Neil and M. J. Kaplan , “Neutrophils in Rheumatoid Arthritis: Breaking Immune Tolerance and Fueling Disease,” Trends in Molecular Medicine 25, no. 3 (2019): 215–227. 30709614 10.1016/j.molmed.2018.12.008

[85] F. Wu , J. Gao , J. Kang , et al., “B Cells in Rheumatoid Arthritis:Pathogenic Mechanisms and Treatment Prospects,” Frontiers in Immunology 12 (2021): 750753. 34650569 10.3389/fimmu.2021.750753 PMC8505880

[86] S. K. O'Neill , M. J. Shlomchik , T. T. Glant , Y. Cao , P. D. Doodes , and A. Finnegan , “Antigen‐specific B Cells Are Required as APCs and Autoantibody‐producing Cells for Induction of Severe Autoimmune Arthritis,” Journal of Immunology 174, no. 6 (2005): 3781–3788. 10.4049/jimmunol.174.6.3781 15749919

[87] W. Sun , N. Meednu , A. Rosenberg , et al., “B Cells Inhibit Bone Formation in Rheumatoid Arthritis by Suppressing Osteoblast Differentiation,” Nature Communications 9, no. 1 (2018): 5127. 10.1038/s41467-018-07626-8 PMC6277442 30510188

[88] L. Laurent , F. Anquetil , C. Clavel , et al., “IgM Rheumatoid Factor Amplifies the Inflammatory Response of Macrophages Induced by the Rheumatoid Arthritis‐specific Immune Complexes Containing Anticitrullinated Protein Antibodies,” Annals of the Rheumatic Diseases 74, no. 7 (2015): 1425–1431. 24618262 10.1136/annrheumdis-2013-204543

[89] Y. Y. Kong , U. Feige , I. Sarosi , et al., “Activated T Cells Regulate Bone Loss and Joint Destruction in Adjuvant Arthritis Through Osteoprotegerin Ligand,” Nature 402, no. 6759 (1999): 304–309. 10580503 10.1038/46303

[90] H. Ye , D. Fu , X. Fang , Y. Xie , X. Zheng , and W. Fan , “Casein Kinase II Exacerbates Rheumatoid Arthritis via Promoting Th1 and Th17 Cell Inflammatory Responses,” Expert Opinion on Therapeutic Targets 25, no. 11 (2021): 1017–1024. 34806506 10.1080/14728222.2021.2010190

[91] N. Komatsu and H. Takayanagi , “Mechanisms of Joint Destruction in Rheumatoid Arthritis—immune Cell‐fibroblast‐bone Interactions,” Nature Reviews Rheumatology 18, no. 7 (2022): 415–429. 35705856 10.1038/s41584-022-00793-5

[92] A. T. Shaw , Y. Maeda , and E. M. Gravallese , “IL‐17A Deficiency Promotes Periosteal Bone Formation in a Model of Inflammatory Arthritis,” Arthritis Research & Therapy 18, no. 1 (2016): 104. 27165410 10.1186/s13075-016-0998-x PMC4863346

[93] T. Ono , K. Okamoto , T. Nakashima , et al., “IL‐17‐producing Gammadelta T Cells Enhance Bone Regeneration,” Nature Communications 7 (2016): 10928. 10.1038/ncomms10928 PMC4792964 26965320

[94] B. Li , R. Su , Q. Guo , et al., “Differential Immunological Profiles in Seronegative versus Seropositive Rheumatoid Arthritis: Th17/Treg Dysregulation and IL‐4,” Frontiers in Immunology 15 (2024): 1447213. 39290695 10.3389/fimmu.2024.1447213 PMC11405332

[95] J. Iske , Y. Cao , M. J. Roesel , Z. Shen , and Y. Nian , “Metabolic Reprogramming of Myeloid‐derived Suppressor Cells in the Context of Organ Transplantation,” Cytotherapy 25, no. 8 (2023): 789–797. 37204374 10.1016/j.jcyt.2023.04.010

[96] Q. Jiang , J. Duan , L. V. Kaer , and G. Yang , “The Role of Myeloid‐Derived Suppressor Cells in Multiple Sclerosis and Its Animal Model,” Aging and Disease 15, no. 3 (2023): 1329–1343. 10.14336/AD.2023.0323-1 PMC11081146 37307825

[97] M. Y. Wu , C. J. Li , G. T. Yiang , et al., “Molecular Regulation of Bone Metastasis Pathogenesis,” Cellular Physiology and Biochemistry 46, no. 4 (2018): 1423–1438. 29689559 10.1159/000489184

[98] A. Glover , Z. Zhang , and C. Shannon‐Lowe , “Deciphering the Roles of Myeloid Derived Suppressor Cells in Viral Oncogenesis,” Frontiers in Immunology 14 (2023): 1161848. 37033972 10.3389/fimmu.2023.1161848 PMC10076641

[99] E. Y. So , C. Sun , K. Q. Wu , P. M. Dubielecka , A. M. Reginato , and O. D. Liang , “Inhibition of Lipid Phosphatase SHIP1 Expands Myeloid‐derived Suppressor Cells and Attenuates Rheumatoid Arthritis in Mice,” American Journal of Physiology. Cell Physiology 321, no. 3 (2021): C569–C584. 34288720 10.1152/ajpcell.00433.2020 PMC8461811

[100] M. Xue , H. Lin , H. P. H. Liang , et al., “Deficiency of Protease‐activated Receptor (PAR) 1 and PAR2 Exacerbates Collagen‐induced Arthritis in Mice via Differing Mechanisms,” Rheumatology 60, no. 6 (2021): 2990–3003. 33823532 10.1093/rheumatology/keaa701

[101] J. F. Charles , L. Y. Hsu , E. C. Niemi , A. Weiss , A. O. Aliprantis , and M. C. Nakamura , “Inflammatory Arthritis Increases Mouse Osteoclast Precursors With Myeloid Suppressor Function,” Journal of Clinical Investigation 122, no. 12 (2012): 4592–4605. 23114597 10.1172/JCI60920 PMC3533532

[102] M. Li , Z. Tang , R. Shu , et al., “Polymorphonuclear Myeloid‐derived Suppressor Cells Play a Proinflammatory Role via TNF‐alpha(+) B Cells Through BAFF/BTK/NF‐kappaB Signalling Pathway in the Pathogenesis of Collagen‐induced Arthritis Mice,” Immunology 170, no. 2 (2023): 286–300. 37337447 10.1111/imm.13668

[103] L. Liu , D. Deng , C. Li , et al., “The Combination of Modified Acupuncture Needle and Melittin Hydrogel as a Novel Therapeutic Approach for Rheumatoid Arthritis Treatment,” Journal of Nanobiotechnology 22, no. 1 (2024): 432. 39034393 10.1186/s12951-024-02722-y PMC11265141

[104] P. Shen , Y. Jiao , L. Miao , J. H. Chen , and A. A. Momtazi‐Borojeni , “Immunomodulatory Effects of berberine on the Inflamed Joint Reveal New Therapeutic Targets for Rheumatoid Arthritis Management,” Journal of Cellular and Molecular Medicine 24, no. 21 (2020): 12234–12245. 32969153 10.1111/jcmm.15803 PMC7687014

[105] V. Lamontain , T. Schmid , D. Weber‐Steffens , et al., “Stimulation of TNF Receptor Type 2 Expands Regulatory T Cells and Ameliorates Established Collagen‐induced Arthritis in Mice,” Cellular & Molecular Immunology 16, no. 1 (2019): 65–74. 29375132 10.1038/cmi.2017.138 PMC6318277

[106] K. F. Baker , D. McDonald , G. Hulme , et al., “Single‐cell Insights Into Immune Dysregulation in Rheumatoid Arthritis Flare versus Drug‐free Remission,” Nature Communications 15, no. 1 (2024): 1063. 10.1038/s41467-024-45213-2 PMC10844292 38316770

[107] S. Yan , K. Kotschenreuther , S. Deng , and D. M. Kofler , “Regulatory T Cells in Rheumatoid Arthritis: Functions, Development, Regulation, and Therapeutic Potential,” Cellular and Molecular Life Sciences 79, no. 10 (2022): 533. 36173485 10.1007/s00018-022-04563-0 PMC9522664

[108] H. Sun , W. Gao , W. Pan , et al., “Tim3(+) Foxp3 (+) Treg Cells Are Potent Inhibitors of Effector T Cells and Are Suppressed in Rheumatoid Arthritis,” Inflammation 40, no. 4 (2017): 1342–1350. 28478516 10.1007/s10753-017-0577-6

[109] T. S. Sumida , N. T. Cheru , and D. A. Hafler , “The Regulation and Differentiation of Regulatory T Cells and Their Dysfunction in Autoimmune Diseases,” Nature Reviews Immunology 24, no. 7 (2024): 503–517. 10.1038/s41577-024-00994-x PMC11216899 38374298

[110] K. Hattori , S. Tanaka , D. Hashiba , et al., “Synovial Regulatory T Cells Expressing ST2 Deteriorate Joint Inflammation Through the Suppression of Immunoregulatory Eosinophils,” Journal of Autoimmunity 149 (2024): 103333. 39509740 10.1016/j.jaut.2024.103333

[111] M. E. Mickael , I. Bienkowska , and M. Sacharczuk , “An Update on the Evolutionary History of Bregs,” Genes (Basel) 13, no. 5 (2022): 890. 35627275 10.3390/genes13050890 PMC9141580

[112] D. Catalan , M. A. Mansilla , A. Ferrier , et al., “Immunosuppressive Mechanisms of Regulatory B Cells,” Frontiers in Immunology 12 (2021): 611795. 33995344 10.3389/fimmu.2021.611795 PMC8118522

[113] E. C. Rosser and C. Mauri , “Regulatory B Cells: Origin, Phenotype, and Function,” Immunity 42, no. 4 (2015): 607–612. 25902480 10.1016/j.immuni.2015.04.005

[114] Z. Banko , J. Pozsgay , T. Gati , B. Rojkovich , I. Ujfalussy , and G. Sarmay , “Regulatory B Cells in Rheumatoid Arthritis: Alterations in Patients Receiving Anti‐TNF Therapy,” Clinical Immunology 184 (2017): 63–69. 28506920 10.1016/j.clim.2017.05.012

[115] Z. Banko , J. Pozsgay , D. Szili , et al., “Induction and Differentiation of IL‐10‐Producing Regulatory B Cells From Healthy Blood Donors and Rheumatoid Arthritis Patients,” Journal of Immunology 198, no. 4 (2017): 1512–1520. 10.4049/jimmunol.1600218 28087671

[116] D. Cui , L. Zhang , J. Chen , et al., “Changes in Regulatory B Cells and Their Relationship With Rheumatoid Arthritis Disease Activity,” Clinical and Experimental Medicine 15, no. 3 (2015): 285–292. 25245953 10.1007/s10238-014-0310-9

[117] L. Xu , X. Liu , H. Liu , et al., “Impairment of Granzyme B‐Producing Regulatory B Cells Correlates With Exacerbated Rheumatoid Arthritis,” Frontiers in Immunology 8 (2017): 768. 28713386 10.3389/fimmu.2017.00768 PMC5491972

[118] E. R. Zacca , L. I. Onofrio , C. D. V. Acosta , et al., “PD‐L1(+) Regulatory B Cells Are Significantly Decreased in Rheumatoid Arthritis Patients and Increase after Successful Treatment,” Frontiers in Immunology 9 (2018): 2241. 30327652 10.3389/fimmu.2018.02241 PMC6174216

[119] M. G. Garimella , S. Kour , V. Piprode , et al., “Adipose‐Derived Mesenchymal Stem Cells Prevent Systemic Bone Loss in Collagen‐Induced Arthritis,” Journal of Immunology 195, no. 11 (2015): 5136–5148. 10.4049/jimmunol.1500332 PMC4654226 26538398

[120] Q. Zhao and L. K. Jung , “Frequency of CD19(+)CD24(hi)CD38(hi) Regulatory B Cells Is Decreased in Peripheral Blood and Synovial Fluid of Patients With Juvenile Idiopathic Arthritis: A Preliminary Study,” Pediatric Rheumatology Online Journal 16, no. 1 (2018): 44. 29973221 10.1186/s12969-018-0262-9 PMC6033228

[121] P. Fortea‐Gordo , A. Villalba , L. Nuno , et al., “Circulating CD19+CD24hiCD38hi Regulatory B Cells as Biomarkers of Response to Methotrexate in Early Rheumatoid Arthritis,” Rheumatology 59, no. 10 (2020): 3081–3091. 32417912 10.1093/rheumatology/keaa186

[122] J. Kim , H. J. Lee , I. S. Yoo , S. W. Kang , and J. H. Lee , “Regulatory B Cells Are Inversely Associated With Disease Activity in Rheumatoid Arthritis,” Yonsei Medical Journal 55, no. 5 (2014): 1354–1358. 25048496 10.3349/ymj.2014.55.5.1354 PMC4108823

[123] L. Luo , Q. Liu , S. Peng , et al., “The Number of Regulatory B Cells Is Increased in Mice With Collagen‐induced Arthritis,” Open Life Sciences 14 (2019): 12–18. 33817132 10.1515/biol-2019-0002 PMC7874759

[124] M. K. Park , Y. O. Jung , S. Y. Lee , et al., “Amelioration of Autoimmune Arthritis by Adoptive Transfer of Foxp3‐expressing Regulatory B Cells Is Associated With the Treg/Th17 Cell Balance,” Journal of Translational Medicine 14, no. 1 (2016): 191. 27350539 10.1186/s12967-016-0940-7 PMC4924280

[125] D. Mauro , R. Thomas , G. Guggino , R. Lories , M. A. Brown , and F. Ciccia , “Ankylosing Spondylitis: An Autoimmune or Autoinflammatory Disease?,” Nature Reviews Rheumatology 17, no. 7 (2021): 387–404. 34113018 10.1038/s41584-021-00625-y

[126] W. Zhu , X. He , K. Cheng , et al., “Ankylosing Spondylitis: Etiology, Pathogenesis, and Treatments,” Bone Research 7 (2019): 22. 31666997 10.1038/s41413-019-0057-8 PMC6804882

[127] Y. Xiong , M. Cai , Y. Xu , et al., “Joint Together: The Etiology and Pathogenesis of Ankylosing Spondylitis,” Frontiers in Immunology 13 (2022): 996103. 36325352 10.3389/fimmu.2022.996103 PMC9619093

[128] M. P. Crawford , S. Sinha , P. S. Renavikar , N. Borcherding , and N. J. Karandikar , “CD4 T Cell‐intrinsic Role for the T Helper 17 Signature Cytokine IL‐17: Effector Resistance to Immune Suppression,” Proceedings of the National Academy of Sciences of the United States of America 117, no. 32 (2020): 19408–19414. 32719138 10.1073/pnas.2005010117 PMC7430972

[129] I. Kryczek , E. Zhao , Y. Liu , et al., “Human TH17 Cells Are Long‐lived Effector Memory Cells,” Science Translational Medicine 3, no. 104 (2011): 104ra0. 10.1126/scitranslmed.3002949 PMC3345568 21998407

[130] F. M. Milanez , C. G. Saad , V. T. Viana , et al., “IL‐23/Th17 Axis Is Not Influenced by TNF‐blocking Agents in Ankylosing Spondylitis Patients,” Arthritis Research & Therapy 18 (2016): 52. 26912133 10.1186/s13075-016-0949-6 PMC4765065

[131] M. Faham , V. Carlton , M. Moorhead , et al., “Discovery of T Cell Receptor Beta Motifs Specific to HLA‐B27‐Positive Ankylosing Spondylitis by Deep Repertoire Sequence Analysis,” Arthritis & Rheumatology 69, no. 4 (2017): 774–784. 28002888 10.1002/art.40028

[132] A. L. Hanson , H. J. Nel , L. Bradbury , J. Phipps , R. Thomas , and K. A. Le Cao , “Altered Repertoire Diversity and Disease‐Associated Clonal Expansions Revealed by T Cell Receptor Immunosequencing in Ankylosing Spondylitis Patients,” Arthritis & Rheumatology 72, no. 8 (2020): 1289–1302. 32162785 10.1002/art.41252

[133] M. Zheng , X. Zhang , Y. Zhou , et al., “TCR Repertoire and CDR3 Motif Analyses Depict the Role of Alphabeta T Cells in Ankylosing Spondylitis,” EBioMedicine 47 (2019): 414–426. 31477563 10.1016/j.ebiom.2019.07.032 PMC6796593

[134] E. May , M. L. Dorris , N. Satumtira , et al., “CD8 alpha Beta T Cells Are Not Essential to the Pathogenesis of Arthritis or Colitis in HLA‐B27 Transgenic Rats,” Journal of Immunology 170, no. 2 (2003): 1099–1105. 10.4049/jimmunol.170.2.1099 12517979

[135] J. D. Taurog , M. L. Dorris , N. Satumtira , et al., “Spondylarthritis in HLA‐B27/human beta2‐microglobulin‐transgenic Rats Is Not Prevented by Lack of CD8,” Arthritis and Rheumatism 60, no. 7 (2009): 1977–1984. 19565478 10.1002/art.24599

[136] O. V. Britanova , K. R. Lupyr , D. B. Staroverov , et al., “Targeted Depletion of TRBV9(+) T Cells as Immunotherapy in a Patient With Ankylosing Spondylitis,” Nature Medicine 29, no. 11 (2023): 2731–2736. 10.1038/s41591-023-02613-z PMC10667094 37872223

[137] H. Appel , R. Maier , P. Wu , et al., “Analysis of IL‐17(+) Cells in Facet Joints of Patients With Spondyloarthritis Suggests That the Innate Immune Pathway Might be of Greater Relevance Than the Th17‐mediated Adaptive Immune Response,” Arthritis Research & Therapy 13, no. 3 (2011): R95. 21689402 10.1186/ar3370 PMC3218910

[138] N. Tamassia , F. Arruda‐Silva , F. Calzetti , et al., “A Reappraisal on the Potential Ability of Human Neutrophils to Express and Produce IL‐17 Family Members in Vitro: Failure to Reproducibly Detect It,” Frontiers in Immunology 9 (2018): 795. 29719541 10.3389/fimmu.2018.00795 PMC5913333

[139] H. L. Rosenzweig , E. E. Vance , K. Asare‐Konadu , et al., “Card9/neutrophil Signalling Axis Promotes IL‐17A‐mediated Ankylosing Spondylitis,” Annals of the Rheumatic Diseases 83, no. 2 (2024): 214–222. 37813481 10.1136/ard-2022-223146 PMC10850635

[140] C. Bridgewood , A. Watad , T. Russell , et al., “Identification of Myeloid Cells in the human Enthesis as the Main Source of Local IL‐23 Production,” Annals of the Rheumatic Diseases 78, no. 7 (2019): 929–933. 31018959 10.1136/annrheumdis-2018-214944 PMC6585277

[141] F. Ciccia , G. Guggino , M. Zeng , et al., “Proinflammatory CX3CR1+CD59+Tumor Necrosis Factor‐Like Molecule 1A+Interleukin‐23+ Monocytes Are Expanded in Patients with Ankylosing Spondylitis and Modulate Innate Lymphoid Cell 3 Immune Functions,” Arthritis & Rheumatology 70, no. 12 (2018): 2003–2013. 29869839 10.1002/art.40582

[142] V. Ranganathan , F. Ciccia , F. Zeng , et al., “Macrophage Migration Inhibitory Factor Induces Inflammation and Predicts Spinal Progression in Ankylosing Spondylitis,” Arthritis & Rheumatology 69, no. 9 (2017): 1796–1806. 28597514 10.1002/art.40175

[143] M. Bollow , T. Fischer , H. Reisshauer , et al., “Quantitative Analyses of Sacroiliac Biopsies in Spondyloarthropathies: T Cells and Macrophages Predominate in Early and Active Sacroiliitis‐ cellularity Correlates With the Degree of Enhancement Detected by Magnetic Resonance Imaging,” Annals of the Rheumatic Diseases 59, no. 2 (2000): 135–140. 10666170 10.1136/ard.59.2.135 PMC1753076

[144] D. McGonagle , H. Marzo‐Ortega , P. O'Connor , W. Gibbon , P. Hawkey , and K. Henshaw , “Histological Assessment of the Early Enthesitis Lesion in Spondyloarthropathy,” Annals of the Rheumatic Diseases 61, no. 6 (2002): 534–537. 12006328 10.1136/ard.61.6.534 PMC1754106

[145] F. Tavasolian , S. Lively , C. Pastrello , et al., “Proteomic and Genomic Profiling of Plasma Exosomes From Patients With Ankylosing Spondylitis,” Annals of the Rheumatic Diseases 82, no. 11 (2023): 1429–1443. 37532285 10.1136/ard-2022-223791

[146] D. Liu , B. Liu , C. Lin , and J. Gu , “Imbalance of Peripheral Lymphocyte Subsets in Patients with Ankylosing Spondylitis: A Meta‐Analysis,” Frontiers in Immunology 12 (2021): 696973. 34295337 10.3389/fimmu.2021.696973 PMC8291033

[147] H. T. Liao and C. Y. Tsai , “Cytokines and Regulatory T Cells in Ankylosing Spondylitis,” Bone & Joint Research 12, no. 2 (2023): 133–137. 37051816 10.1302/2046-3758.122.BJR-2022-0195.R1 PMC10003037

[148] D. Simone , F. Penkava , A. Ridley , S. Sansom , M. H. Al‐Mossawi , and P. Bowness , “Single Cell Analysis of Spondyloarthritis Regulatory T Cells Identifies Distinct Synovial Gene Expression Patterns and Clonal Fates,” Communications Biology 4, no. 1 (2021): 1395. 34907325 10.1038/s42003-021-02931-3 PMC8671562

[149] L. M. Araujo , I. Fert , Q. Jouhault , et al., “Increased Production of Interleukin‐17 Over Interleukin‐10 by Treg Cells Implicates Inducible Costimulator Molecule in Experimental Spondyloarthritis,” Arthritis & Rheumatology 66, no. 9 (2014): 2412–2422. 24909668 10.1002/art.38737

[150] Y. F. Liu , K. H. Zhuang , B. Chen , et al., “Expansion and Activation of Monocytic‐myeloid‐derived Suppressor Cell via STAT3/Arginase‐I Signaling in Patients With Ankylosing Spondylitis,” Arthritis Research & Therapy 20, no. 1 (2018): 168. 30075733 10.1186/s13075-018-1654-4 PMC6091075

[151] C. T. Ritchlin , R. A. Colbert , and D. D. Gladman , “Psoriatic Arthritis,” New England Journal of Medicine 376, no. 10 (2017): 957–970. 28273019 10.1056/NEJMra1505557

[152] D. J. Veale and U. Fearon , “The Pathogenesis of Psoriatic Arthritis,” Lancet 391, no. 10136 (2018): 2273–2284. 29893226 10.1016/S0140-6736(18)30830-4

[153] O. FitzGerald , A. Ogdie , V. Chandran , et al., “Psoriatic Arthritis,” Nature Reviews Disease Primers 7, no. 1 (2021): 59. 10.1038/s41572-021-00293-y 34385474

[154] M. Kamata and Y. Tada , “Dendritic Cells and Macrophages in the Pathogenesis of Psoriasis,” Frontiers in Immunology 13 (2022): 941071. 35837394 10.3389/fimmu.2022.941071 PMC9274091

[155] A. Deodhar , P. S. Helliwell , W. H. Boehncke , et al., “Guselkumab in Patients With Active Psoriatic Arthritis Who Were Biologic‐naive or Had Previously Received TNFalpha Inhibitor Treatment (DISCOVER‐1): A Double‐blind, Randomised, Placebo‐controlled Phase 3 Trial,” Lancet 395, no. 10230 (2020): 1115–1125. 32178765 10.1016/S0140-6736(20)30265-8

[156] I. B. McInnes , A. Kavanaugh , A. B. Gottlieb , et al., “Efficacy and Safety of ustekinumab in Patients With Active Psoriatic Arthritis: 1 Year Results of the Phase 3, Multicentre, Double‐blind, Placebo‐controlled PSUMMIT 1 Trial,” Lancet 382, no. 9894 (2013): 780–789. 23769296 10.1016/S0140-6736(13)60594-2

[157] P. J. Mease , P. Rahman , A. B. Gottlieb , et al., “Guselkumab in Biologic‐naive Patients With Active Psoriatic Arthritis (DISCOVER‐2): A Double‐blind, Randomised, Placebo‐controlled Phase 3 Trial,” Lancet 395, no. 10230 (2020): 1126–1136. 32178766 10.1016/S0140-6736(20)30263-4

[158] C. Ritchlin , P. Rahman , A. Kavanaugh , et al., “Efficacy and Safety of the Anti‐IL‐12/23 p40 Monoclonal Antibody, ustekinumab, in Patients With Active Psoriatic Arthritis Despite Conventional Non‐biological and Biological Anti‐tumour Necrosis Factor Therapy: 6‐month and 1‐year Results of the Phase 3, Multicentre, Double‐blind, Placebo‐controlled, Randomised PSUMMIT 2 Trial,” Annals of the Rheumatic Diseases 73, no. 6 (2014): 990–999. 24482301 10.1136/annrheumdis-2013-204655 PMC4033144

[159] H. Benham , P. Norris , J. Goodall , et al., “Th17 and Th22 Cells in Psoriatic Arthritis and Psoriasis,” Arthritis Research & Therapy 15, no. 5 (2013): R136. 24286492 10.1186/ar4317 PMC3978433

[160] S. P. Raychaudhuri , S. K. Raychaudhuri , and M. C. Genovese , “IL‐17 Receptor and Its Functional Significance in Psoriatic Arthritis,” Molecular and Cellular Biochemistry 359, no. 1‐2 (2012): 419–429. 21894442 10.1007/s11010-011-1036-6

[161] C. T. Ritchlin and J. G. Krueger , “New Therapies for Psoriasis and Psoriatic Arthritis,” Current Opinion in Rheumatology 28, no. 3 (2016): 204–210. 27022911 10.1097/BOR.0000000000000274 PMC5812682

[162] F. Ciccia , G. Guggino , A. Ferrante , et al., “Interleukin‐9 Overexpression and Th9 Polarization Characterize the Inflamed Gut, the Synovial Tissue, and the Peripheral Blood of Patients with Psoriatic Arthritis,” Arthritis & Rheumatology 68, no. 8 (2016): 1922–1931. 26895441 10.1002/art.39649

[163] W. Tillett , J. F. Merola , D. Thaci , et al., “Disease Characteristics and the Burden of Joint and Skin Involvement amongst People with Psoriatic Arthritis: A Population Survey,” Rheumatology and Therapy 7, no. 3 (2020): 617–637. 32700230 10.1007/s40744-020-00221-8 PMC7410983

[164] P. E. Saw and E. Song , “The 'inflammazone' in Chronic Inflammatory Diseases: Psoriasis and Sarcoidosis,” Trends in Immunology 46, no. 2 (2025): 121–137. 39875239 10.1016/j.it.2025.01.002

[165] Q. Y. Su , S. X. Zhang , L. Yang , et al., “Peripheral T(reg) Levels and Transforming Growth Factor‐beta (TGFbeta) in Patients With Psoriatic Arthritis: A Systematic Review Meta‐Analysis,” Advances in Therapy 40, no. 1 (2023): 102–116. 36287319 10.1007/s12325-022-02337-5

[166] A. Szentpetery , E. Heffernan , M. Gogarty , et al., “Abatacept Reduces Synovial Regulatory T‐cell Expression in Patients With Psoriatic Arthritis,” Arthritis Research & Therapy 19, no. 1 (2017): 158. 28679449 10.1186/s13075-017-1364-3 PMC5498994

[167] T. McTaggart , J. X. Lim , K. J. Smith , et al., “Deep Phenotyping of T Regulatory Cells in Psoriatic Arthritis Highlights Targetable Mechanisms of Disease,” Journal of Biological Chemistry 301, no. 1 (2025): 108059. 39662827 10.1016/j.jbc.2024.108059 PMC11750473

[168] D. X. Nguyen , H. M. Baldwin , A. N. Ezeonyeji , M. R. Butt , and M. R. Ehrenstein , “Regulatory T Cells Enhance Th17 Migration in Psoriatic Arthritis Which Is Reversed by Anti‐TNF,” Iscience 24, no. 9 (2021): 102973. 34471865 10.1016/j.isci.2021.102973 PMC8387926

[169] T. Fukasawa , T. Yamashita , A. Enomoto , et al., “The Optimal Use of tildrakizumab in the Elderly via Improvement of Treg Function and Its Preventive Effect of Psoriatic Arthritis,” Frontiers in Immunology 14 (2023): 1286251. 37928519 10.3389/fimmu.2023.1286251 PMC10620742

[170] Y. Liu , W. Jarjour , N. Olsen , and S. G. Zheng , “Traitor or Warrior‐Treg Cells Sneaking Into the Lesions of Psoriatic Arthritis,” Clinical Immunology 215 (2020): 108425. 32305454 10.1016/j.clim.2020.108425

[171] M. E. DeWane , R. Waldman , and J. Lu , “Dermatomyositis: Clinical Features and Pathogenesis,” Journal of the American Academy of Dermatology 82, no. 2 (2020): 267–281. 31279808 10.1016/j.jaad.2019.06.1309

[172] M. C. Dalakas , “Inflammatory Muscle Diseases,” New England Journal of Medicine 372, no. 18 (2015): 1734–1747. 25923553 10.1056/NEJMra1402225

[173] R. Waldman , M. E. DeWane , and J. Lu , “Dermatomyositis: Diagnosis and Treatment,” Journal of the American Academy of Dermatology 82, no. 2 (2020): 283–296. 31279813 10.1016/j.jaad.2019.05.105

[174] Y. Ma , J. Lai , Q. Wan , Z. Chen , L. Sun , and Q. Zhang , “Identification of Common Mechanisms and Biomarkers for Dermatomyositis and Atherosclerosis Based on Bioinformatics Analysis,” Skin Research and Technology: Official Journal of International Society for Bioengineering and the Skin (Isbs) [And] International Society for Digital Imaging of Skin (Isdis) [And] International Society for Skin Imaging (Issi) 30, no. 6 (2024): e13808. 10.1111/srt.13808 PMC11187814 38899746

[175] Y. Zhang , L. Shan , D. Li , et al., “Identification of Key Biomarkers Associated With Immune Cells Infiltration for Myocardial Injury in Dermatomyositis by Integrated Bioinformatics Analysis,” Arthritis Research & Therapy 25, no. 1 (2023): 69. 37118825 10.1186/s13075-023-03052-4 PMC10142164

[176] X. Lu , Q. Peng , and G. Wang , “Biomarkers of Disease Activity in Dermatomyositis,” Current Opinion in Rheumatology 34, no. 6 (2022): 289–294. 36082751 10.1097/BOR.0000000000000905

[177] X. Xu , T. Qiu , K. Sun , et al., “Integrated Analysis of Dermatomyositis Reveals Heterogeneous Immune Infiltration and Interstitial Lung Disease‐associated Endotype,” Arthritis Research & Therapy 27, no. 1 (2025): 26. 39923079 10.1186/s13075-025-03494-y PMC11806601

[178] T. Jiang , Y. Huang , H. Liu , et al., “Reduced miR‐146a Promotes REG3A Expression and Macrophage Migration in Polymyositis and Dermatomyositis,” Frontiers in Immunology 11 (2020): 37. 32153557 10.3389/fimmu.2020.00037 PMC7047152

[179] A. Kokuzawa , J. Nakamura , Y. Kamata , and K. Sato , “Potential Role of Type I Interferon/IP‐10 Axis in the Pathogenesis of Anti‐MDA5 Antibody‐positive Dermatomyositis,” Clinical and Experimental Rheumatology 41, no. 2 (2023): 275–284. 36622131 10.55563/clinexprheumatol/em67zx

[180] M. Kogami , Y. Abe , T. Ando , A. Makiyama , K. Yamaji , and N. Tamura , “Changes in Anti‐MDA5 Antibody Titres and Serum Cytokine Levels Before and After Diagnosis of Anti‐MDA5 Antibody‐positive Dermatomyositis,” Rheumatology 62, no. 7 (2023): 2525–2533. 36326436 10.1093/rheumatology/keac627

[181] J. Shi , X. Pei , J. Peng , et al., “Monocyte‐macrophage Dynamics as Key in Disparate Lung and Peripheral Immune Responses in Severe Anti‐melanoma Differentiation‐associated Gene 5‐positive Dermatomyositis‐related Interstitial Lung Disease,” Clinical and Translational Medicine 15, no. 2 (2025): e70226. 39902678 10.1002/ctm2.70226 PMC11791760

[182] W. Li , C. Deng , H. Yang , et al., “Expansion of Circulating Peripheral TIGIT+CD226+ CD4 T Cells With Enhanced Effector Functions in Dermatomyositis,” Arthritis Research & Therapy 23, no. 1 (2021): 15. 33413573 10.1186/s13075-020-02397-4 PMC7791775

[183] X. Hou , C. Yang , M. Lin , et al., “Altered Peripheral Helper T Cells in Peripheral Blood and Muscle Tissue of the Patients With Dermatomyositis,” Clinical and Experimental Medicine 21, no. 4 (2021): 655–661. 33900488 10.1007/s10238-021-00713-z

[184] N. Okiyama , Y. Ichimura , M. Shobo , et al., “Immune Response to Dermatomyositis‐specific Autoantigen, Transcriptional Intermediary Factor 1gamma Can Result in Experimental Myositis,” Annals of the Rheumatic Diseases 80, no. 9 (2021): 1201–1208. 33811031 10.1136/annrheumdis-2020-218661

[185] X. Chen , D. Lian , and H. Zeng , “Single‐cell Profiling of Peripheral Blood and Muscle Cells Reveals Inflammatory Features of Juvenile Dermatomyositis,” Frontiers in Cell and Developmental Biology 11 (2023): 1166017. 37152289 10.3389/fcell.2023.1166017 PMC10157079

[186] J. S. Gofshteyn , L. Mansfield , J. Spitznagle , et al., “Association of Juvenile Dermatomyositis Disease Activity with the Expansion of Blood Memory B and T Cell Subsets Lacking Follicular Markers,” Arthritis & Rheumatology 75, no. 7 (2023): 1246–1261. 36648920 10.1002/art.42446

[187] X. Lu , Q. Peng , and G. Wang , “Anti‐MDA5 Antibody‐positive Dermatomyositis: Pathogenesis and Clinical Progress,” Nature Reviews Rheumatology 20, no. 1 (2024): 48–62. 38057474 10.1038/s41584-023-01054-9

[188] Y. Guo , H. Liu , B. Chen , et al., “Dysregulated CD38 Expression on T Cells Was Associated With Rapidly Progressive Interstitial Lung Disease in Anti‐melanoma Differentiation‐associated Gene 5 Positive Dermatomyositis,” Frontiers in Immunology 15 (2024): 1455944. 39588376 10.3389/fimmu.2024.1455944 PMC11586385

[189] Y. Ye , Z. Chen , S. Jiang , et al., “Single‐cell Profiling Reveals Distinct Adaptive Immune Hallmarks in MDA5+ Dermatomyositis With Therapeutic Implications,” Nature Communications 13, no. 1 (2022): 6458. 10.1038/s41467-022-34145-4 PMC9617246 36309526

[190] Y. Zuo , L. Ye , F. Chen , et al., “Different Multivariable Risk Factors for Rapid Progressive Interstitial Lung Disease in Anti‐MDA5 Positive Dermatomyositis and Anti‐Synthetase Syndrome,” Frontiers in Immunology 13 (2022): 845988. 35320936 10.3389/fimmu.2022.845988 PMC8936070

[191] M. Ogawa‐Momohara , T. Vazquez , F. Chin , M. Sharma , J. Dan , and G. Sprow , “Multiplexed Mass Cytometry of Cutaneous Lupus Erythematosus and Dermatomyositis Skin: An in‐depth, B‐Cell‐Directed Immunoprofile,” Journal of Investigative Dermatology 145, no. 1 (2025): 190–193. e2. 39098475 10.1016/j.jid.2024.07.007 PMC13157705

[192] J. He , Z. Liu , Y. Cao , et al., “Single‐cell Landscape of Peripheral Immune Response in Patients With Anti‐melanoma Differentiation‐associated Gene 5 Dermatomyositis,” Rheumatology 63, no. 8 (2024): 2284–2294. 37941459 10.1093/rheumatology/kead597

[193] O. Krystufkova , H. Hulejova , H. F. Mann , et al., “Serum Levels of B‐cell Activating Factor of the TNF family (BAFF) Correlate With Anti‐Jo‐1 Autoantibodies Levels and Disease Activity in Patients With Anti‐Jo‐1positive Polymyositis and Dermatomyositis,” Arthritis Research & Therapy 20, no. 1 (2018): 158. 30053824 10.1186/s13075-018-1650-8 PMC6062864

[194] Y. Shi , H. You , C. Liu , et al., “Elevated Serum B‐cell Activator Factor Levels Predict Rapid Progressive Interstitial Lung Disease in Anti‐melanoma Differentiation Associated Protein 5 Antibody Positive Dermatomyositis,” Orphanet Journal of Rare Diseases 19, no. 1 (2024): 170. 38637830 10.1186/s13023-024-03153-6 PMC11027411

[195] C. Costin , A. Khojah , E. Ochfeld , et al., “B Cell Lymphocytosis in Juvenile Dermatomyositis,” Diagnostics (Basel) 13, no. 16 (2023): 2626. 37627885 10.3390/diagnostics13162626 PMC10453137

[196] J. Radke , R. Koll , C. Preusse , et al., “Architectural B‐cell Organization in Skeletal Muscle Identifies Subtypes of Dermatomyositis,” Neurology Neuroimmunology & Neuroinflammation 5, no. 3 (2018): e451. 10.1212/NXI.0000000000000451 PMC5840889 29520367

[197] J. Neely , G. Hartoularos , D. Bunis , et al., “Multi‐Modal Single‐Cell Sequencing Identifies Cellular Immunophenotypes Associated with Juvenile Dermatomyositis Disease Activity,” Frontiers in Immunology 13 (2022): 902232. 35799782 10.3389/fimmu.2022.902232 PMC9254730

[198] G. Rabadam , C. Wibrand , E. Flynn , et al., “Coordinated Immune Dysregulation in Juvenile Dermatomyositis Revealed by Single‐cell Genomics,” JCI Insight 9, no. 12 (2024): e176963. 38743491 10.1172/jci.insight.176963 PMC11383589

[199] C. J. M. Piper , M. G. L. Wilkinson , C. T. Deakin , et al., “CD19(+)CD24(hi)CD38(hi) B Cells Are Expanded in Juvenile Dermatomyositis and Exhibit a Pro‐Inflammatory Phenotype after Activation through Toll‐Like Receptor 7 and Interferon‐alpha,” Frontiers in Immunology 9 (2018): 1372. 29988398 10.3389/fimmu.2018.01372 PMC6024011

[200] A. Bukhari , A. Khojah , W. Marin , et al., “Increased Otoferlin Expression in B Cells Is Associated With Muscle Weakness in Untreated Juvenile Dermatomyositis: A Pilot Study,” International Journal of Molecular Sciences 24, no. 13 (2023): 10553. 37445728 10.3390/ijms241310553 PMC10341737

[201] F. Coutant , R. Bachet , J. J. Pin , M. Alonzo , and P. Miossec , “Monoclonal Antibodies From B Cells of Patients With Anti‐MDA5 Antibody‐positive Dermatomyositis Directly Stimulate Interferon Gamma Production,” Journal of Autoimmunity 130 (2022): 102831. 35436746 10.1016/j.jaut.2022.102831

[202] R. Nicolai , P. Merli , P. Moran Alvarez , C. Bracaglia , F. Del Bufalo , and E. Marasco , “Autologous CD19‐Targeting CAR T Cells in a Patient with Refractory Juvenile Dermatomyositis,” Arthritis & Rheumatology 76, no. 10 (2024): 1560–1565. 38924652 10.1002/art.42933

[203] A. Paris‐Munoz , R. M. Alcobendas‐Rueda , C. Verdu‐Sanchez , et al., “CD19 CAR‐T Cell Therapy in a Pediatric Patient With MDA5(+) Dermatomyositis and Rapidly Progressive Interstitial Lung Disease,” Med 6, no. 8 (2025): 100676. 40306284 10.1016/j.medj.2025.100676

[204] Y. Liu , Y. Li , T. Shen , et al., “Belimumab Ameliorates Symptoms and Disease Activity in Patients With Dermatomyositis and Juvenile Dermatomyositis Refractory to Standard Therapy: A Retrospective Observational Study,” Journal of the American Academy of Dermatology 91, no. 3 (2024): 524–527. 38697217 10.1016/j.jaad.2024.04.060

[205] M. T. Holzer , J. F. Nies , T. Oqueka , T. B. Huber , I. Kotter , and M. Krusche , “Successful Rescue Therapy with Daratumumab in Rapidly Progressive Interstitial Lung Disease Caused by MDA5‐Positive Dermatomyositis,” Chest 163, no. 1 (2023): e1–e5. 36628678 10.1016/j.chest.2022.08.2209

[206] E. Antiga , C. C. Kretz , R. Klembt , et al., “Characterization of Regulatory T Cells in Patients With Dermatomyositis,” Journal of Autoimmunity 35, no. 4 (2010): 342–350. 20843660 10.1016/j.jaut.2010.07.006

[207] M. Feng , H. Guo , C. Zhang , et al., “Absolute Reduction of Regulatory T Cells and Regulatory Effect of Short‐term and Low‐dose IL‐2 in Polymyositis or Dermatomyositis,” International Immunopharmacology 77 (2019): 105912. 31669890 10.1016/j.intimp.2019.105912

[208] S. X. Zhang , J. Wang , H. H. Sun , et al., “Circulating Regulatory T Cells Were Absolutely Decreased in Dermatomyositis/Polymyositis Patients and Restored by Low‐dose IL‐2,” Annals of the Rheumatic Diseases 80, no. 8 (2021): e130. 31611221 10.1136/annrheumdis-2019-216246

[209] Y. Xie , T. Zhang , R. Su , et al., “Increased Serum Soluble Interleukin‐2 Receptor Levels in Dermatomyositis Are Associated With Th17/Treg Immune Imbalance,” Clinical and Experimental Medicine 23, no. 7 (2023): 3605–3617. 37528249 10.1007/s10238-023-01155-5

[210] Z. Zhang , J. Pang , Y. Li , Y. Zuo , X. Cui , and H. Xu , “Imbalance of Peripheral Blood Th17/Treg Increases Neutrophil‐to‐lymphocyte Ratio in Patients With Dermatomyositis,” American Journal of Translational Research 15, no. 10 (2023): 6106–6114. 37969179 PMC10641349

[211] X. Zheng , R. Su , F. Hu , et al., “Low‐dose IL‐2 Therapy Restores Imbalance Between Th17 and Regulatory T Cells in Patients With the Dermatomyositis Combined With EBV/CMV Viremia,” Autoimmunity Reviews 21, no. 11 (2022): 103186. 36084894 10.1016/j.autrev.2022.103186

[212] E. Sag , G. Kale , G. Haliloglu , et al., “Inflammatory Milieu of Muscle Biopsies in Juvenile Dermatomyositis,” Rheumatology International 41, no. 1 (2021): 77–85. 33106894 10.1007/s00296-020-04735-w

[213] Y. Vercoulen , F. Bellutti Enders , J. Meerding , M. Plantinga , E. F. Elst , and H. Varsani , “Increased Presence of FOXP3+ Regulatory T Cells in Inflamed Muscle of Patients With Active Juvenile Dermatomyositis Compared to Peripheral Blood,” PLoS ONE 9, no. 8 (2014): e105353. 25157414 10.1371/journal.pone.0105353 PMC4144849

[214] Y. Chen , H. Liu , Z. Luo , et al., “ASM Is a Therapeutic Target in Dermatomyositis by Regulating the Differentiation of Naive CD4 + T Cells Into Th17 and Treg Subsets,” Skeletal Muscle 14, no. 1 (2024): 16. 39026344 10.1186/s13395-024-00347-1 PMC11256435

[215] W. Yan , L. Wang , Z. Chen , et al., “Knockdown of lncRNA HAGLR Promotes Treg Cell Differentiation Through Increasing the RUNX3 Level in Dermatomyositis,” Journal of Molecular Histology 53, no. 2 (2022): 413–421. 35064420 10.1007/s10735-021-10051-9

[216] J. M. Pandya , I. Loell , M. S. Hossain , et al., “Effects of Conventional Immunosuppressive Treatment on CD244+ (CD28null) and FOXP3+ T Cells in the Inflamed Muscle of Patients With Polymyositis and Dermatomyositis,” Arthritis Research & Therapy 18 (2016): 80. 27039301 10.1186/s13075-016-0974-5 PMC4818535

[217] I. E. Lundberg , M. Fujimoto , J. Vencovsky , et al., “Idiopathic Inflammatory Myopathies,” Nature Reviews Disease Primers 7, no. 1 (2021): 86. 10.1038/s41572-021-00321-x 34857798

[218] V. Leclair , A. Notarnicola , J. Vencovsky , and I. E. Lundberg , “Polymyositis: Does It Really Exist as a Distinct Clinical Subset?,” Current Opinion in Rheumatology 33, no. 6 (2021): 537–543. 34494607 10.1097/BOR.0000000000000837

[219] Q. Jia , R. J. Hao , X. J. Lu , et al., “Identification of Hub Biomarkers and Immune Cell Infiltration Characteristics of Polymyositis by Bioinformatics Analysis,” Frontiers in Immunology 13 (2022): 1002500. 36225941 10.3389/fimmu.2022.1002500 PMC9548705

[220] M. Kamiya , F. Mizoguchi , A. Takamura , N. Kimura , K. Kawahata , and H. Kohsaka , “A New in Vitro Model of Polymyositis Reveals CD8+ T Cell Invasion Into Muscle Cells and Its Cytotoxic Role,” Rheumatology 59, no. 1 (2020): 224–232. 31257434 10.1093/rheumatology/kez248 PMC6927901

[221] M. Houtman , L. Ekholm , E. Hesselberg , et al., “T‐cell Transcriptomics From Peripheral Blood Highlights Differences Between Polymyositis and Dermatomyositis Patients,” Arthritis Research & Therapy 20, no. 1 (2018): 188. 30157932 10.1186/s13075-018-1688-7 PMC6116372

[222] Y. Shimojima , M. Matsuda , W. Ishii , D. Kishida , and Y. Sekijima , “T‐cell Receptor‐mediated Characteristic Signaling Pathway of Peripheral Blood T Cells in Dermatomyositis and Polymyositis,” Autoimmunity 50, no. 8 (2017): 481–490. 29172719 10.1080/08916934.2017.1405942

[223] Z. Zhu , C. Yang , J. Wang , Q. Feng , Q. Chen , and P. Yang , “Altered Chemokine Receptor Expression in the Peripheral Blood Lymphocytes in Polymyositis and Dermatomyositis,” Cytokine 99 (2017): 316–321. 28869080 10.1016/j.cyto.2017.08.018

[224] H. Hasegawa , K. Kawahata , F. Mizoguchi , N. Okiyama , N. Miyasaka , and H. Kohsaka , “Direct Suppression of Autoaggressive CD8+ T Cells With CD80/86 Blockade in CD8+ T Cell‐mediated Polymyositis Models of Mice,” Clinical and Experimental Rheumatology 35, no. 4 (2017): 593–597. 28134083

[225] J. M. Pandya , P. Venalis , L. Al‐Khalili , et al., “CD4+ and CD8+ CD28(null) T Cells Are Cytotoxic to Autologous Muscle Cells in Patients with Polymyositis,” Arthritis & Rheumatology 68, no. 8 (2016): 2016–2026. 26895511 10.1002/art.39650

[226] S. John , S. J. Antonia , T. A. Rose , et al., “Progressive Hypoventilation due to Mixed CD8(+) and CD4(+) Lymphocytic Polymyositis Following tremelimumab—durvalumab Treatment,” Journal for ImmunoTherapy of Cancer 5, no. 1 (2017): 54. 28716137 10.1186/s40425-017-0258-x PMC5514517

[227] T. Kimura , S. Fukushima , A. Miyashita , et al., “Myasthenic Crisis and Polymyositis Induced by One Dose of nivolumab,” Cancer Science 107, no. 7 (2016): 1055–1058. 27420474 10.1111/cas.12961 PMC4946722

[228] P. Xia , Y. Q. Shao , C. C. Yu , Y. Xie , and Z. J. Zhou , “NLRP3 inflammasome Up‐regulates Major Histocompatibility Complex Class I Expression and Promotes Inflammatory Infiltration in Polymyositis,” BMC Immunology [Electronic Resource] 23, no. 1 (2022): 39. 35965334 10.1186/s12865-022-00515-2 PMC9375941

[229] W. Yan , C. Chen , and H. Chen , “Estrogen Downregulates miR‐21 Expression and Induces Inflammatory Infiltration of Macrophages in Polymyositis: Role of CXCL10,” Molecular Neurobiology 54, no. 3 (2017): 1631–1641. 26873848 10.1007/s12035-016-9769-6

[230] W. Yan , W. Fan , C. Chen , Y. Wu , Z. Fan , and J. Chen , “IL‐15 Up‐regulates the MMP‐9 Expression Levels and Induces Inflammatory Infiltration of Macrophages in Polymyositis Through Regulating the NF‐kB Pathway,” Gene 591, no. 1 (2016): 137–147. 27374114 10.1016/j.gene.2016.06.055

[231] Y. Liu , Y. Gao , J. Yang , C. Shi , Y. Wang , and Y. Xu , “MicroRNA‐381 Reduces Inflammation and Infiltration of Macrophages in Polymyositis via Downregulating HMGB1,” International Journal of Oncology 53, no. 3 (2018): 1332–1342. 29956737 10.3892/ijo.2018.4463

[232] D. Wu , Y. Cui , Y. Cao , et al., “Clinical Implications and Mechanism of Complement C1q in Polymyositis,” Applied Biochemistry and Biotechnology 196, no. 6 (2024): 3088–3101. 37624510 10.1007/s12010-023-04692-7

[233] Y. Zhou , Y. Zhao , G. Yin , L. Kang , X. Zhu , and Q. Xie , “CD44 is Associated With Muscle Inflammation in Polymyositis and Skin Damage in Idiopathic Inflammatory Myopathy,” Clinical and Experimental Rheumatology 43, no. 2 (2025): 241–250. 39269020 10.55563/clinexprheumatol/hlk85n

[234] Y. Enomoto , Y. Suzuki , H. Hozumi , et al., “Clinical Significance of Soluble CD163 in Polymyositis‐related or Dermatomyositis‐related Interstitial Lung Disease,” Arthritis Research & Therapy 19, no. 1 (2017): 9. 28103926 10.1186/s13075-016-1214-8 PMC5248519

[235] Q. L. Peng , Y. L. Zhang , X. M. Shu , et al., “Elevated Serum Levels of Soluble CD163 in Polymyositis and Dermatomyositis: Associated With Macrophage Infiltration in Muscle Tissue,” Journal of Rheumatology 42, no. 6 (2015): 979–987. 25877505 10.3899/jrheum.141307

[236] X. Yang , H. Yao , Q. Zhao , et al., “UNC13D mutation in a Patient With Juvenile Polymyositis With Recurrent Macrophage Activation Syndrome,” Rheumatology 60, no. 11 (2021): e404–e406. 33930104 10.1093/rheumatology/keab391

[237] S. Koh , H. Koh , Y. Nakashima , et al., “Plasma Kinetics of Th1, Th2 and Th17 Cytokines in Polymyositis Related to Chronic Graft‐versus‐Host Disease,” Internal Medicine 55, no. 16 (2016): 2265–2270. 27523006 10.2169/internalmedicine.55.6206

[238] R. Anan , M. Akiyama , Y. Kaneko , et al., “Polymyositis With Elevated Serum IgG4 Levels and Abundant IgG4+ Plasma Cell Infiltration: A Case Report and Literature Review,” Medicine 96, no. 48 (2017): e8710. 29310344 10.1097/MD.0000000000008710 PMC5728745

[239] A. Tjarnlund , Q. Tang , C. Wick , et al., “Abatacept in the Treatment of Adult Dermatomyositis and Polymyositis: A Randomised, Phase IIb Treatment Delayed‐start Trial,” Annals of the Rheumatic Diseases 77, no. 1 (2018): 55–62. 28993346 10.1136/annrheumdis-2017-211751

[240] J. Zhao , X. J. Guo , and L. Shi , “Inflammatory Biomarkers in Polymyositis/Dermatomyositis Patients With Interstitial Lung Disease: A Retrospective Study,” Current Medical Research and Opinion 40, no. 1 (2024): 113–122. 37938089 10.1080/03007995.2023.2281501

[241] D. J. Hunter , L. March , and M. Chew , “Osteoarthritis in 2020 and Beyond: A Lancet Commission,” Lancet 396, no. 10264 (2020): 1711–1712. 33159851 10.1016/S0140-6736(20)32230-3

[242] F. Motta , E. Barone , A. Sica , and C. Selmi , “Inflammaging and Osteoarthritis,” Clinical Reviews in Allergy & Immunology 64, no. 2 (2023): 222–238. 35716253 10.1007/s12016-022-08941-1

[243] N. Amos , S. Lauder , A. Evans , M. Feldmann , and J. Bondeson , “Adenoviral Gene Transfer Into Osteoarthritis Synovial Cells Using the Endogenous Inhibitor IkappaBalpha Reveals That Most, but Not All, Inflammatory and Destructive Mediators Are NFkappaB Dependent,” Rheumatology 45, no. 10 (2006): 1201–1209. 16571608 10.1093/rheumatology/kel078

[244] W. C. Chang , M. T. Chu , C. Y. Hsu , et al., “Rhein, an Anthraquinone Drug, Suppresses the NLRP3 Inflammasome and Macrophage Activation in Urate Crystal‐Induced Gouty Inflammation,” American Journal of Chinese Medicine 47, no. 1 (2019): 135–151. 30612459 10.1142/S0192415X19500071

[245] J. Sokolove and C. M. Lepus , “Role of Inflammation in the Pathogenesis of Osteoarthritis: Latest Findings and Interpretations,” Therapeutic Advances in Musculoskeletal Disease 5, no. 2 (2013): 77–94. 23641259 10.1177/1759720X12467868 PMC3638313

[246] H. Zhang , D. Cai , and X. Bai , “Macrophages Regulate the Progression of Osteoarthritis,” Osteoarthritis and Cartilage 28, no. 5 (2020): 555–561. 31982565 10.1016/j.joca.2020.01.007

[247] S. Samavedi , P. Diaz‐Rodriguez , J. D. Erndt‐Marino , and M. S. Hahn , “A Three‐Dimensional Chondrocyte‐Macrophage Coculture System to Probe Inflammation in Experimental Osteoarthritis,” Tissue Engineering Part A 23, no. 3‐4 (2017): 101–114. 27736317 10.1089/ten.tea.2016.0007 PMC5312455

[248] E. N. Blaney Davidson , P. M. van der Kraan , and W. B. van den Berg , “TGF‐beta and Osteoarthritis,” Osteoarthritis and Cartilage 15, no. 6 (2007): 597–604. 17391995 10.1016/j.joca.2007.02.005

[249] H. Zhang , C. Lin , C. Zeng , et al., “Synovial Macrophage M1 Polarisation Exacerbates Experimental Osteoarthritis Partially Through R‐spondin‐2,” Annals of the Rheumatic Diseases 77, no. 10 (2018): 1524–1534. 29991473 10.1136/annrheumdis-2018-213450

[250] A. B. Blom , P. L. van Lent , A. E. Holthuysen , et al., “Synovial Lining Macrophages Mediate Osteophyte Formation During Experimental Osteoarthritis,” Osteoarthritis and Cartilage 12, no. 8 (2004): 627–635. 15262242 10.1016/j.joca.2004.03.003

[251] A. B. Blom , P. L. van Lent , S. Libregts , et al., “Crucial Role of Macrophages in Matrix Metalloproteinase‐mediated Cartilage Destruction During Experimental Osteoarthritis: Involvement of Matrix Metalloproteinase 3,” Arthritis and Rheumatism 56, no. 1 (2007): 147–157. 17195217 10.1002/art.22337

[252] C. L. Wu , J. McNeill , K. Goon , et al., “Conditional Macrophage Depletion Increases Inflammation and Does Not Inhibit the Development of Osteoarthritis in Obese Macrophage Fas‐Induced Apoptosis‐Transgenic Mice,” Arthritis & Rheumatology 69, no. 9 (2017): 1772–1783. 28544542 10.1002/art.40161 PMC5611814

[253] B. Liu , M. Zhang , J. Zhao , M. Zheng , and H. Yang , “Imbalance of M1/M2 Macrophages Is Linked to Severity Level of Knee Osteoarthritis,” Experimental and Therapeutic Medicine 16, no. 6 (2018): 5009–5014. 30546406 10.3892/etm.2018.6852 PMC6256852

[254] A. Damerau , E. Rosenow , D. Alkhoury , F. Buttgereit , and T. Gaber , “Fibrotic Pathways and Fibroblast‐Like Synoviocyte Phenotypes in Osteoarthritis,” Frontiers in Immunology 15 (2024): 1385006. 38895122 10.3389/fimmu.2024.1385006 PMC11183113

[255] D. Han , Y. Fang , X. Tan , et al., “The Emerging Role of Fibroblast‐Like Synoviocytes‐mediated Synovitis in Osteoarthritis: An Update,” Journal of Cellular and Molecular Medicine 24, no. 17 (2020): 9518–9532. 32686306 10.1111/jcmm.15669 PMC7520283

[256] A. Koskinen , K. Vuolteenaho , T. Moilanen , and E. Moilanen , “Resistin as a Factor in Osteoarthritis: Synovial Fluid Resistin Concentrations Correlate Positively With Interleukin 6 and Matrix Metalloproteinases MMP‐1 and MMP‐3,” Scandinavian Journal of Rheumatology 43, no. 3 (2014): 249–253. 24780007 10.3109/03009742.2013.853096

[257] S. Kemble and A. P. Croft , “Critical Role of Synovial Tissue‐Resident Macrophage and Fibroblast Subsets in the Persistence of Joint Inflammation,” Frontiers in Immunology 12 (2021): 715894. 34539648 10.3389/fimmu.2021.715894 PMC8446662

[258] F. Zhang , K. Wei , K. Slowikowski , et al., “Defining Inflammatory Cell States in Rheumatoid Arthritis Joint Synovial Tissues by Integrating Single‐cell Transcriptomics and Mass Cytometry,” Nature Immunology 20, no. 7 (2019): 928–942. 31061532 10.1038/s41590-019-0378-1 PMC6602051

[259] T. W. Kragstrup , D. H. Sohn , C. M. Lepus , et al., “Fibroblast‐Like Synovial Cell Production of Extra Domain A Fibronectin Associates With Inflammation in Osteoarthritis,” BMC Rheumatology 3 (2019): 46. 31819923 10.1186/s41927-019-0093-4 PMC6886182

[260] N. Xie , Z. Tan , S. Banerjee , et al., “Glycolytic Reprogramming in Myofibroblast Differentiation and Lung Fibrosis,” American Journal of Respiratory and Critical Care Medicine 192, no. 12 (2015): 1462–1474. 26284610 10.1164/rccm.201504-0780OC PMC4731722

[261] L. Zheng , Z. Zhang , P. Sheng , and A. Mobasheri , “The Role of Metabolism in Chondrocyte Dysfunction and the Progression of Osteoarthritis,” Ageing Research Reviews 66 (2021): 101249. 33383189 10.1016/j.arr.2020.101249

[262] F. Mizoguchi , K. Slowikowski , K. Wei , et al., “Functionally Distinct Disease‐associated Fibroblast Subsets in Rheumatoid Arthritis,” Nature Communications 9, no. 1 (2018): 789. 10.1038/s41467-018-02892-y PMC5824882 29476097

[263] J. Massague and D. Sheppard , “TGF‐beta Signaling in Health and Disease,” Cell 186, no. 19 (2023): 4007–4037. 37714133 10.1016/j.cell.2023.07.036 PMC10772989

[264] S. Pacquelet , N. Presle , C. Boileau , et al., “Interleukin 17, a Nitric Oxide‐producing Cytokine With a Peroxynitrite‐independent Inhibitory Effect on Proteoglycan Synthesis,” Journal of Rheumatology 29, no. 12 (2002): 2602–2610. 12465160

[265] W. Sun , X. Li , L. Zhang , et al., “IL‐17A Exacerbates Synovial Inflammation in Osteoarthritis via Activation of Endoplasmic Reticulum Stress,” International Immunopharmacology 145 (2025): 113733. 39662267 10.1016/j.intimp.2024.113733

[266] D. Sinkeviciute , A. Aspberg , Y. He , A. C. Bay‐Jensen , and P. Onnerfjord , “Characterization of the Interleukin‐17 Effect on Articular Cartilage in a Translational Model: An Explorative Study,” BMC Rheumatology 4 (2020): 30. 32426694 10.1186/s41927-020-00122-x PMC7216541

[267] E. Wisniewska , D. Laue , J. Spinnen , et al., “Infrapatellar Fat Pad Modulates Osteoarthritis‐Associated Cytokine and MMP Expression in Human Articular Chondrocytes,” Cells 12, no. 24 (2023): 2850. 38132170 10.3390/cells12242850 PMC10741519

[268] J. Apinun , P. Sengprasert , P. Yuktanandana , S. Ngarmukos , A. Tanavalee , and R. Reantragoon , “Immune Mediators in Osteoarthritis: Infrapatellar Fat Pad‐Infiltrating CD8+ T Cells Are Increased in Osteoarthritic Patients With Higher Clinical Radiographic Grading,” International Journal of Rheumatology 2016 (2016): 9525724. 28070192 10.1155/2016/9525724 PMC5192329

[269] N. Zapata‐Linares , L. Loisay , D. de Haro , et al., “Systemic and Joint Adipose Tissue Lipids and Their Role in Osteoarthritis,” Biochimie 227, no. Pt B (2024): 130–138. 39343353 10.1016/j.biochi.2024.09.015

[270] H. Platzer , R. Trauth , T. A. Nees , et al., “CD8(+) T Cells in OA Knee Joints Are Differentiated Into Subsets Depending on OA Stage and Compartment,” Journal of Clinical Medicine 11, no. 10 (2022): 2814. 35628940 10.3390/jcm11102814 PMC9145354

[271] M. A. Boutet , A. Nerviani , L. Fossati‐Jimack , et al., “Comparative Analysis of Late‐stage Rheumatoid Arthritis and Osteoarthritis Reveals Shared Histopathological Features,” Osteoarthritis and Cartilage 32, no. 2 (2024): 166–176. 37984558 10.1016/j.joca.2023.10.009

[272] Z. Wen , L. Qiu , Z. Ye , et al., “The Role of Th/Treg Immune Cells in Osteoarthritis,” Frontiers in Immunology 15 (2024): 1393418. 39364408 10.3389/fimmu.2024.1393418 PMC11446774

[273] L. E. Keller , E. D. Tait Wojno , L. Begum , and L. A. Fortier , “Regulatory T Cells Provide Chondroprotection Through Increased TIMP1, IL‐10 and IL‐4, but CannotNot Mitigate the Catabolic Effects of IL‐1beta and IL‐6 in a Tri‐culture Model of Osteoarthritis,” Osteoarthritis and Cartilage Open 3, no. 3 (2021): 100193. 36474817 10.1016/j.ocarto.2021.100193 PMC9718146

[274] N. Rosshirt , R. Trauth , H. Platzer , et al., “Proinflammatory T Cell Polarization Is Already Present in Patients With Early Knee Osteoarthritis,” Arthritis Research & Therapy 23, no. 1 (2021): 37. 33482899 10.1186/s13075-020-02410-w PMC7821658

[275] H. Luo , Y. Zhu , B. Guo , et al., “Causal Relationships Between CD25 on Immune Cells and Hip Osteoarthritis,” Frontiers in Immunology 14 (2023): 1247710. 37731506 10.3389/fimmu.2023.1247710 PMC10507251

[276] T. A. Nees , J. A. Zhang , H. Platzer , et al., “Infiltration Profile of Regulatory T Cells in Osteoarthritis‐Related Pain and Disability,” Biomedicines 10, no. 9 (2022): 2111. 36140212 10.3390/biomedicines10092111 PMC9495462

[277] S. Li , J. Wan , W. Anderson , H. Sun , H. Zhang , and X. Peng , “Downregulation of IL‐10 Secretion by Treg Cells in Osteoarthritis Is Associated With a Reduction in Tim‐3 Expression,” Biomedicine & Pharmacotherapy 79 (2016): 159–165. 27044824 10.1016/j.biopha.2016.01.036

[278] C. Xu , S. Wang , X. Chen , et al., “Causal Associations Between Circulating Immune Cells and Osteoarthritis: A Bidirectional Mendelian Randomization Study,” International Immunopharmacology 142, no. Pt A (2024): 113156. 39278062 10.1016/j.intimp.2024.113156

[279] M. Atabaki , Z. Shariati‐Sarabi , J. Tavakkol‐Afshari , and M. Mohammadi , “Significant Immunomodulatory Properties of Curcumin in Patients With Osteoarthritis; a Successful Clinical Trial in Iran,” International Immunopharmacology 85 (2020): 106607. 32540725 10.1016/j.intimp.2020.106607

[280] H. Sun , Y. Zhang , W. Song , et al., “IgM(+)CD27(+) B Cells Possessed Regulatory Function and Represented the Main Source of B Cell‐derived IL‐10 in the Synovial Fluid of Osteoarthritis Patients,” Human Immunology 80, no. 4 (2019): 263–269. 30769033 10.1016/j.humimm.2019.02.007

[281] D. M. Black and C. J. Rosen , “Clinical Practice. Postmenopausal Osteoporosis,” New England Journal of Medicine 374, no. 3 (2016): 254–262. 26789873 10.1056/NEJMcp1513724

[282] Y. W. Zhang , M. M. Cao , Y. J. Li , et al., “Dietary Protein Intake in Relation to the Risk of Osteoporosis in Middle‐Aged and Older Individuals: A Cross‐Sectional Study,” Journal of Nutrition, Health and Aging 26, no. 3 (2022): 252–258. 10.1007/s12603-022-1748-1 PMC12275630 35297468

[283] L. Sapra , C. Saini , P. K. Mishra , et al., “Bacillus Coagulans Ameliorates Inflammatory Bone Loss in Post‐menopausal Osteoporosis via Modulating the "Gut‐Immune‐Bone" Axis,” Gut Microbes 17, no. 1 (2025): 2492378. 40275534 10.1080/19490976.2025.2492378 PMC12036487

[284] S. Song , Y. Guo , Y. Yang , and D. Fu , “Advances in Pathogenesis and Therapeutic Strategies for Osteoporosis,” Pharmacology & Therapeutics 237 (2022): 108168. 35283172 10.1016/j.pharmthera.2022.108168

[285] J. Munoz , N. S. Akhavan , A. P. Mullins , and B. H. Arjmandi , “Macrophage Polarization and Osteoporosis: A Review,” Nutrients 12, no. 10 (2020): 2999. 33007863 10.3390/nu12102999 PMC7601854

[286] J. Tsay , Z. Yang , F. P. Ross , et al., “Bone Loss Caused by Iron Overload in a Murine Model: Importance of Oxidative Stress,” Blood 116, no. 14 (2010): 2582–2589. 20554970 10.1182/blood-2009-12-260083 PMC2953890

[287] J. L. Kirkland and T. Tchkonia , “Senolytic Drugs: From Discovery to Translation,” Journal of Internal Medicine 288, no. 5 (2020): 518–536. 32686219 10.1111/joim.13141 PMC7405395

[288] Y. Sun , J. Li , X. Xie , et al., “Macrophage‐Osteoclast Associations: Origin, Polarization, and Subgroups,” Frontiers in Immunology 12 (2021): 778078. 34925351 10.3389/fimmu.2021.778078 PMC8672114

[289] R. Pacifici , “Role of Gut Microbiota in the Skeletal Response to PTH,” Journal of Clinical Endocrinology and Metabolism 106, no. 3 (2021): 636–645. 33254225 10.1210/clinem/dgaa895 PMC7947780

[290] M. Yu , A. Malik Tyagi , J. Y. Li , J. Adams , T. L. Denning , and M. N. Weitzmann , “PTH Induces Bone Loss via Microbial‐dependent Expansion of Intestinal TNF(+) T Cells and Th17 Cells,” Nature Communications 11, no. 1 (2020): 468. 10.1038/s41467-019-14148-4 PMC6981196 31980603

[291] F. An , X. Jia , Y. Shi , X. Xiao , F. Yang , and J. Su , “The Ultimate Microbial Composition for Correcting Th17/Treg Cell Imbalance and Lipid Metabolism Disorders in Osteoporosis,” International Immunopharmacology 144 (2025): 113613. 39571271 10.1016/j.intimp.2024.113613

[292] W. Zhang , W. Zhao , W. Li , et al., “The Imbalance of Cytokines and Lower Levels of Tregs in Elderly Male Primary Osteoporosis,” Frontiers in Endocrinolog (Lausanne) 13 (2022): 779264. 10.3389/fendo.2022.779264 PMC9205399 35721756

[293] X. Yang , F. Zhou , P. Yuan , et al., “T Cell‐depleting Nanoparticles Ameliorate Bone Loss by Reducing Activated T Cells and Regulating the Treg/Th17 Balance,” Bioactive Materials 6, no. 10 (2021): 3150–3163. 33778195 10.1016/j.bioactmat.2021.02.034 PMC7970013

[294] F. Huang , P. Wong , J. Li , et al., “Osteoimmunology: The Correlation Between Osteoclasts and the Th17/Treg Balance in Osteoporosis,” Journal of Cellular and Molecular Medicine 26, no. 13 (2022): 3591–3597. 35633138 10.1111/jcmm.17399 PMC9258696

[295] A. Taylor , J. Verhagen , K. Blaser , M. Akdis , and C. A. Akdis , “Mechanisms of Immune Suppression by Interleukin‐10 and Transforming Growth Factor‐beta: The Role of T Regulatory Cells,” Immunology 117, no. 4 (2006): 433–442. 16556256 10.1111/j.1365-2567.2006.02321.x PMC1782242

[296] C. F. Francisconi , A. E. Vieira , M. C. S. Azevedo , et al., “RANKL Triggers Treg‐Mediated Immunoregulation in Inflammatory Osteolysis,” Journal of Dental Research 97, no. 8 (2018): 917–927. 29499125 10.1177/0022034518759302 PMC6728554

[297] L. Sapra , H. Y. Dar , A. Bhardwaj , et al., “Lactobacillus Rhamnosus Attenuates Bone Loss and Maintains Bone Health by Skewing Treg‐Th17 Cell Balance in Ovx Mice,” Scientific Reports 11, no. 1 (2021): 1807. 33469043 10.1038/s41598-020-80536-2 PMC7815799

[298] H. Y. Dar , S. Pal , P. Shukla , et al., “Bacillus Clausii Inhibits Bone Loss by Skewing Treg‐Th17 Cell Equilibrium in Postmenopausal Osteoporotic Mice Model,” Nutrition (Burbank, Los Angeles County, Calif) 54 (2018): 118–128. 29793054 10.1016/j.nut.2018.02.013

[299] J. Piao , J. S. Park , D. Y. Hwang , Y. Son , and H. S. Hong , “Substance P Blocks Ovariectomy‐induced Bone Loss by Modulating Inflammation and Potentiating Stem Cell Function,” Aging (Albany NY) 12, no. 20 (2020): 20753–20777. 33109775 10.18632/aging.104008 PMC7655156

[300] L. Sapra , A. Bhardwaj , P. K. Mishra , et al., “Regulatory B Cells (Bregs) Inhibit Osteoclastogenesis and Play a Potential Role in Ameliorating Ovariectomy‐Induced Bone Loss,” Frontiers in Immunology 12 (2021): 691081. 34276682 10.3389/fimmu.2021.691081 PMC8278221

[301] D. Frase , C. Lee , C. Nachiappan , R. Gupta , and A. Akkouch , “The Inflammatory Contribution of B‐Lymphocytes and Neutrophils in Progression to Osteoporosis,” Cells 12, no. 13 (2023): 1744. 37443778 10.3390/cells12131744 PMC10340451

[302] L. Sapra , C. Saini , P. K. Mishra , B. Garg , M. Gupta , and R. K. Srivastava , “Compromised Anti‐osteoclastogenic and Immunomodulatory Functions of Regulatory B Cells (Bregs) Aggravate Inflammatory Bone Loss in Post‐menopausal Osteoporosis,” Biochimica et Biophysica Acta: Molecular Basis of Disease 1871, no. 3 (2025): 167675. 39826852 10.1016/j.bbadis.2025.167675

[303] Y. Wang , W. Zhang , S. M. Lim , L. Xu , and J. O. Jin , “Interleukin‐10‐Producing B Cells Help Suppress Ovariectomy‐Mediated Osteoporosis,” Immune Network 20, no. 6 (2020): e50. 33425435 10.4110/in.2020.20.e50 PMC7779870

[304] L. Sapra , N. Shokeen , K. Porwal , et al., “Bifidobacterium Longum Ameliorates Ovariectomy‐Induced Bone Loss via Enhancing Anti‐Osteoclastogenic and Immunomodulatory Potential of Regulatory B Cells (Bregs),” Frontiers in Immunology 13 (2022): 875788. 35693779 10.3389/fimmu.2022.875788 PMC9174515

[305] X. Chen , A. Zhang , K. Zhao , et al., “The Role of Oxidative Stress in Intervertebral Disc Degeneration: Mechanisms and Therapeutic Implications,” Ageing Research Reviews 98 (2024): 102323. 38734147 10.1016/j.arr.2024.102323

[306] L. Kang , H. Zhang , C. Jia , R. Zhang , and C. Shen , “Epigenetic Modifications of Inflammation in Intervertebral Disc Degeneration,” Ageing Research Reviews 87 (2023): 101902. 36871778 10.1016/j.arr.2023.101902

[307] X. Kong , H. Gu , Y. Zhang , et al., “beta‐Mangostin Attenuates TET2‐Mediated DNA Demethylation of Prkcg in the Prevention of Intervertebral Disc Degeneration,” Advanced science (Weinheim) 12, no. 32 (2025): e05077. 10.1002/advs.202505077 PMC12407310 40558107

[308] Y. Huang , H. Li , L. Qi , et al., “NanoCRISPR‐assisted Biomimetic Tissue‐equivalent Patch Regenerates the Intervertebral Disc by Inhibiting Endothelial‐to‐mesenchymal Transition,” Biomaterials 322 (2025): 123404. 40398216 10.1016/j.biomaterials.2025.123404

[309] K. G. Burt , M. K. M. Kim , D. C. Viola , A. C. Abraham , and N. O. Chahine , “Nuclear Factor kappaB Overactivation in the Intervertebral Disc Leads to Macrophage Recruitment and Severe Disc Degeneration,” Science Advances 10, no. 23 (2024): eadj3194. 38848366 10.1126/sciadv.adj3194 PMC11160472

[310] X. Zhao , Z. Sun , B. Xu , et al., “Degenerated Nucleus Pulposus Cells Derived Exosome Carrying miR‐27a‐3p Aggravates Intervertebral Disc Degeneration by Inducing M1 Polarization of Macrophages,” Journal of Nanobiotechnology 21, no. 1 (2023): 317. 37667246 10.1186/s12951-023-02075-y PMC10478255

[311] Y. Fu , H. Sun , Y. Jin , S. Cheng , Y. Wu , and C. Liu , “Self‐assembled Antioxidant Enzyme‐mimicking Hydrogel: Targeting Oxidative Stress and Macrophage Organization for Improving Degenerated Intervertebral Discs,” Materials Today Bio 31 (2025): 101586. 10.1016/j.mtbio.2025.101586 PMC11923825 40115052

[312] Y. Dou , Y. Zhang , Y. Liu , et al., “Role of Macrophage in Intervertebral Disc Degeneration,” Bone Research 13, no. 1 (2025): 15. 39848963 10.1038/s41413-024-00397-7 PMC11758090

[313] X. C. Li , S. J. Luo , W. Fan , T. L. Zhou , D. Q. Tan , and R. X. Tan , “Macrophage Polarization Regulates Intervertebral Disc Degeneration by Modulating Cell Proliferation, Inflammation Mediator Secretion, and Extracellular Matrix Metabolism,” Frontiers in Immunology 13 (2022): 922173. 36059551 10.3389/fimmu.2022.922173 PMC9433570

[314] F. Li , Y. Shi , J. Chen , et al., “LGR6 modulates Intervertebral Disc Degeneration Through Regulation of Macrophage Efferocytosis,” Journal of Translational Medicine 23, no. 1 (2025): 475. 40281518 10.1186/s12967-025-06427-0 PMC12023656

[315] X. J. Yu , P. Zou , T. Q. Li , et al., “Deciphering SPP1‐related Macrophage Signaling in the Pathogenesis of Intervertebral Disc Degeneration,” Cell Biology and Toxicology 41, no. 1 (2025): 33. 39825191 10.1007/s10565-024-09948-4 PMC11748470

[316] Y. Zhang , J. Zhang , Z. Sun , et al., “MAPK8 and CAPN1 as Potential Biomarkers of Intervertebral Disc Degeneration Overlapping Immune Infiltration, Autophagy, and ceRNA,” Frontiers in Immunology 14 (2023): 1188774. 37325630 10.3389/fimmu.2023.1188774 PMC10266224

[317] S. W. Clayton , R. E. Walk , L. Mpofu , G. W. D. Easson , and S. Y. Tang , “Sex‐specific Divergences in the Types and Timing of Infiltrating Immune Cells During the Intervertebral Disc Acute Injury Response and Their Associations With Degeneration,” Osteoarthritis and Cartilage 33, no. 2 (2025): 247–260. 39426787 10.1016/j.joca.2024.10.002 PMC12525793

[318] C. Xu , M. Zhang , K. Li , et al., “CD24(hi)CD38(hi) B Regulatory Cells From Patients With End Plate Inflammation Presented Reduced Functional Potency,” International Immunopharmacology 70 (2019): 295–301. 30851710 10.1016/j.intimp.2019.02.034

[319] K. Guo , J. Zeng , J. Lu , et al., “The Clinical Significance of the Neutrophil‐to‐Lymphocyte Ratio as a Novel Inflammatory Biomarker for Assessing the Severity of Intervertebral Disc Degeneration,” Frontiers in Medicine (Lausanne) 11 (2024): 1446124. 10.3389/fmed.2024.1446124 PMC11560784 39544385

[320] T. L. Zhang , W. K. Chen , X. P. Huang , et al., “Single‐cell RNA Sequencing Reveals the MIF/ACKR3 Receptor‐ligand Interaction Between Neutrophils and Nucleus Pulposus Cells in Intervertebral Disc Degeneration,” Translational Research 272 (2024): 1–18. 38823438 10.1016/j.trsl.2024.05.011

[321] B. Song , J. Wang , H. Tang , H. Li , and W. Zhang , “Shared Diagnostic Genes and Potential Mechanisms Between Intervertebral Disc Degeneration and Diabetes Mellitus Revealed by Integrated Transcriptomic Analysis and Machine Learning,” Frontiers in Endocrinology (Lausanne) 16 (2025): 1576826. 10.3389/fendo.2025.1576826 PMC12313482 40756519

[322] W. Li , Z. Ding , H. Zhang , et al., “The Roles of Blood Lipid‐Metabolism Genes in Immune Infiltration Could Promote the Development of IDD,” Frontiers in Cell and Developmental Biology 10 (2022): 844395. 35223859 10.3389/fcell.2022.844395 PMC8864150

[323] T. Shao , Q. Gao , W. Tang , Y. Ma , J. Gu , and Z. Yu , “The Role of Immunocyte Infiltration Regulatory Network Based on hdWGCNA and Single‐Cell Bioinformatics Analysis in Intervertebral Disc Degeneration,” Inflammation 47, no. 6 (2024): 1987–1999. 38630169 10.1007/s10753-024-02020-7

[324] Y. Yamamoto , Y. Kokubo , H. Nakajima , K. Honjoh , S. Watanabe , and A. Matsumine , “Distribution and Polarization of Hematogenous Macrophages Associated With the Progression of Intervertebral Disc Degeneration,” Spine (Phila Pa 1976) 47, no. 4 (2022): E149–E158. 34545043 10.1097/BRS.0000000000004222

[325] X. C. Li , W. Wang , C. Jiang , et al., “CD206(+) M2‐Like Macrophages Protect Against Intervertebral Disc Degeneration Partially by Targeting R‐spondin‐2,” Osteoarthritis and Cartilage 32, no. 1 (2024): 66–81. 37802465 10.1016/j.joca.2023.09.010

[326] Z. Tao , T. Zhang , Y. Ge , et al., “M2 macrophages Regulate Nucleus Pulposus Cell Extracellular Matrix Synthesis Through the OPN‐CD44 Axis in Intervertebral Disc Degeneration,” Osteoarthritis and Cartilage 33, no. 4 (2025): 447–460. 39842659 10.1016/j.joca.2024.12.007

[327] X. C. Li , S. J. Luo , W. Fan , T. L. Zhou , C. M. Huang , and M. S. Wang , “M2 macrophage‐conditioned Medium Inhibits Intervertebral Disc Degeneration in a Tumor Necrosis Factor‐alpha‐rich Environment,” Journal of Orthopaedic Research 40, no. 11 (2022): 2488–2501. 35170802 10.1002/jor.25292

[328] Y. Liu , M. Xue , Y. Han , Y. Li , B. Xiao , and W. Wang , “Exosomes From M2c Macrophages Alleviate Intervertebral Disc Degeneration by Promoting Synthesis of the Extracellular Matrix via MiR‐124/CILP/TGF‐beta,” Bioengineering & Translational Medicine 8, no. 6 (2023): e10500. 38023721 10.1002/btm2.10500 PMC10658595

[329] K. Zhang , L. Du , Z. Li , et al., “M2 Macrophage‐Derived Small Extracellular Vesicles Ameliorate Pyroptosis and Intervertebral Disc Degeneration,” Biomaterials Research 28 (2024): 0047. 38952714 10.34133/bmr.0047 PMC11214826

[330] S. P. Zhang , M. Tong , J. Mo , Z. Y. Dong , and Y. F. Huang , “M2 macrophages Activate the IL‐10/JAK2/STAT3 Pathway to Induce Pathological Microangiogenesis in the Nucleus Pulposus Exacerbating Intervertebral Disc Degeneration,” Journal of Orthopaedic Surgery and Research 20, no. 1 (2025): 532. 40426248 10.1186/s13018-025-05962-2 PMC12117970

[331] J. Qian , X. Wang , G. Su , et al., “Platelet‐rich Plasma‐derived Exosomes Attenuate Intervertebral Disc Degeneration by Promoting NLRP3 Autophagic Degradation in Macrophages,” International Immunopharmacology 110 (2022): 108962. 35753124 10.1016/j.intimp.2022.108962

[332] J. Luo , G. Jin , S. Cui , H. Wang , and Q. Liu , “Regulatory Mechanism of FCGR2A in Macrophage Polarization and Its Effects on Intervertebral Disc Degeneration,” The Journal of Physiology 602, no. 7 (2024): 1341–1369. 38544414 10.1113/JP285871

[333] J. Tu , W. Li , S. Yang , et al., “Single‐Cell Transcriptome Profiling Reveals Multicellular Ecosystem of Nucleus Pulposus During Degeneration Progression,” Advanced science (Weinheim) 9, no. 3 (2022): e2103631. 10.1002/advs.202103631 PMC8787427 34825784

[334] C. Zhang , H. Li , H. Wang , et al., “Identifying Myeloid‐Derived Suppressor Cells and Lipocalin‐2 as Therapeutic Targets for Intervertebral Disc Degeneration,” Advanced Science (Weinheim) 12, no. 34 (2025): e00505. 10.1002/advs.202500505 PMC12442675 40570207

[335] H. Zhou , C. Liu , F. Hu , et al., “Increased Levels of Circulating Granulocytic Myeloid‑Derived Suppressor Cells in Lumbar Disc Herniation,” Experimental and Therapeutic Medicine 26, no. 2 (2023): 367. 37408862 10.3892/etm.2023.12066 PMC10318602

[336] A. J. Cruz‐Jentoft and A. A. Sayer , “Sarcopenia,” Lancet 393, no. 10191 (2019): 2636–2646. 31171417 10.1016/S0140-6736(19)31138-9

[337] A. A. Sayer , R. Cooper , H. Arai , P. M. Cawthon , M. J. Ntsama Essomba , and R. A. Fielding , “Sarcopenia,” Nature Reviews Disease Primers 10, no. 1 (2024): 68. 10.1038/s41572-024-00550-w 39300120

[338] X. Zhang , H. Li , M. He , J. Wang , Y. Wu , and Y. Li , “Immune System and Sarcopenia: Presented Relationship and Future Perspective,” Experimental Gerontology 164 (2022): 111823. 35504482 10.1016/j.exger.2022.111823

[339] S. J. Heo and Y. S. Jee , “Characteristics of Age Classification Into Five‐Year Intervals to Explain Sarcopenia and Immune Cells in Older Adults,” Medicina (Kaunas, Lithuania) 59, no. 10 (2023): 1700. 37893417 10.3390/medicina59101700 PMC10607932

[340] S. Doi , S. Yasuda , M. Miyashita , et al., “Prognostic Relevance of Sarcopenia and Tumor‐infiltrating CD8(+) T Cells in Patients With Hepatocellular Carcinoma,” Annals of Gastroenterological Surgery 9, no. 2 (2025): 359–368. 40046516 10.1002/ags3.12875 PMC11877349

[341] S. Masuda , K. Yamakawa , A. Masuda , et al., “Association of Sarcopenia With a Poor Prognosis and Decreased Tumor‐Infiltrating CD8‐Positive T Cells in Pancreatic Ductal Adenocarcinoma: A Retrospective Analysis,” Annals of Surgical Oncology 30, no. 9 (2023): 5776–5787. 37191859 10.1245/s10434-023-13569-2 PMC10409680

[342] S. L. Ma , J. Wu , L. Zhu , et al., “Peripheral Blood T Cell Gene Expression Responses to Exercise and HMB in Sarcopenia,” Nutrients 13, no. 7 (2021): 2313. 34371826 10.3390/nu13072313 PMC8308783

[343] S. W. Huang , T. Xu , C. T. Zhang , and H. L. Zhou , “Relationship of Peripheral Lymphocyte Subsets and Skeletal Muscle Mass Index in Sarcopenia: A Cross‐Sectional Study,” Journal of Nutrition, Health and Aging 24, no. 3 (2020): 325–329.10.1007/s12603-020-1329-032115615

[344] A. Granic , C. Martin‐Ruiz , R. M. Dodds , et al., “Immunosenescence Profiles Are Not Associated With Muscle Strength, Physical Performance and Sarcopenia Risk in Very Old Adults: The Newcastle 85+ Study,” Mechanisms of Ageing and Development 190 (2020): 111321. 32735896 10.1016/j.mad.2020.111321

[345] L. Shen , Y. Zong , J. Zhao , et al., “Characterizing the Skeletal Muscle Immune Microenvironment for Sarcopenia: Insights From Transcriptome Analysis and Histological Validation,” Frontiers in Immunology 15 (2024): 1414387. 39026669 10.3389/fimmu.2024.1414387 PMC11254692

[346] H. J. Oh , H. Jin , and B. Y. Lee , “Hesperidin Ameliorates Sarcopenia Through the Regulation of Inflammaging and the AKT/mTOR/FoxO3a Signaling Pathway in 22‐26‐Month‐Old Mice,” Cells 12, no. 15 (2023): 2015. 37566094 10.3390/cells12152015 PMC10417333

[347] G. Song , H. J. Oh , H. Jin , H. Han , and B. Y. Lee , “GABA Prevents Sarcopenia by Regulation of Muscle Protein Degradation and Inflammaging in 23‐ to 25‐Month‐Old Female Mice,” Journal of Cachexia, Sarcopenia and Muscle 15, no. 6 (2024): 2852–2864. 39513373 10.1002/jcsm.13646 PMC11634462

[348] E. Alcalde‐Estevez , A. Moreno‐Piedra , A. Asenjo‐Bueno , et al., “Aging‐related Hyperphosphatemia Triggers the Release of TNF‐alpha From Macrophages, Promoting Indicators of Sarcopenia Through the Reduction of IL‐15 Expression in Skeletal Muscle,” Life Sciences 368 (2025): 123507. 40010633 10.1016/j.lfs.2025.123507

[349] Y. Y. Chen , T. W. Kao , Y. L. Chiu , T. C. Peng , H. F. Yang , and W. L. Chen , “Association between Interleukin‐12 and Sarcopenia,” Journal of Inflammation Research 14 (2021): 2019–2029. 34040414 10.2147/JIR.S313085 PMC8140914

[350] X. Zhang , P. Zhang , Y. Zhu , et al., “Myogenic Nano‐adjuvant for Orthopedic‐related Sarcopenia via Mitochondrial Homeostasis Modulation in Macrophage‐myosatellite Metabolic Crosstalk,” Journal of Nanobiotechnology 23, no. 1 (2025): 390. 40437492 10.1186/s12951-025-03480-1 PMC12117855

[351] Z. Zeng , Z. Zhang , L. Chang , et al., “Therapeutic Silicate Biomaterials for Sarcopenia Treatment by Inhibiting Inflammation and Enhancing Muscle Regeneration Through Regulation of Sarcolipin/SIRT Signaling Pathway,” Bioactive Materials 51 (2025): 787–806. 40809080 10.1016/j.bioactmat.2025.06.040 PMC12348681

[352] P. Ding , H. Wu , T. Li , et al., “Impact of Preoperative Sarcopenia on Postoperative Complications and Prognosis in Patients Undergoing Robotic Gastric Cancer Surgery: A Propensity Score Matching Study,” Nutrition (Burbank, Los Angeles County, Calif) 123 (2024): 112408. 38513525 10.1016/j.nut.2024.112408

[353] L. Xiao , Y. Liu , X. Zhang , et al., “Prognostic Value of Sarcopenia and Inflammatory Indices Synergy in Patients With Esophageal Squamous Cell Carcinoma Undergoing Chemoradiotherapy,” BMC cancer 24, no. 1 (2024): 860. 39026185 10.1186/s12885-024-12602-1 PMC11256500

[354] Y. Zhang , L. Zhang , Y. Guan , et al., “Establishment and Validation of a Risk Prediction Model for Sarcopenia in Gastrointestinal Cancer Patients: A Systematic Review and Meta‐analysis‐based Approach,” Clinical Nutrition 43, no. 11 (2024): 91–98. 39357087 10.1016/j.clnu.2024.08.014

[355] I. Balazs , M. Stelzer , J. Traub , A. Horvath , N. Feldbacher , and V. Stadlbauer , “Primary Sarcopenia Is Associated With Elevated Spontaneous NET Formation,” Frontiers in Cell and Developmental Biology 12 (2024): 1347495. 38505257 10.3389/fcell.2024.1347495 PMC10948394

[356] Y. Wang , L. Wang , Y. Zhang , et al., “Comprehensive Profiling of Chemokine and NETosis‐associated Genes in Sarcopenia: Construction of a Machine Learning‐based Diagnostic Nomogram,” Frontiers in Medicine (Lausanne) 12 (2025): 1606430. 10.3389/fmed.2025.1606430 PMC12230068 40625358

[357] Y. W. Chen , S. He , Y. Wang , L. Y. Hu , Q. K. Chen , and S. Y. Liu , “Mitochondrial Insights: Key Biomarkers and Potential Treatments for Diabetic Nephropathy and Sarcopenia,” Frontiers in Cell and Developmental Biology 13 (2025): 1596204. 40703651 10.3389/fcell.2025.1596204 PMC12283731

[358] Y. He , T. Lin , R. Liang , et al., “Interleukin 25 Promotes Muscle Regeneration in Sarcopenia by Regulating Macrophage‐mediated Sonic Hedgehog Signaling,” International Immunopharmacology 139 (2024): 112662. 39038385 10.1016/j.intimp.2024.112662

[359] Y. Liu , M. Ge , X. Xiao , Y. Lu , W. Zhao , and K. Zheng , “Sarcosine Decreases in Sarcopenia and Enhances Muscle Regeneration and Adipose Thermogenesis by Activating Anti‐inflammatory Macrophages,” Nature Aging 5, no. 9 (2025): 1810–1827. 40550878 10.1038/s43587-025-00900-7

[360] M. Hong , I. H. Han , I. Choi , et al., “Magnoliae Cortex Alleviates Muscle Wasting by Modulating M2 Macrophages in a Cisplatin‐Induced Sarcopenia Mouse Model,” International Journal of Molecular Sciences 22, no. 6 (2021): 3188. 33804803 10.3390/ijms22063188 PMC8003985

[361] Y. Wang , S. S. Welc , M. Wehling‐Henricks , et al., “Myeloid Cell‐specific Mutation of Spi1 Selectively Reduces M2‐biased Macrophage Numbers in Skeletal Muscle, Reduces Age‐related Muscle Fibrosis and Prevents Sarcopenia,” Aging Cell 21, no. 10 (2022): e13690. 36098370 10.1111/acel.13690 PMC9577952

[362] S. Schiaffino , M. G. Pereira , S. Ciciliot , and P. Rovere‐Querini , “Regulatory T Cells and Skeletal Muscle Regeneration,” Febs Journal 284, no. 4 (2017): 517–524. 27479876 10.1111/febs.13827

[363] S. Oh , J. Y. Yang , C. H. Park , K. H. Son , and K. Byun , “Dieckol Reduces Muscle Atrophy by Modulating Angiotensin Type II Type 1 Receptor and NADPH Oxidase in Spontaneously Hypertensive Rats,” Antioxidants (Basel) 10, no. 10 (2021): 1561. 34679696 10.3390/antiox10101561 PMC8533257

[364] Y. Xiang , J. Dai , L. Xu , X. Li , J. Jiang , and J. Xu , “Research Progress in Immune Microenvironment Regulation of Muscle Atrophy Induced by Peripheral Nerve Injury,” Life Sciences 287 (2021): 120117. 34740577 10.1016/j.lfs.2021.120117

[365] A. Narsale , R. Moya , J. Ma , et al., “Cancer‐driven Changes Link T Cell Frequency to Muscle Strength in People With Cancer: A Pilot Study,” Journal of Cachexia, Sarcopenia and Muscle 10, no. 4 (2019): 827–843. 30977974 10.1002/jcsm.12424 PMC6711422

[366] J. Yu , H. Ahn , K. Y. Han , et al., “Paradoxical Effect of Myosteatosis on the Immune Checkpoint Inhibitor Response in Metastatic Renal Cell Carcinoma,” Journal of Cachexia, Sarcopenia and Muscle 16, no. 2 (2025): e13758. 40052383 10.1002/jcsm.13758 PMC11886412

[367] A. Traweger , A. Scott , M. Kjaer , et al., “Achilles Tendinopathy,” Nature Reviews Disease Primers 11, no. 1 (2025): 20. 10.1038/s41572-025-00602-9 40148342

[368] M. T. Cooper , “Common Painful Foot and Ankle Conditions: A Review,” Jama 330, no. 23 (2023): 2285–2294. 38112812 10.1001/jama.2023.23906

[369] M. S. Kragsnaes , U. Fredberg , K. Stribolt , S. G. Kjaer , K. Bendix , and T. Ellingsen , “Stereological Quantification of Immune‐competent Cells in Baseline Biopsy Specimens From Achilles Tendons: Results From Patients With Chronic Tendinopathy Followed for More Than 4 Years,” American Journal of Sports Medicine 42, no. 10 (2014): 2435–2445. 25081311 10.1177/0363546514542329

[370] N. M. Malmgaard‐Clausen , M. Kjaer , and S. G. Dakin , “Pathological Tendon Histology in Early and Chronic Human Patellar Tendinopathy,” Translational Sports Medicine 2022 (2022): 2799665. 38655164 10.1155/2022/2799665 PMC11022758

[371] N. L. Millar , A. J. Hueber , J. H. Reilly , et al., “Inflammation Is Present in Early human Tendinopathy,” American Journal of Sports Medicine 38, no. 10 (2010): 2085–2091. 20595553 10.1177/0363546510372613

[372] N. Cho , S. G. Lee , J. O. Kim , et al., “Identification of Differentially Expressed Genes Associated With Extracellular Matrix Degradation and Inflammatory Regulation in Calcific Tendinopathy Using RNA Sequencing,” Calcified Tissue International 107, no. 5 (2020): 489–498. 32776213 10.1007/s00223-020-00743-x

[373] Z. Chen , M. Li , P. Chen , et al., “Mechanical Overload‐induced Release of Extracellular Mitochondrial Particles From Tendon Cells Leads to Inflammation in Tendinopathy,” Experimental & Molecular Medicine 56, no. 3 (2024): 583–599. 38424192 10.1038/s12276-024-01183-5 PMC10985099

[374] J. Xu , M. Zheng , Z. Feng , and Q. Lin , “CCL4L2 participates in Tendinopathy Progression by Promoting Macrophage Inflammatory Responses: A Single‐cell Analysis,” Journal of Orthopaedic Surgery and Research 19, no. 1 (2024): 836. 39696421 10.1186/s13018-024-05268-9 PMC11656782

[375] J. Herman , B. Le Goff , J. De Lima , R. Brion , C. Chevalier , and F. Blanchard , “Pro‐inflammatory Effects of human Apatite Crystals Extracted From Patients Suffering From Calcific Tendinopathy,” Arthritis Research & Therapy 23, no. 1 (2021): 131. 33926523 10.1186/s13075-021-02516-9 PMC8082912

[376] N. L. Millar , M. Akbar , A. L. Campbell , et al., “IL‐17A Mediates Inflammatory and Tissue Remodelling Events in Early human Tendinopathy,” Scientific Reports 6 (2016): 27149. 27263531 10.1038/srep27149 PMC4893609

[377] H. Jiang , Y. Xie , J. Lu , et al., “Pristimerin Suppresses AIM2 Inflammasome by Modulating AIM2‐PYCARD/ASC Stability via Selective Autophagy to Alleviate Tendinopathy,” Autophagy 20, no. 1 (2024): 76–93. 37647255 10.1080/15548627.2023.2249392 PMC10761048

[378] J. Christensen , H. Alfredson , and G. Andersson , “Protease‐activated Receptors in the Achilles Tendon‐a Potential Explanation for the Excessive Pain Signalling in Tendinopathy,” Molecular Pain 11 (2015): 13. 25880199 10.1186/s12990-015-0007-4 PMC4369088

[379] R. Gao , T. Ye , Z. Zhu , et al., “Small Extracellular Vesicles From iPSC‐derived Mesenchymal Stem Cells Ameliorate Tendinopathy Pain by Inhibiting Mast Cell Activation,” Nanomedicine (London) 17, no. 8 (2022): 513–529. 10.2217/nnm-2022-0036 35289187

[380] H. Behzad , A. Sharma , R. Mousavizadeh , A. Lu , and A. Scott , “Mast Cells Exert Pro‐inflammatory Effects of Relevance to the Pathophyisology of Tendinopathy,” Arthritis Research & Therapy 15, no. 6 (2013): R184. 24517261 10.1186/ar4374 PMC3978883

[381] Y. Wang , J. Zhang , Y. Lin , et al., “A Global Phosphorylation Atlas of Proteins within Pathological Site of Rotator Cuff Tendinopathy,” Frontiers in Molecular Biosciences 8 (2021): 787008. 35242811 10.3389/fmolb.2021.787008 PMC8886731

[382] Y. T. Wu , Y. T. Wu , T. C. Huang , F. C. Su , I. M. Jou , and C. C. Wu , “Sequential Inflammation Model for Achilles Tendinopathy by Elastin Degradation With Treadmill Exercise,” Journal of Orthopaedic Translation 23 (2020): 113–121. 32642426 10.1016/j.jot.2020.03.004 PMC7322491

[383] D. Kouroupis , C. Perucca Orfei , D. Correa , G. Talo , F. Libonati , and P. De Luca , “Cellular and Structural Changes in Achilles and Patellar Tendinopathies: A Pilot in Vivo Study,” Biomedicines 12, no. 5 (2024): 995. 38790957 10.3390/biomedicines12050995 PMC11117798

[384] W. Fu , R. Yang , and J. Li , “Single‐cell and Spatial Transcriptomics Reveal Changes in Cell Heterogeneity During Progression of human Tendinopathy,” BMC Biology 21, no. 1 (2023): 132. 37280595 10.1186/s12915-023-01613-2 PMC10246392

[385] R. Chen , L. Ai , J. Zhang , and D. Jiang , “Dendritic Cell‐Derived Exosomes Promote Tendon Healing and Regulate Macrophage Polarization in Preventing Tendinopathy,” International Journal of Nanomedicine 19 (2024): 11701–11718. 39558915 10.2147/IJN.S466363 PMC11571930

[386] C. Wang , Y. Zhang , G. Zhang , W. Yu , and Y. He , “Adipose Stem Cell‐Derived Exosomes Ameliorate Chronic Rotator Cuff Tendinopathy by Regulating Macrophage Polarization: From a Mouse Model to a Study in Human Tissue,” American Journal of Sports Medicine 49, no. 9 (2021): 2321–2331. 34259608 10.1177/03635465211020010

[387] G. Wu , Q. Su , J. Li , et al., “NAMPT Encapsulated by Extracellular Vesicles From Young Adipose‐derived Mesenchymal Stem Cells Treated Tendinopathy in a "One‐Stone‐Two‐Birds" Manner,” Journal of Nanobiotechnology 21, no. 1 (2023): 7. 36604715 10.1186/s12951-022-01763-5 PMC9814467

[388] T. Ye , Z. Chen , J. Zhang , et al., “Large Extracellular Vesicles Secreted by human iPSC‐derived MSCs Ameliorate Tendinopathy via Regulating Macrophage Heterogeneity,” Bioactive Materials 21 (2023): 194–208. 36101856 10.1016/j.bioactmat.2022.08.007 PMC9440485

[389] D. Li , S. Li , S. He , et al., “Restoring Tendon Microenvironment in Tendinopathy: Macrophage Modulation and Tendon Regeneration With Injectable Tendon Hydrogel and Tendon‐derived Stem Cells Exosomes,” Bioactive Materials 47 (2025): 152–169. 39906648 10.1016/j.bioactmat.2025.01.016 PMC11791013

[390] S. Lopez‐Cerda , G. Molinaro , R. P. Tello , et al., “Study of the Synergistic Immunomodulatory and Antifibrotic Effects of Dual‐Loaded Budesonide and Serpine1 siRNA Lipid‐Polymer Nanoparticles Targeting Macrophage Dysregulation in Tendinopathy,” ACS Applied Materials and Interfaces 16, no. 15 (2024): 18643–18657. 38564504 10.1021/acsami.4c02363 PMC11040533

[391] J. Espejo Valle‐Inclan , S. De Noon , K. Trevers , H. Elrick , I. van Belzen , and S. Zumalave , “Ongoing Chromothripsis Underpins Osteosarcoma Genome Complexity and Clonal Evolution,” Cell 188, no. 2 (2025): 352–730. e22. 39814020 10.1016/j.cell.2024.12.005

[392] J. Shan , Z. Lin , H. Rashid , et al., “A Novel Therapeutic Strategy for Osteosarcoma Using Anti‐GD2 ADC and EZH2 Inhibitor,” Biomarker Research 13, no. 1 (2025): 87. 40533837 10.1186/s40364-025-00800-3 PMC12177979

[393] S. Yu and X. Yao , “Advances on Immunotherapy for Osteosarcoma,” Molecular Cancer 23, no. 1 (2024): 192. 39245737 10.1186/s12943-024-02105-9 PMC11382402

[394] B. Li , Z. Wang , H. Wu , et al., “Epigenetic Regulation of CXCL12 Plays a Critical Role in Mediating Tumor Progression and the Immune Response in Osteosarcoma,” Cancer Research 78, no. 14 (2018): 3938–3953. 29735547 10.1158/0008-5472.CAN-17-3801

[395] R. Ji , Y. Wang , D. Pan , et al., “NUCB2 inhibition Antagonizes Osteosarcoma Progression and Promotes Anti‐tumor Immunity Through Inactivating NUCKS1/CXCL8 Axis,” Cancer Letters 591 (2024): 216893. 38636892 10.1016/j.canlet.2024.216893

[396] K. Jiang , Q. Zhang , Y. Fan , et al., “MYC Inhibition Reprograms Tumor Immune Microenvironment by Recruiting T Lymphocytes and Activating the CD40/CD40L System in Osteosarcoma,” Cell Death Discovery 8, no. 1 (2022): 117. 35292660 10.1038/s41420-022-00923-8 PMC8924240

[397] K. Yahiro , Y. Matsumoto , H. Yamada , et al., “Activation of TLR4 Signaling Inhibits Progression of Osteosarcoma by Stimulating CD8‐positive Cytotoxic Lymphocytes,” Cancer Immunology, Immunotherapy 69, no. 5 (2020): 745–758. 32047957 10.1007/s00262-020-02508-9 PMC11027819

[398] L. Fernandez , J. Y. Metais , A. Escudero , et al., “Memory T Cells Expressing an NKG2D‐CAR Efficiently Target Osteosarcoma Cells,” Clinical Cancer Research 23, no. 19 (2017): 5824–5835. 28659311 10.1158/1078-0432.CCR-17-0075

[399] Y. Jin , Z. Jia , X. Xia , et al., “Anti‐CD137 Agonist Antibody‐independent and Clinically Feasible Preparation of Tumor‐infiltrating Lymphocytes From Soft Tissue Sarcoma and Osteosarcoma,” Frontiers in Immunology 16 (2025): 1557006. 40145091 10.3389/fimmu.2025.1557006 PMC11936977

[400] M. Liu , L. L. Sun , Y. J. Li , et al., “Trastuzumab Enhanced the Cytotoxicity of Vgamma9Vdelta2 T Cells Against Zoledronate‐sensitized Osteosarcoma Cells,” International Immunopharmacology 28, no. 1 (2015): 160–167. 26071219 10.1016/j.intimp.2015.06.002

[401] Y. Wang , X. Wang , Y. Liu , et al., “A Novel Hypoxia‐ and Lactate Metabolism‐related Prognostic Signature to Characterize the Immune Landscape and Predict Immunotherapy Response in Osteosarcoma,” Frontiers in Immunology 15 (2024): 1467052. 39569192 10.3389/fimmu.2024.1467052 PMC11576178

[402] Z. Zhang , W. Ji , J. Huang , Y. Zhang , Y. Zhou , and J. Zhang , “Characterization of the Tumour Microenvironment Phenotypes in Malignant Tissues and Pleural Effusion From Advanced Osteoblastic Osteosarcoma Patients,” Clinical and Translational Medicine 12, no. 11 (2022): e1072. 36305631 10.1002/ctm2.1072 PMC9615475

[403] Z. Wang , Z. Wang , B. Li , S. Wang , T. Chen , and Z. Ye , “Innate Immune Cells: A Potential and Promising Cell Population for Treating Osteosarcoma,” Frontiers in Immunology 10 (2019): 1114. 31156651 10.3389/fimmu.2019.01114 PMC6531991

[404] H. Gassmann , M. Thiede , J. Weiss , et al., “Cytokine Screening Identifies TNF to Potentially Enhance Immunogenicity of Pediatric Sarcomas,” Frontiers in Immunology 15 (2024): 1347404. 39723214 10.3389/fimmu.2024.1347404 PMC11668575

[405] Y. Ogiwara , M. Nakagawa , F. Nakatani , Y. Uemura , R. Zhang , and C. Kudo‐Saito , “Blocking FSTL1 Boosts NK Immunity in Treatment of Osteosarcoma,” Cancer Letters 537 (2022): 215690. 35439537 10.1016/j.canlet.2022.215690

[406] M. M. Cho , L. Song , A. E. Quamine , et al., “CD155 blockade Enhances Allogeneic Natural Killer Cell‐mediated Antitumor Response Against Osteosarcoma,” Journal for ImmunoTherapy of Cancer 13, no. 4 (2025): e008755. 40234092 10.1136/jitc-2023-008755 PMC12001373

[407] Y. Zhou , D. Yang , Q. Yang , et al., “Single‐cell RNA Landscape of Intratumoral Heterogeneity and Immunosuppressive Microenvironment in Advanced Osteosarcoma,” Nature Communications 11, no. 1 (2020): 6322. 10.1038/s41467-020-20059-6 PMC7730477 33303760

[408] W. Liu , H. Hu , Z. Shao , et al., “Characterizing the Tumor Microenvironment at the Single‐cell Level Reveals a Novel Immune Evasion Mechanism in Osteosarcoma,” Bone Research 11, no. 1 (2023): 4. 36596773 10.1038/s41413-022-00237-6 PMC9810605

[409] A. M. Taylor , J. Sheng , P. K. S. Ng , et al., “Immunosuppressive Tumor Microenvironment of Osteosarcoma,” Cancers (Basel) 17, no. 13 (2025): 2117. 40647416 10.3390/cancers17132117 PMC12248827

[410] A. J. Gentles , S. V. Bratman , L. J. Lee , et al., “Integrating Tumor and Stromal Gene Expression Signatures with Clinical Indices for Survival Stratification of Early‐Stage Non‐Small Cell Lung Cancer,” JNCI: Journal of the National Cancer Institute 107, no. 10 (2015): djv211. 26286589 10.1093/jnci/djv211 PMC6090873

[411] Q. Huang , X. Liang , T. Ren , et al., “The Role of Tumor‐associated Macrophages in Osteosarcoma Progression—therapeutic Implications,” Cellular Oncology (Dordrecht) 44, no. 3 (2021): 525–539. 10.1007/s13402-021-00598-w PMC12980758 33788151

[412] M. D. Thakur , C. J. Franz , L. Brennan , et al., “Immune Contexture of Paediatric Cancers,” European Journal of Cancer 170 (2022): 179–193. 35660252 10.1016/j.ejca.2022.03.012

[413] C. Dumars , J. M. Ngyuen , A. Gaultier , et al., “Dysregulation of Macrophage Polarization Is Associated With the Metastatic Process in Osteosarcoma,” Oncotarget 7, no. 48 (2016): 78343–78354. 27823976 10.18632/oncotarget.13055 PMC5346643

[414] W. Liu , Q. Long , W. Zhang , et al., “miRNA‐221‐3p Derived From M2‐polarized Tumor‐associated Macrophage Exosomes Aggravates the Growth and Metastasis of Osteosarcoma Through SOCS3/JAK2/STAT3 Axis,” Aging (Albany NY) 13, no. 15 (2021): 19760–19775. 34388111 10.18632/aging.203388 PMC8386545

[415] Y. Kimura and M. Sumiyoshi , “Anti‐tumor and Anti‐metastatic Actions of Wogonin Isolated From Scutellaria baicalensis Roots Through Anti‐lymphangiogenesis,” Phytomedicine 20, no. 3‐4 (2013): 328–336. 23219337 10.1016/j.phymed.2012.10.016

[416] J. A. Ligon , W. Choi , G. Cojocaru , et al., “Pathways of Immune Exclusion in Metastatic Osteosarcoma Are Associated With Inferior Patient Outcomes,” Journal for ImmunoTherapy of Cancer 9, no. 5 (2021): e001772. 34021032 10.1136/jitc-2020-001772 PMC8144029

[417] F. He , G. Ding , W. Jiang , X. Fan , and L. Zhu , “Effect of Tumor‐associated Macrophages on lncRNA PURPL/miR‐363/PDZD2 Axis in Osteosarcoma Cells,” Cell Death Discovery 7, no. 1 (2021): 307. 34686652 10.1038/s41420-021-00700-z PMC8536668

[418] J. Y. He , F. Y. Huo , H. C. Tang , B. Liu , and L. L. Bu , “Myeloid‐derived Suppressor Cells in Head and Neck Squamous Cell Carcinoma,” International Review of Cell and Molecular Biology 375 (2023): 33–92. 36967154 10.1016/bs.ircmb.2022.11.002

[419] E. Shokati and E. Safari , “The Immunomodulatory Role of Exosomal microRNA Networks in the Crosstalk Between Tumor‐associated Myeloid‐derived Suppressor Cells and Tumor Cells,” International Immunopharmacology 120 (2023): 110267. 37276829 10.1016/j.intimp.2023.110267

[420] S. Wang , X. Zhao , S. Wu , D. Cui , and Z. Xu , “Myeloid‐derived Suppressor Cells: Key Immunosuppressive Regulators and Therapeutic Targets in Hematological Malignancies,” Biomarker Research 11, no. 1 (2023): 34. 36978204 10.1186/s40364-023-00475-8 PMC10049909

[421] K. Jiang , J. Li , J. Zhang , et al., “SDF‐1/CXCR4 Axis Facilitates Myeloid‐derived Suppressor Cells Accumulation in Osteosarcoma Microenvironment and Blunts the Response to anti‐PD‐1 Therapy,” International Immunopharmacology 75 (2019): 105818. 31437795 10.1016/j.intimp.2019.105818

[422] Y. Guan , R. Zhang , Z. Peng , D. Dong , G. Wei , and Y. Wang , “Inhibition of IL‐18‐mediated Myeloid Derived Suppressor Cell Accumulation Enhances Anti‐PD1 Efficacy Against Osteosarcoma Cancer,” Journal of Bone Oncology 9 (2017): 59–64. 29226090 10.1016/j.jbo.2017.10.002 PMC5715437

[423] X. Shi , X. Li , H. Wang , Z. Yu , Y. Zhu , and Y. Gao , “Specific Inhibition of PI3Kdelta/Gamma Enhances the Efficacy of Anti‐PD1 Against Osteosarcoma Cancer,” Journal of Bone Oncology 16 (2019): 100206. 31334002 10.1016/j.jbo.2018.11.001 PMC6617297

[424] D. T. Ammons , R. A. Harris , L. S. Hopkins , J. Kurihara , K. Weishaar , and S. Dow , “A Single‐cell RNA Sequencing Atlas of Circulating Leukocytes From Healthy and Osteosarcoma Affected Dogs,” Frontiers in Immunology 14 (2023): 1162700. 37275879 10.3389/fimmu.2023.1162700 PMC10235626

[425] C. Deng , Y. Xu , J. Fu , et al., “Reprograming the Tumor Immunologic Microenvironment Using Neoadjuvant Chemotherapy in Osteosarcoma,” Cancer Science 111, no. 6 (2020): 1899–1909. 32232912 10.1111/cas.14398 PMC7293104

[426] M. Kansara , K. Thomson , P. Pang , et al., “Infiltrating Myeloid Cells Drive Osteosarcoma Progression via GRM4 Regulation of IL23,” Cancer Discovery 9, no. 11 (2019): 1511–1519. 31527131 10.1158/2159-8290.CD-19-0154

[427] B. J. Biller , A. Guth , J. H. Burton , and S. W. Dow , “Decreased Ratio of CD8+ T Cells to Regulatory T Cells Associated With Decreased Survival in Dogs With Osteosarcoma,” Journal of Veterinary Internal Medicine 24, no. 5 (2010): 1118–1123. 20666983 10.1111/j.1939-1676.2010.0557.x PMC3557512

[428] B. Fritzsching , J. Fellenberg , L. Moskovszky , et al., “CD8(+)/FOXP3(+)‐ratio in Osteosarcoma Microenvironment Separates Survivors From Non‐survivors: A Multicenter Validated Retrospective Study,” Oncoimmunology 4, no. 3 (2015): e990800. 25949908 10.4161/2162402X.2014.990800 PMC4404826

[429] M. Yang , H. Zheng , K. Xu , et al., “A Novel Signature to Guide Osteosarcoma Prognosis and Immune Microenvironment: Cuproptosis‐related lncRNA,” Frontiers in Immunology 13 (2022): 919231. 35967366 10.3389/fimmu.2022.919231 PMC9373797

[430] M. X. Liu , Q. Y. Liu , Y. Liu , et al., “Interleukin‐35 Suppresses Antitumor Activity of Circulating CD8(+) T Cells in Osteosarcoma Patients,” Connective Tissue Research 60, no. 4 (2019): 367–375. 30616389 10.1080/03008207.2018.1552267

[431] X. Li , Y. Chen , X. Liu , et al., “Tim3/Gal9 interactions Between T Cells and Monocytes Result in an Immunosuppressive Feedback Loop That Inhibits Th1 Responses in Osteosarcoma Patients,” International Immunopharmacology 44 (2017): 153–159. 28103502 10.1016/j.intimp.2017.01.006

[432] Y. Takahashi , T. Yasui , K. Tamari , et al., “Radiation Enhanced the Local and Distant Anti‐tumor Efficacy in Dual Immune Checkpoint Blockade Therapy in Osteosarcoma,” PLoS ONE 12, no. 12 (2017): e0189697. 29253865 10.1371/journal.pone.0189697 PMC5734786

[433] K. Yoshida , M. Okamoto , J. Sasaki , et al., “Anti‐PD‐1 Antibody Decreases Tumour‐infiltrating Regulatory T Cells,” BMC Cancer 20, no. 1 (2020): 25. 31914969 10.1186/s12885-019-6499-y PMC6950856

[434] E. Kozawa , H. Sugiura , J. Wasa , et al., “Suppression of Tumour Metastasis in a Murine Osteosarcoma Model With Anti‐CD25 Monoclonal Antibody Treatment,” Anticancer Research 30, no. 12 (2010): 5019–5022. 21187484

[435] J. Zhang , Y. Li , S. Yang , L. Zhang , and W. Wang , “Anti‐CD40 mAb Enhanced Efficacy of Anti‐PD1 Against Osteosarcoma,” Journal of Bone Oncology 17 (2019): 100245. 31293882 10.1016/j.jbo.2019.100245 PMC6593232

[436] D. Lavie , A. Ben‐Shmuel , N. Erez , and R. Scherz‐Shouval , “Cancer‐associated Fibroblasts in the Single‐cell Era,” Nature Cancer 3, no. 7 (2022): 793–807. 35883004 10.1038/s43018-022-00411-z PMC7613625

[437] G. Biffi and D. A. Tuveson , “Diversity and Biology of Cancer‐Associated Fibroblasts,” Physiological Reviews 101, no. 1 (2021): 147–176. 32466724 10.1152/physrev.00048.2019 PMC7864232

[438] X. Mao , J. Xu , W. Wang , et al., “Crosstalk Between Cancer‐associated Fibroblasts and Immune Cells in the Tumor Microenvironment: New Findings and Future Perspectives,” Molecular Cancer 20, no. 1 (2021): 131. 34635121 10.1186/s12943-021-01428-1 PMC8504100

[439] A. Obradovic , D. Graves , M. Korrer , et al., “Immunostimulatory Cancer‐Associated Fibroblast Subpopulations Can Predict Immunotherapy Response in Head and Neck Cancer,” Clinical Cancer Research 28, no. 10 (2022): 2094–2109. 35262677 10.1158/1078-0432.CCR-21-3570 PMC9161438

[440] B. C. Ozdemir , T. Pentcheva‐Hoang , J. L. Carstens , et al., “Depletion of Carcinoma‐associated Fibroblasts and Fibrosis Induces Immunosuppression and Accelerates Pancreas Cancer With Reduced Survival,” Cancer Cell 25, no. 6 (2014): 719–734. 24856586 10.1016/j.ccr.2014.04.005 PMC4180632

[441] Y. Zhang , Z. Liu , X. Yang , et al., “H3K27 acetylation Activated‐COL6A1 Promotes Osteosarcoma Lung Metastasis by Repressing STAT1 and Activating Pulmonary Cancer‐associated Fibroblasts,” Theranostics 11, no. 3 (2021): 1473–1492. 33391546 10.7150/thno.51245 PMC7738898

[442] A. Mazumdar , J. Urdinez , A. Boro , et al., “Osteosarcoma‐Derived Extracellular Vesicles Induce Lung Fibroblast Reprogramming,” International Journal of Molecular Sciences 21, no. 15 (2020): 5451. 32751693 10.3390/ijms21155451 PMC7432951

[443] L. Lin , K. Huang , W. Guo , C. Zhou , G. Wang , and Q. Zhao , “Conditioned Medium of the Osteosarcoma Cell Line U2OS Induces hBMSCs to Exhibit Characteristics of Carcinoma‐associated Fibroblasts via Activation of IL‐6/STAT3 Signalling,” Journal of Biochemistry 168, no. 3 (2020): 265–271. 32302384 10.1093/jb/mvaa044

[444] X. Chang , Q. Tan , J. Xu , et al., “Tumor‐derived Exosomal linc00881 Induces Lung Fibroblast Activation and Promotes Osteosarcoma Lung Migration,” Cancer Cell International 23, no. 1 (2023): 287. 37990331 10.1186/s12935-023-03121-3 PMC10664679

[445] Y. Xu , P. Chen , D. Liu , Q. Xu , H. Meng , and X. Wang , “Exploration of s New Biomarker in Osteosarcoma and Association With Clinical Outcomes: (TOP2A+) Cancer Associated Fibroblasts,” The Journal of Gene Medicine 25, no. 11 (2023): e3528. 37246449 10.1002/jgm.3528

[446] S. Jaillon , A. Ponzetta , D. Di Mitri , A. Santoni , R. Bonecchi , and A. Mantovani , “Neutrophil Diversity and Plasticity in Tumour Progression and Therapy,” Nature Reviews Cancer 20, no. 9 (2020): 485–503. 32694624 10.1038/s41568-020-0281-y

[447] A. Mantovani , “The Yin‐yang of Tumor‐associated Neutrophils,” Cancer Cell 16, no. 3 (2009): 173–174. 19732714 10.1016/j.ccr.2009.08.014

[448] H. Que , Q. Fu , T. Lan , X. Tian , and X. Wei , “Tumor‐associated Neutrophils and Neutrophil‐targeted Cancer Therapies,” Biochimica et Biophysica Acta (BBA) ‐ Reviews on Cancer 1877, no. 5 (2022): 188762. 35853517 10.1016/j.bbcan.2022.188762

[449] R. Sun , J. Luo , D. Li , et al., “Neutrophils With Protumor Potential Could Efficiently Suppress Tumor Growth After Cytokine Priming and in Presence of Normal NK Cells,” Oncotarget 5, no. 24 (2014): 12621–12634. 25587026 10.18632/oncotarget.2181 PMC4350330

[450] S. Tan and R. Chao , “An Exploration of Osteosarcoma Metastasis Diagnostic Markers Based on Tumor‐Associated Neutrophils,” Discovery Medicine 35, no. 176 (2023): 300–311. 37272097 10.24976/Discov.Med.202335176.31

[451] W. D. Tap , G. M. Cote , H. Burris , et al., “Phase I Study of the Mutant IDH1 Inhibitor Ivosidenib: Long‐term Safety and Clinical Activity in Patients With Conventional Chondrosarcoma,” Clinical Cancer Research 31, no. 11 (2025): 2108–2114. 40100120 10.1158/1078-0432.CCR-24-4128 PMC12130799

[452] B. Li , G. Li , X. Yan , et al., “Fresh Tissue Multi‐omics Profiling Reveals Immune Classification and Suggests Immunotherapy Candidates for Conventional Chondrosarcoma,” Clinical Cancer Research 27, no. 23 (2021): 6543–6558. 34426437 10.1158/1078-0432.CCR-21-1893 PMC9401490

[453] F. A. Simard , I. Richert , A. Vandermoeten , et al., “Description of the Immune Microenvironment of Chondrosarcoma and Contribution to Progression,” Oncoimmunology 6, no. 2 (2017): e1265716. 28344871 10.1080/2162402X.2016.1265716 PMC5353901

[454] M. Minopoli , S. Sarno , G. Di Carluccio , et al., “Inhibiting Monocyte Recruitment to Prevent the Pro‐Tumoral Activity of Tumor‐Associated Macrophages in Chondrosarcoma,” Cells 9, no. 4 (2020): 1062. 32344648 10.3390/cells9041062 PMC7226304

[455] R. Iseulys , G. B. Anne , B. Corinne , D. B. P. Gonzague , K. Marie , and B. Jean‐Yves , “The Immune Landscape of Chondrosarcoma Reveals an Immunosuppressive Environment in the Dedifferentiated Subtypes and Exposes CSFR1+ Macrophages as a Promising Therapeutic Target,” Journal of Bone Oncology 20 (2020): 100271. 31956474 10.1016/j.jbo.2019.100271 PMC6961717

[456] M. Kostine , A. H. Cleven , N. F. de Miranda , A. Italiano , A. M. Cleton‐Jansen , and J. V. Bovee , “Analysis of PD‐L1, T‐cell Infiltrate and HLA Expression in Chondrosarcoma Indicates Potential for Response to Immunotherapy Specifically in the Dedifferentiated Subtype,” Modern Pathology 29, no. 9 (2016): 1028–1037. 27312065 10.1038/modpathol.2016.108

[457] C. Li , W. Wang , B. Zhong , et al., “Long Noncoding RNA TUG1 Promotes Chondrosarcoma Progression and M2 Polarization,” Genes & Diseases 12, no. 4 (2025): 101474. 40330150 10.1016/j.gendis.2024.101474 PMC12052688

[458] R. Quoniou , E. Moreau , F. Cachin , et al., “Chondrosarcoma Co‐Culture 3D Model Horizontal Line an Insight to Evaluate Drugs Acting on TAMs,” ACS Biomaterials Science & Engineering 10, no. 9 (2024): 5832–5843. 39121344 10.1021/acsbiomaterials.4c00625

[459] Y. Wen , Y. Li , S. Cheng , et al., “Partition‐Less Digital Immunoassay Using Configurable Topographic Nanoarrays for Extracellular Vesicle Diagnosis of Ewing Sarcoma,” ACS Nano 19, no. 12 (2025): 11973–11986.40115997 10.1021/acsnano.4c16904

[460] E. D. Wrenn , A. A. Apfelbaum , E. R. Rudzinski , et al., “Cancer‐Associated Fibroblast‐Like Tumor Cells Remodel the Ewing Sarcoma Tumor Microenvironment,” Clinical Cancer Research 29, no. 24 (2023): 5140–5154. 37471463 10.1158/1078-0432.CCR-23-1111 PMC10801911

[461] E. Morales , M. Olson , F. Iglesias , S. Dahiya , T. Luetkens , and D. Atanackovic , “Role of Immunotherapy in Ewing Sarcoma,” Journal for ImmunoTherapy of Cancer 8, no. 2 (2020): e000653. 33293354 10.1136/jitc-2020-000653 PMC7725096

[462] D. Berghuis , A. S. de Hooge , S. J. Santos , et al., “Reduced human Leukocyte Antigen Expression in Advanced‐stage Ewing Sarcoma: Implications for Immune Recognition,” Journal of Pathology 218, no. 2 (2009): 222–231. 19274709 10.1002/path.2537

[463] I. C. Henrich , K. Jain , R. Young , et al., “Ubiquitin‐Specific Protease 6 Functions as a Tumor Suppressor in Ewing Sarcoma Through Immune Activation,” Cancer Research 81, no. 8 (2021): 2171–2183. 33558334 10.1158/0008-5472.CAN-20-1458 PMC8137534

[464] W. Luo , H. Hoang , H. Zhu , et al., “Circumventing Resistance Within the Ewing Sarcoma Microenvironment by Combinatorial Innate Immunotherapy,” Journal for ImmunoTherapy of Cancer 12, no. 9 (2024): e009726. 39266215 10.1136/jitc-2024-009726 PMC11404285

[465] H. Zhang , I. Maric , M. J. DiPrima , et al., “Fibrocytes Represent a Novel MDSC Subset Circulating in Patients With Metastatic Cancer,” Blood 122, no. 7 (2013): 1105–1113. 23757729 10.1182/blood-2012-08-449413 PMC3744987

[466] N. L. Denton , C. Y. Chen , B. Hutzen , et al., “Myelolytic Treatments Enhance Oncolytic Herpes Virotherapy in Models of Ewing Sarcoma by Modulating the Immune Microenvironment,” Molecular Therapy Oncolytics 11 (2018): 62–74. 30505937 10.1016/j.omto.2018.10.001 PMC6249791

[467] P. Brinkrolf , S. Landmeier , B. Altvater , et al., “A High Proportion of Bone Marrow T Cells With Regulatory Phenotype (CD4+CD25hiFoxP3+) in Ewing Sarcoma Patients Is Associated With Metastatic Disease,” International Journal of Cancer 125, no. 4 (2009): 879–886. 19480009 10.1002/ijc.24461

[468] T. V. Tilak , S. Sharawat , R. Gupta , S. Agarwala , S. Vishnubhatla , and S. Bakhshi , “Circulating T‐regulatory Cells in PNET: A Prospective Study,” Pediatric Blood & Cancer 61, no. 2 (2014): 228–232. 23997029 10.1002/pbc.24734

[469] S. J. Schober , C. Schoening , J. Eck , et al., “The Oncolytic Adenovirus XVir‐N‐31 Joins Forces With CDK4/6 Inhibition Augmenting Innate and Adaptive Antitumor Immunity in Ewing Sarcoma,” Clinical Cancer Research 29, no. 10 (2023): 1996–2011. 36892582 10.1158/1078-0432.CCR-22-1961

[470] P. Sargos , M. P. Sunyach , A. Ducassou , et al., “Results of a Phase Ib Study of olaparib With Concomitant Radiotherapy in Soft‐tissue Sarcoma: A French Sarcoma Group Study,” Annals of Oncology 36, no. 5 (2025): 592–600. 39894354 10.1016/j.annonc.2025.01.016

[471] E. Jumaniyazova , A. Lokhonina , D. Dzhalilova , A. Kosyreva , and T. Fatkhudinov , “Immune Cells in the Tumor Microenvironment of Soft Tissue Sarcomas,” Cancers (Basel) 15, no. 24 (2023): 5760. 38136307 10.3390/cancers15245760 PMC10741982

[472] D. S. Moura , J. M. Lopez‐Marti , I. Benesova , et al., “Predictive and Dynamic Signature for Antiangiogenics in Combination With a PD1 Inhibitor in Soft‐Tissue Sarcoma: Correlative Studies Linked to the IMMUNOSARC Trial,” Clinical Cancer Research 30, no. 22 (2024): 5192–5206. 39283727 10.1158/1078-0432.CCR-24-1782

[473] B. A. Schroeder , N. A. LaFranzo , B. J. LaFleur , et al., “CD4+ T Cell and M2 Macrophage Infiltration Predict Dedifferentiated Liposarcoma Patient Outcomes,” Journal for ImmunoTherapy of Cancer 9, no. 8 (2021): e002812. 34465597 10.1136/jitc-2021-002812 PMC8413967

[474] N. Matsuda , H. Yamamoto , T. Habu , et al., “Prognostic Impact of Tumor‐Infiltrating Lymphocytes, Tertiary Lymphoid Structures, and Neutrophil‐to‐Lymphocyte Ratio in Pulmonary Metastases From Uterine Leiomyosarcoma,” Annals of Surgical Oncology 30, no. 13 (2023): 8727–8734. 37658268 10.1245/s10434-023-14176-x PMC10625945

[475] Y. Klaver , M. Rijnders , A. Oostvogels , et al., “Differential Quantities of Immune Checkpoint‐expressing CD8 T Cells in Soft Tissue Sarcoma Subtypes,” Journal for ImmunoTherapy of Cancer 8, no. 2 (2020): e000271. 32792357 10.1136/jitc-2019-000271 PMC7430493

[476] S. van Oost , D. M. Meijer , Z. B. Erdem , et al., “Divergent Therapeutic and Prognostic Impacts of Immunogenic Features in Undifferentiated Pleomorphic Sarcoma and Myxofibrosarcoma,” Cancer Immunology, Immunotherapy 74, no. 8 (2025): 258. 40601026 10.1007/s00262-025-04123-y PMC12222583

[477] K. M. Campbell , M. Thaker , E. Medina , et al., “Spatial Profiling Reveals Association Between WNT Pathway Activation and T‐cell Exclusion in Acquired Resistance of Synovial Sarcoma to NY‐ESO‐1 Transgenic T‐cell Therapy,” Journal for ImmunoTherapy of Cancer 10, no. 3 (2022): e004190. 35264439 10.1136/jitc-2021-004190 PMC8915285

[478] A. M. Fuller , H. C. Pruitt , Y. Liu , et al., “Oncogene‐induced Matrix Reorganization Controls CD8+ T Cell Function in the Soft‐tissue Sarcoma Microenvironment,” Journal of Clinical Investigation 134, no. 11 (2024): e167826. 38652549 10.1172/JCI167826 PMC11142734

[479] M. Kanahori , E. Shimada , Y. Matsumoto , et al., “Immune Evasion in Lung Metastasis of Leiomyosarcoma: Upregulation of EPCAM Inhibits CD8(+) T Cell Infiltration,” British Journal of Cancer 130, no. 7 (2024): 1083–1095. 38291183 10.1038/s41416-024-02576-z PMC10991329

[480] W. De Wispelaere , D. Annibali , S. Tuyaerts , et al., “PI3K/mTOR Inhibition Induces Tumour Microenvironment Remodelling and Sensitises pS6(high) Uterine Leiomyosarcoma to PD‐1 Blockade,” Clinical and Translational Medicine 14, no. 5 (2024): e1655. 38711203 10.1002/ctm2.1655 PMC11074386

[481] J. Yang , X. Lu , Q. Cai , et al., “Loss of TACC2 Impairs Chemokine CCL3 and CCL4 Expression and Reduces Response to anti‐PD‐1 Therapy in Soft Tissue Sarcoma,” Molecular Cancer 24, no. 1 (2025): 158. 40442694 10.1186/s12943-025-02354-2 PMC12123857

[482] H. G. Smith , K. Jamal , J. H. Dayal , et al., “RIPK1‐mediated Immunogenic Cell Death Promotes Anti‐tumour Immunity Against Soft‐tissue Sarcoma,” EMBO Molecular Medicine 12, no. 6 (2020): e10979. 32419365 10.15252/emmm.201910979 PMC7278545

[483] L. Rupp , A. Resag , V. Potkrajcic , et al., “Prognostic Impact of the Post‐treatment T Cell Composition and Spatial Organization in Soft Tissue Sarcoma Patients Treated With Neoadjuvant Hyperthermic Radio(chemo)Therapy,” Frontiers in Immunology 14 (2023): 1185197. 37261361 10.3389/fimmu.2023.1185197 PMC10228739

[484] J. S. Almeida , L. M. Sousa , P. Couceiro , et al., “Peripheral Immune Profiling of Soft Tissue Sarcoma: Perspectives for Disease Monitoring,” Frontiers in Immunology 15 (2024): 1391840. 39502689 10.3389/fimmu.2024.1391840 PMC11536262

[485] L. M. Sousa , J. S. Almeida , T. Fortes‐Andrade , et al., “Comprehensive Receptor Repertoire and Functional Analysis of Peripheral NK Cells in Soft Tissue Sarcoma Patients,” Cancers (Basel) 17, no. 15 (2025): 2508. 40805205 10.3390/cancers17152508 PMC12346150

[486] S. Y. Neo , L. Tong , J. Chong , et al., “Tumor‐associated NK Cells Drive MDSC‐mediated Tumor Immune Tolerance Through the IL‐6/STAT3 Axis,” Science Translational Medicine 16, no. 747 (2024): eadi2952. 38748775 10.1126/scitranslmed.adi2952

[487] S. M. Cruz , C. J. Sholevar , S. J. Judge , et al., “Intratumoral NKp46(+) Natural Killer Cells Are Spatially Distanced From T and MHC‐I(+) Cells With Prognostic Implications in Soft Tissue Sarcoma,” Frontiers in Immunology 14 (2023): 1230534. 37545516 10.3389/fimmu.2023.1230534 PMC10401426

[488] P. Taborska , P. Lukac , D. Stakheev , et al., “Novel PD‐L1‐ and Collagen‐expressing Patient‐derived Cell Line of Undifferentiated Pleomorphic Sarcoma (JBT19) as a Model for Cancer Immunotherapy,” Scientific Reports 13, no. 1 (2023): 19079. 37925511 10.1038/s41598-023-46305-7 PMC10625569

[489] Y. Murayama , Y. Kasahara , N. Kubo , et al., “NKp44‐based Chimeric Antigen Receptor Effectively Redirects Primary T Cells Against Synovial Sarcoma,” Translational Oncology 25 (2022): 101521. 35998437 10.1016/j.tranon.2022.101521 PMC9420389

[490] B. Choi , J. S. Lee , S. J. Kim , D. Hong , J. B. Park , and K. Y. Lee , “Anti‐tumor Effects of Anti‐PD‐1 Antibody, Pembrolizumab, in Humanized NSG PDX Mice Xenografted With Dedifferentiated Liposarcoma,” Cancer Letters 478 (2020): 56–69. 32145342 10.1016/j.canlet.2020.02.042

[491] X. Lu , M. Liu , J. Yang , Y. Que , and X. Zhang , “Panobinostat Enhances NK Cell Cytotoxicity in Soft Tissue Sarcoma,” Clinical and Experimental Immunology 209, no. 2 (2022): 127–139. 35867577 10.1093/cei/uxac068 PMC9390845

[492] L. Yan , Z. Wang , C. Cui , et al., “Comprehensive Immune Characterization and T‐cell Receptor Repertoire Heterogeneity of Retroperitoneal Liposarcoma,” Cancer Science 110, no. 10 (2019): 3038–3048. 31385405 10.1111/cas.14161 PMC6778648

[493] G. Avallone , E. Brigandi , C. Tugnoli , A. Rigillo , B. Bacci , and P. Roccabianca , “Tumor‐infiltrating Lymphocytes Vary in Different Canine Soft Tissue Sarcoma Histological Types,” Veterinary Pathology 62, no. 3 (2025): 276–283. 39651618 10.1177/03009858241300556

[494] M. A. Smolle , L. Herbsthofer , M. Goda , et al., “Influence of Tumor‐infiltrating Immune Cells on Local Control Rate, Distant Metastasis, and Survival in Patients With Soft Tissue Sarcoma,” Oncoimmunology 10, no. 1 (2021): 1896658. 33763294 10.1080/2162402X.2021.1896658 PMC7954425

[495] P. Tsagozis , M. Augsten , Y. Zhang , et al., “An Immunosuppressive Macrophage Profile Attenuates the Prognostic Impact of CD20‐positive B Cells in human Soft Tissue Sarcoma,” Cancer Immunology, Immunotherapy 68, no. 6 (2019): 927–936. 30879106 10.1007/s00262-019-02322-y PMC6529392

[496] J. Y. Hong , H. J. Cho , K. H. Yun , et al., “Comprehensive Molecular Characterization of Soft Tissue Sarcoma for Prediction of Pazopanib‐Based Treatment Response,” Cancer Research and Treatment: Official Journal of Korean Cancer Association 55, no. 2 (2023): 671–683. 10.4143/crt.2022.251 PMC10101793 36164943

[497] C. L. Roland , E. F. Nassif Haddad , E. Z. Keung , W. L. Wang , A. J. Lazar , and H. Lin , “A Randomized, Non‐comparative Phase 2 Study of Neoadjuvant Immune‐checkpoint Blockade in Retroperitoneal Dedifferentiated Liposarcoma and Extremity/Truncal Undifferentiated Pleomorphic Sarcoma,” Nature Cancer 5, no. 4 (2024): 625–641. 38351182 10.1038/s43018-024-00726-z PMC12955605

[498] A. Salawu , B. X. Wang , M. Han , et al., “Safety, Immunologic, and Clinical Activity of Durvalumab in Combination With Olaparib or Cediranib in Advanced Leiomyosarcoma: Results of the DAPPER Clinical Trial,” Clinical Cancer Research 29, no. 20 (2023): 4128–4138. 37566240 10.1158/1078-0432.CCR-23-1137

[499] Y. Que , W. Xiao , Y. X. Guan , Y. Liang , S. M. Yan , and H. Y. Chen , “PD‐L1 Expression Is Associated With FOXP3+ Regulatory T‐Cell Infiltration of Soft Tissue Sarcoma and Poor Patient Prognosis,” Journal of Cancer 8, no. 11 (2017): 2018–2025. 28819402 10.7150/jca.18683 PMC5559963

[500] M. A. Smolle , L. Herbsthofer , B. Granegger , et al., “T‐regulatory Cells Predict Clinical Outcome in Soft Tissue Sarcoma Patients: A Clinico‐pathological Study,” British Journal of Cancer 125, no. 5 (2021): 717–724. 34127811 10.1038/s41416-021-01456-0 PMC8405702

[501] H. Chen , Y. Chen , H. Liu , Y. Que , X. Zhang , and F. Zheng , “Integrated Expression Profiles Analysis Reveals Correlations between the IL‐33/ST2 Axis and CD8(+) T Cells, Regulatory T Cells, and Myeloid‐Derived Suppressor Cells in Soft Tissue Sarcoma,” Frontiers in Immunology 9 (2018): 1179. 29896199 10.3389/fimmu.2018.01179 PMC5986931

[502] M. Spalato‐Ceruso , F. Bouteiller , J. P. Guegan , et al., “Pembrolizumab Combined With Low‐dose Cyclophosphamide and Intra‐tumoral Injection of the Toll‐Like Receptor 4 Agonist G100 in Patients With Advanced Pretreated Soft Tissue Sarcoma: Results From the PEMBROSARC Basket Study,” Journal of Hematology & Oncology 15, no. 1 (2022): 157. 36303228 10.1186/s13045-022-01377-2 PMC9609223

[503] Y. Kim , E. Kobayashi , Y. Suehara , et al., “Immunological Status of Peripheral Blood Is Associated With Prognosis in Patients With Bone and Soft‐tissue Sarcoma,” Oncology Letters 21, no. 3 (2021): 212. 33510813 10.3892/ol.2021.12473 PMC7836390

[504] S. E. Finkelstein , C. Iclozan , M. M. Bui , et al., “Combination of External Beam Radiotherapy (EBRT) With Intratumoral Injection of Dendritic Cells as Neo‐adjuvant Treatment of High‐risk Soft Tissue Sarcoma Patients,” International Journal of Radiation and Oncology in Biology and Physics 82, no. 2 (2012): 924–932. 10.1016/j.ijrobp.2010.12.068 PMC4241354 21398051

[505] A. Levy , D. Morel , M. Texier , et al., “Monocyte‐lineage Tumor Infiltration Predicts Immunoradiotherapy Response in Advanced Pretreated Soft‐tissue Sarcoma: Phase 2 Trial Results,” Signal Transduction and Targeted Therapy 10, no. 1 (2025): 103. 40097400 10.1038/s41392-025-02173-3 PMC11914280

[506] N. Oike , H. Kawashima , A. Ogose , et al., “Prognostic Impact of the Tumor Immune Microenvironment in Synovial Sarcoma,” Cancer Science 109, no. 10 (2018): 3043–3054. 30133055 10.1111/cas.13769 PMC6172059

[507] M. Minopoli , S. Sarno , L. Cannella , et al., “Crosstalk Between Macrophages and Myxoid Liposarcoma Cells Increases Spreading and Invasiveness of Tumor Cells,” Cancers (Basel) 13, no. 13 (2021): 3298. 34209309 10.3390/cancers13133298 PMC8268435

[508] K. R. Patel , A. Martinez , J. M. Stahl , et al., “Increase in PD‐L1 Expression After Pre‐operative Radiotherapy for Soft Tissue Sarcoma,” Oncoimmunology 7, no. 7 (2018): e1442168. 29900051 10.1080/2162402X.2018.1442168 PMC5993497

[509] K. M. Skubitz , J. D. Wilson , E. Y. Cheng , B. R. Lindgren , K. L. M. Boylan , and A. P. N. Skubitz , “Effect of Chemotherapy on Cancer Stem Cells and Tumor‐associated Macrophages in a Prospective Study of Preoperative Chemotherapy in Soft Tissue Sarcoma,” Journal of Translational Medicine 17, no. 1 (2019): 130. 30999901 10.1186/s12967-019-1883-6 PMC6471853

[510] F. H. G. Tessaro , E. Y. Ko , M. De Simone , et al., “Single‐cell RNA‐seq of a Soft‐tissue Sarcoma Model Reveals the Critical Role of Tumor‐expressed MIF in Shaping Macrophage Heterogeneity,” Cell Reports 39, no. 12 (2022): 110977. 35732118 10.1016/j.celrep.2022.110977 PMC9249098

[511] J. J. Barrott , L. A. Kafchinski , H. Jin , et al., “Modeling Synovial Sarcoma Metastasis in the Mouse: PI3'‐lipid Signaling and Inflammation,” Journal of Experimental Medicine 213, no. 13 (2016): 2989–3005. 27956588 10.1084/jem.20160817 PMC5154942

[512] L. Casadei , F. Calore , C. J. Creighton , et al., “Exosome‐Derived miR‐25‐3p and miR‐92a‐3p Stimulate Liposarcoma Progression,” Cancer Research 77, no. 14 (2017): 3846–3856. 28588009 10.1158/0008-5472.CAN-16-2984 PMC6033276

[513] I. Benesova , L. Capkova , A. Ozaniak , et al., “A Comprehensive Analysis of CD47 Expression in Various Histological Subtypes of Soft Tissue Sarcoma: Exploring Novel Opportunities for Macrophage‐directed Treatments,” Journal of Cancer Research and Clinical Oncology 150, no. 3 (2024): 134. 38493445 10.1007/s00432-024-05661-1 PMC10944806

[514] S. Ishihara , T. Iwasaki , K. Kohashi , et al., “Clinical Significance of Signal Regulatory Protein Alpha and T Cell Immunoreceptor With Immunoglobulin and Immunoreceptor Tyrosine‐based Inhibition Motif Domain Expression in Undifferentiated Pleomorphic Sarcoma,” Journal of Cancer Research and Clinical Oncology 149, no. 6 (2023): 2425–2436. 35737088 10.1007/s00432-022-04078-y PMC11797676

[515] M. Umakoshi , Y. Kudo‐Asabe , H. Tsuchie , et al., “Prognostic Value of Cancer‐Associated Fibroblast Marker Expression in the Intratumoral and Marginal Areas of Soft Tissue Sarcoma,” Pathobiology 92, no. 1 (2025): 1–17. 38964294 10.1159/000539855 PMC11797935

[516] C. Xu , L. Yan , X. Guan , et al., “Tsp2 Facilitates Tumor‐associated Fibroblasts Formation and Promotes Tumor Progression in Retroperitoneal Liposarcoma,” International Journal of Biological Sciences 18, no. 13 (2022): 5038–5055. 35982904 10.7150/ijbs.70083 PMC9379409

[517] C. Bai , S. Li , Z. Tan , and Z. Fan , “Targeting MCM2 Activates Cancer‐associated Fibroblasts‐Like Phenotype and Affects Chemo‐resistance of Liposarcoma Cells Against Doxorubicin,” Anti‐Cancer Drugs 35, no. 10 (2024): 883–892. 39109389 10.1097/CAD.0000000000001641

[518] Y. Nagao , A. Yokoi , K. Yoshida , et al., “Uterine Leiomyosarcoma Cell‐derived Extracellular Vesicles Induce the Formation of Cancer‐associated Fibroblasts,” Biochimica et Biophysica Acta: Molecular Basis of Disease 1870, no. 4 (2024): 167103. 38417460 10.1016/j.bbadis.2024.167103

[519] M. Panagi , F. Mpekris , C. Voutouri , et al., “Stabilizing Tumor‐Resident Mast Cells Restores T‐Cell Infiltration and Sensitizes Sarcomas to PD‐L1 Inhibition,” Clinical Cancer Research 30, no. 11 (2024): 2582–2597. 38578281 10.1158/1078-0432.CCR-24-0246 PMC11145177

[520] M. T. Broz , E. Y. Ko , K. Ishaya , et al., “Metabolic Targeting of Cancer Associated Fibroblasts Overcomes T‐cell Exclusion and Chemoresistance in Soft‐tissue Sarcomas,” Nature Communications 15, no. 1 (2024): 2498. 10.1038/s41467-024-46504-4 PMC10954767 38509063

[521] B. W. Zheng , M. L. Yang , W. Huang , et al., “Prognostic Significance of Tumor‐Associated Macrophages in Chondroblastoma and Their Association With Response to Adjuvant Radiotherapy,” Journal of Inflammation Research 14 (2021): 1991–2005. 34040412 10.2147/JIR.S308707 PMC8139723

[522] S. Bissinger , C. Hage , V. Wagner , et al., “Macrophage Depletion Induces Edema Through Release of Matrix‐degrading Proteases and Proteoglycan Deposition,” Science Translational Medicine 13, no. 598 (2021): eabd4550. 34135110 10.1126/scitranslmed.abd4550

[523] J. R. Kalden and H. Schulze‐Koops , “Immunogenicity and Loss of Response to TNF Inhibitors: Implications for Rheumatoid Arthritis Treatment,” Nature Reviews Rheumatology 13, no. 12 (2017): 707–718. 29158574 10.1038/nrrheum.2017.187

[524] A. Rubbert‐Roth , F. Atzeni , I. F. Masala , R. Caporali , C. Montecucco , and P. Sarzi‐Puttini , “TNF Inhibitors in Rheumatoid Arthritis and Spondyloarthritis: Are They the Same?,” Autoimmunity Reviews 17, no. 1 (2018): 24–28. 29108829 10.1016/j.autrev.2017.11.005

[525] Y. Tanaka , Y. Miyazaki , M. Kawanishi , H. Yamasaki , and T. Takeuchi , “Long‐term Safety and Efficacy of Anti‐TNF Multivalent VHH Antibodies ozoralizumab in Patients With Rheumatoid Arthritis,” RMD Open 10, no. 3 (2024): e004480. 39179257 10.1136/rmdopen-2024-004480 PMC11344530

[526] J. S. Smolen , E. Feist , S. Fatenejad , et al., “Olokizumab versus Placebo or Adalimumab in Rheumatoid Arthritis,” New England Journal of Medicine 387, no. 8 (2022): 715–726. 36001712 10.1056/NEJMoa2201302

[527] R. Fleischmann , M. C. Genovese , K. Maslova , H. Leher , A. Praestgaard , and G. R. Burmester , “Long‐term Safety and Efficacy of sarilumab Over 5 Years in Patients With Rheumatoid Arthritis Refractory to TNF Inhibitors,” Rheumatology 60, no. 11 (2021): 4991–5001. 33871596 10.1093/rheumatology/keab355

[528] M. C. Genovese , M. E. Weinblatt , J. A. Aelion , et al., “ABT‐122, a Bispecific Dual Variable Domain Immunoglobulin Targeting Tumor Necrosis Factor and Interleukin‐17A, in Patients with Rheumatoid Arthritis with an Inadequate Response to Methotrexate: A Randomized,” Double‐Blind Study Arthritis & Rheumatology 70, no. 11 (2018): 1710–1720. 29855172 10.1002/art.40580 PMC6704363

[529] E. Yuba , E. Budina , K. Katsumata , et al., “Suppression of Rheumatoid Arthritis by Enhanced Lymph Node Trafficking of Engineered Interleukin‐10 in Murine Models,” Arthritis & Rheumatology 73, no. 5 (2021): 769–778. 33169522 10.1002/art.41585 PMC11095083

[530] T. Takeuchi , Y. Tanaka , S. Soen , et al., “Effects of the Anti‐RANKL Antibody Denosumab on Joint Structural Damage in Patients With Rheumatoid Arthritis Treated With Conventional Synthetic Disease‐modifying Antirheumatic Drugs (DESIRABLE study): A Randomised, Double‐blind, Placebo‐controlled Phase 3 Trial,” Annals of the Rheumatic Diseases 78, no. 7 (2019): 899–907. 31036625 10.1136/annrheumdis-2018-214827 PMC6585575

[531] Y. Chi , Y. Li , Q. Cheng , et al., “Efficacy and Safety of narlumosbart, an Anti‐RANKL Monoclonal Antibody, in Postmenopausal Women With Osteoporosis: A Multi‐center, Randomized, Double‐blind, Placebo‐ and Active‐controlled, Phased II Study,” EClinicalMedicine 85 (2025): 103329. 40686685 10.1016/j.eclinm.2025.103329 PMC12271762

[532] F. Eckstein , M. C. Hochberg , H. Guehring , et al., “Long‐term Structural and Symptomatic Effects of Intra‐articular Sprifermin in Patients With Knee Osteoarthritis: 5‐year Results From the FORWARD Study,” Annals of the Rheumatic Diseases 80, no. 8 (2021): 1062–1069. 33962962 10.1136/annrheumdis-2020-219181 PMC8292562

[533] H. A. Blair and E. D. Deeks , “Abatacept: A Review in Rheumatoid Arthritis,” Drugs 77, no. 11 (2017): 1221–1233. 28608166 10.1007/s40265-017-0775-4

[534] A. Hosseini , T. Gharibi , F. Marofi , Z. Babaloo , and B. Baradaran , “CTLA‐4: From Mechanism to Autoimmune Therapy,” International Immunopharmacology 80 (2020): 106221. 32007707 10.1016/j.intimp.2020.106221

[535] A. P. Cope , M. Jasenecova , J. C. Vasconcelos , et al., “Abatacept in Individuals at High Risk of Rheumatoid Arthritis (APIPPRA): A Randomised, Double‐blind, Multicentre, Parallel, Placebo‐controlled, Phase 2b Clinical Trial,” Lancet 403, no. 10429 (2024): 838–849. 38364839 10.1016/S0140-6736(23)02649-1

[536] J. Rech , K. Tascilar , M. Hagen , et al., “Abatacept Inhibits Inflammation and Onset of Rheumatoid Arthritis in Individuals at High Risk (ARIAA): A Randomised, International, Multicentre, Double‐blind, Placebo‐controlled Trial,” Lancet 403, no. 10429 (2024): 850–859. 38364841 10.1016/S0140-6736(23)02650-8

[537] J. Tuttle , E. Drescher , J. A. Simon‐Campos , et al., “A Phase 2 Trial of Peresolimab for Adults With Rheumatoid Arthritis,” New England Journal of Medicine 388, no. 20 (2023): 1853–1862. 37195941 10.1056/NEJMoa2209856

[538] Q. Tang , X. Zhang , X. Zhu , et al., “Camrelizumab in Combination With Doxorubicin, Cisplatin, Ifosfamide, and Methotrexate in Neoadjuvant Treatment of Resectable Osteosarcoma: A Prospective, Single‐arm, Exploratory Phase II Trial,” Cancer Medicine 13, no. 18 (2024): e70206. 39324173 10.1002/cam4.70206 PMC11424980

[539] K. Boye , A. Longhi , T. Guren , et al., “Pembrolizumab in Advanced Osteosarcoma: Results of a Single‐arm, Open‐label, Phase 2 Trial,” Cancer Immunology, Immunotherapy 70, no. 9 (2021): 2617–2624. 33580363 10.1007/s00262-021-02876-w PMC8360887

[540] D. M. Schwartz , Y. Kanno , A. Villarino , M. Ward , M. Gadina , and J. J. O'Shea , “JAK Inhibition as a Therapeutic Strategy for Immune and Inflammatory Diseases,” Nature Reviews Drug Discovery 16, no. 12 (2017): 843–862. 29104284 10.1038/nrd.2017.201

[541] J. Liu , Y. Jiang , S. Zhang , et al., “Ivarmacitinib, a Selective Janus Kinase 1 Inhibitor, in Patients With Moderate‐to‐severe Active Rheumatoid Arthritis and Inadequate Response to Conventional Synthetic DMARDs: Results From a Phase III Randomised Clinical Trial,” Annals of the Rheumatic Diseases 84, no. 2 (2025): 188–200. 39919893 10.1136/ard-2024-226385

[542] M. F. Robinson , N. Damjanov , B. Stamenkovic , et al., “Efficacy and Safety of PF‐06651600 (Ritlecitinib), a Novel JAK3/TEC Inhibitor, in Patients with Moderate‐to‐Severe Rheumatoid Arthritis and an Inadequate Response to Methotrexate,” Arthritis & Rheumatology 72, no. 10 (2020): 1621–1631. 32419304 10.1002/art.41316 PMC7589242

[543] M. C. Genovese , K. Kalunian , J. E. Gottenberg , et al., “Effect of Filgotinib vs Placebo on Clinical Response in Patients with Moderate to Severe Rheumatoid Arthritis Refractory to Disease‐Modifying Antirheumatic Drug Therapy: The FINCH 2 Randomized Clinical Trial,” Jama 322, no. 4 (2019): 315–325. 31334793 10.1001/jama.2019.9055 PMC6652745

[544] Y. Yazici , T. E. McAlindon , A. Gibofsky , et al., “A Phase 2b Randomized Trial of lorecivivint, a Novel Intra‐articular CLK2/DYRK1A Inhibitor and Wnt Pathway Modulator for Knee Osteoarthritis,” Osteoarthritis and Cartilage 29, no. 5 (2021): 654–666. 33588087 10.1016/j.joca.2021.02.004

[545] P. D. Miller , J. D. Adachi , B. H. Albergaria , et al., “Efficacy and Safety of Romosozumab among Postmenopausal Women with Osteoporosis and Mild‐to‐Moderate Chronic Kidney Disease,” Journal of Bone and Mineral Research 37, no. 8 (2022): 1437–1445. 35466448 10.1002/jbmr.4563 PMC9544335

[546] K. G. Saag , J. Petersen , M. L. Brandi , et al., “Romosozumab or Alendronate for Fracture Prevention in Women With Osteoporosis,” New England Journal of Medicine 377, no. 15 (2017): 1417–1427. 28892457 10.1056/NEJMoa1708322

[547] E. F. Eriksen , R. W. Boyce , Y. Shi , et al., “Reconstruction of Remodeling Units Reveals Positive Effects After 2 and 12 Months of romosozumab Treatment,” Journal of Bone and Mineral Research 39, no. 6 (2024): 729–736. 38640512 10.1093/jbmr/zjae055

[548] W. D. Tap , V. M. Villalobos , G. M. Cote , et al., “Phase I Study of the Mutant IDH1 Inhibitor Ivosidenib: Safety and Clinical Activity in Patients with Advanced Chondrosarcoma,” Journal of Clinical Oncology 38, no. 15 (2020): 1693–1701. 32208957 10.1200/JCO.19.02492 PMC7238491

[549] D. Fiorentino , A. R. Mangold , V. P. Werth , et al., “Efficacy, Safety, and Target Engagement of dazukibart, an IFNbeta Specific Monoclonal Antibody, in Adults With Dermatomyositis: A Multicentre, Double‐blind, Randomised, Placebo‐controlled, Phase 2 Trial,” Lancet 405, no. 10473 (2025): 137–146. 39798982 10.1016/S0140-6736(24)02071-3

[550] Y. Wang , Y. Kong , J. Du , et al., “Injection of human Umbilical Cord Mesenchymal Stem Cells Exosomes for the Treatment of Knee Osteoarthritis: From Preclinical to Clinical Research,” Journal of Translational Medicine 23, no. 1 (2025): 641. 40500748 10.1186/s12967-025-06623-y PMC12153132

[551] N. Mensali , H. Koksal , S. Joaquina , et al., “ALPL‐1 Is a Target for Chimeric Antigen Receptor Therapy in Osteosarcoma,” Nature Communications 14, no. 1 (2023): 3375. 10.1038/s41467-023-39097-x PMC10250459 37291203

[552] J. H. Chen , C. L. Zhao , J. Zhang , et al., “Enhancing Immunogenicity and Release of in Situ‐generated Tumor Vesicles for Autologous Vaccines,” Journal of Control Release 381 (2025): 113614. 10.1016/j.jconrel.2025.113614 40068738

[553] J. Pang , L. Huang , Y. Lian , Z. Yuan , F. Wang , and L. M. Zhang , “Co‐delivery of siAEG‐1 and doxorubicin to Treat Osteosarcoma via Nanomicelles for Azide‐alkyne "Click" Conjugation of Poly(l‐lysine) Dendrons Onto Zein,” International Journal of Biological Macromolecules 264, no. Pt 2 (2024): 130729. 38460643 10.1016/j.ijbiomac.2024.130729

[554] M. Guo , H. Liu , Y. Yu , et al., “Lactobacillus Rhamnosus GG Ameliorates Osteoporosis in Ovariectomized Rats by Regulating the Th17/Treg Balance and Gut Microbiota Structure,” Gut Microbes 15, no. 1 (2023): 2190304. 36941563 10.1080/19490976.2023.2190304 PMC10038048

[555] N. Aryaeian , M. Hadidi , M. Mahmoudi , M. Asgari , Z. S. Hezaveh , and S. K. Sadehi , “The Effect of Black Barberry Hydroalcoholic Extract on Immune Mediators in Patients With Active Rheumatoid Arthritis: A Randomized, Double‐blind, Controlled Clinical Trial,” Phytotherapy Research 35, no. 2 (2021): 1062–1068. 32914483 10.1002/ptr.6874

[556] S. Qdaisat , B. Wummer , B. D. Stover , et al., “Sensitization of Tumours to Immunotherapy by Boosting Early Type‐I Interferon Responses Enables Epitope Spreading,” Nature Biomedical Engineering 9, no. 9 (2025): 1437–1452. 10.1038/s41551-025-01380-1 40681861

[557] T. W. Laetsch , K. Ludwig , P. M. Williams , et al., “Phase II Study of Samotolisib in Children and Young Adults with Tumors Harboring Phosphoinositide 3‐Kinase/Mammalian Target of Rapamycin Pathway Alterations: Pediatric MATCH APEC1621D,” JCO Precision Oncology 8 (2024): e2400258. 39298693 10.1200/PO.24.00258 PMC11581706

[558] Y. Chen , Q. W. Chen , F. S. Fu , H. Y. Gu , A. Yu , and X. Z. Zhang , “Bone Destruction‐Chemotactic Osteoprogenitor Cells Deliver Liposome Nanomedicines for the Treatment of Osteosarcoma and Osteoporosis,” ACS Nano 18, no. 43 (2024): 29864–29879. 39424791 10.1021/acsnano.4c10053

[559] R. J. Miron , N. E. Estrin , A. Sculean , and Y. Zhang , “Understanding Exosomes: Part 2‐Emerging Leaders in Regenerative Medicine,” Periodontology 2000 94, no. 1 (2024): 257–414. 38591622 10.1111/prd.12561

[560] G. Bellini , D. Di Pinto , C. Tortora , et al., “The Role of Mifamurtide in Chemotherapy‐induced Osteoporosis of Children With Osteosarcoma,” Current Cancer Drug Targets 17, no. 7 (2017): 650–656. 27993113 10.2174/1568009616666161215163426

[561] U. M. Pirker‐Fruhauf , J. Friesenbichler , E. C. Urban , B. Obermayer‐Pietsch , and A. Leithner , “Osteoporosis in Children and Young Adults: A Late Effect After Chemotherapy for Bone Sarcoma,” Clinical Orthopaedics and Related Research 470, no. 10 (2012): 2874–2885. 22806259 10.1007/s11999-012-2448-7 PMC3441998

[562] Y. U. Chen , S. F. Xu , M. Xu , and X. C. Yu , “Postoperative Infection and Survival in Osteosarcoma Patients: Reconsideration of Immunotherapy for Osteosarcoma,” Molecular and Clinical Oncology 3, no. 3 (2015): 495–500. 26137256 10.3892/mco.2015.528 PMC4471531

